# Understanding of the impact of chemicals on amphibians: a meta-analytic review

**DOI:** 10.1002/ece3.249

**Published:** 2012-07

**Authors:** Andrés Egea-Serrano, Rick A Relyea, Miguel Tejedo, Mar Torralva

**Affiliations:** 1 Facultad de Biología, Departamento de Zoología y Antropología Física, Universidad de Murcia30100 Murcia, Spain; 2 Department of Biological Sciences, University of Pittsburgh101 Clapp Hall, Pittsburgh, Pennsylvania 15260; 3 Department of Evolutionary Ecology, Estación Biológica de DoñanaCSIC, Avda. Américo Vespucio s/n, 41092 Sevilla, Spain

**Keywords:** Amphibians, ecotoxicology, meta-analysis, phylogenetic signal, publication bias, synergism

## Abstract

Many studies have assessed the impact of different pollutants on amphibians across a variety of experimental venues (laboratory, mesocosm, and enclosure conditions). Past reviews, using vote-counting methods, have described pollution as one of the major threats faced by amphibians. However, vote-counting methods lack strong statistical power, do not permit one to determine the magnitudes of effects, and do not compare responses among predefined groups. To address these challenges, we conducted a meta-analysis of experimental studies that measured the effects of different chemical pollutants (nitrogenous and phosphorous compounds, pesticides, road deicers, heavy metals, and other wastewater contaminants) at environmentally relevant concentrations on amphibian survival, mass, time to hatching, time to metamorphosis, and frequency of abnormalities. The overall effect size of pollutant exposure was a medium decrease in amphibian survival and mass and a large increase in abnormality frequency. This translates to a 14.3% decrease in survival, a 7.5% decrease in mass, and a 535% increase in abnormality frequency across all studies. In contrast, we found no overall effect of pollutants on time to hatching and time to metamorphosis. We also found that effect sizes differed among experimental venues and among types of pollutants, but we only detected weak differences among amphibian families. These results suggest that variation in sensitivity to contaminants is generally independent of phylogeny. Some publication bias (i.e., selective reporting) was detected, but only for mass and the interaction effect size among stressors. We conclude that the overall impact of pollution on amphibians is moderately to largely negative. This implies that pollutants at environmentally relevant concentrations pose an important threat to amphibians and may play a role in their present global decline.

## Introduction

The negative impact of anthropogenic activities on bio-diversity is becoming increasingly conspicuous and amphibians are currently the most globally threatened group of vertebrates (approximately 41% of all species [Hoffmann et al. 2010]). Emergent diseases, habitat destruction, introduction of exotic species, and the pollution of both terrestrial and aquatic habitats have all been described as important threatening factors (Stuart et al. 2004; Wake and Vredenburgh 2008). Given these identified threats, it is critical that we determine the influence and magnitude of their effects on amphibian populations to help develop proper management and conservation strategies.

A variety of pollutants occur in natural habitats including fertilizers, pesticides, heavy metals, and road deicers. This broad array of pollutants is increasingly introduced into the environment by direct application, runoff from crop and forest applications or mines, urban and industrial sewage, and atmospheric deposition (Vitousek et al. 1997; Linder and Grillitsch 2000; Sparling 2000; Ritter and Bergstrom 2001). In short, the presence of pollutants is widespread (Carpenter et al. 1998; Kolpin et al. 2002; Gilliom et al. 2007) and is expected to increase in the near future (Tilman et al. 2001; Galloway et al. 2003). What we lack is an overall assessment of how different types of pollutants affect amphibians.

Documented effects of pollutants on amphibians range from lethal effects to sublethal effects including decreased growth and development and increased developmental abnormality frequency, susceptibility to diseases, and behavioral alterations (e.g., Bridges 1999; Ortiz et al. 2004; Relyea 2005; Griffis-Kyle 2007; Karraker et al. 2008; Shinn et al. 2008; Snodgrass et al. 2008; Relyea 2009). Due to the great diversity of pollutants and their modes of action, it is not surprising that they affect amphibians differentially. Additionally, several factors including pathogenic organisms and ultraviolet-B radiation are increasingly common in natural environments (Daszak et al. 2001; McKenzie et al. 2003) and these stressors can interact with chemical pollutants (e.g., Hatch and Blaustein 2000; Romansic et al. 2006; Macías et al. 2007). As a result, analyzing patterns in how these stressors interact with pollutants is of great relevance when considering the effects of pollution on amphibian populations (Hayes et al. 2010).

To date, a large proportion of studies examining the effects of pollutants on amphibians has been performed under laboratory conditions whereas fewer have been conducted under more natural conditions, such as outdoor mesocosms (Boone and James 2005). Although laboratory studies may use ecologically relevant concentrations, outcomes observed under such conditions may not be applicable to more natural conditions (Boone and Bridges 2003) because actual concentrations in the environment can be affected by several factors including plant uptake, denitrification, and sediment trapping (e.g., Ritter and Bergstrom 2001), or because the aforementioned interaction among stressors may mask the effect of a given chemical. As a result, it is possible that laboratory studies overestimate or underestimate the impact of chemical pollutants on amphibians (Boone and Bridges 2003; Gómez-Mestre and Tejedo 2003). This emphasizes the need to compare the effects of pollutants on amphibians across experimental venues (Skelly 2002, but see Chalcraft et al. 2005).

It is also reasonable to hypothesize that there may be important species- or family-level differences in sensitivity, although studies to date have been equivocal on this point (Marco et al. 1999; Bridges and Semlitsch 2000; Shinn et al. 2008; Snodgrass et al. 2008; Jones et al. 2009). What is needed is an examination of pollutant effects within a phylogenetic framework. The impact of pollutants may also vary with the developmental stage at which individuals are initially exposed (Bridges 2000; Greulich and Pflugmacher 2003; Griffis-Kyle 2005; Ortiz-Santaliestra et al. 2006). Collectively, it is clear that to fully understand the effects of pollutants on amphibians, we need to consider a wide variety of factors including type of pollutant, the presence of additional stressors, experimental venue, phylogenetic relationships, and ontogenetic stage.

Several reviews have been published examining the effects of different pollutants on amphibians (Cowman and Mazanti 2000; Linder and Grillitsch 2000; Sparling 2000; Camargo et al. 2005; Relyea and Hoverman 2006; Marco and Ortiz-Santaliestra 2009; Kerby et al. 2010). These reviews have taken the approach of summarizing studies by the method of vote counting (counting the number of significant vs. nonsignificant outcomes) or by simply summarizing LC_50_ values studies of pollutants on amphibian survival (where LC_50_ is the lethal concentration that kills 50% of a population). In LC_50_ reviews, the summarized studies are largely restricted to single-species lab studies (i.e., individuals extracted from their natural environment) and survival is the only response variable. Moreover, since the analysis of LC_50_ values likely bias the actual impact of chemicals on amphibians in natural environments (LC_50_ values often are much higher than actual concentrations in the field; e.g., Egea-Serrano et al. 2009a), more information on the impact of ecologically relevant concentrations on survival and sublethal endpoints is essential to determine properly the sensitivity of amphibians to chemical pollution. In vote-counting studies, there are always concerns that the conclusions obtained may not be correct (e.g., due to low sample size) and that the estimates may be highly biased since they have poor statistical power (Rosenberg et al. 2000). In addition, vote counting does not provide a reliable way to determine the magnitude of the effect and compare responses among predefined groups (Gurevitch et al. 2000).

An alternative methodology to averaging LC_50_ studies or using vote counting is the use of meta-analytic techniques. Meta-analytic techniques incorporate the magnitude of effects and the sample size of each study to derive test statistics of overall effect sizes. Meta-analyses can also compare effect sizes among predefined groups, including phylogenetic groups, types of environmental manipulations, and experimental venues. These techniques can also be used to examine 2 × 2 factorial manipulations (Gurevitch et al. 2000). For example, meta-analyses have recently been used to examine the overall effect of ultraviolet-B radiation on amphibians and other aquatic organisms, as well as its interaction with other environmental factors (Bancroft et al. 2007, 2008).

Using meta-analytic techniques, the objectives of our study were the following: (1) to determine the overall effect of environmentally relevant concentrations of some chemical pollutants on amphibian survival, mass, developmental time, and abnormality frequency; (2) to assess the interactive effects of pollutants and other stressors on amphibians; (3) and to determine whether there are significant differences in pollution effects among predefined groups (i.e., amphibian lineages, experimental venues, developmental stages, and types of pollutants) for the abovementioned response variables.

## Material and Methods

### Data collection

We used three methods to identify the studies to include in the meta-analysis. First, we searched four electronic databases (ISI Web of Science, BIOSIS Previews, ScienceDirect, Scirus) using 10 search words (fertilizers, pesticides, heavy metals, wastewater contaminants, deicers, poly-chlorinated byphenyls, hydrocarbons, dioxins, furans, and estrogens) for dates prior to December 2008. These search words are considered to include the major types of chemicals affecting amphibians (e.g., Sparling et al. 2000). Second, we examined the citations from the studies dealing with amphibians resulting from the abovementioned search, especially from two recent reviews (Camargo et al. 2005; Marco and Ortiz-Santaliestra 2009). From all of the studies obtained, we only included data in the meta-analysis if they met the following criteria: (1) the studies reported data on amphibian survival at the end of the experiments, time to hatching, time to metamorphosis, mass at the end of the experiments, or abnormality frequency at the end of the experiments; (2) the studies stated that concentrations used in the experiments were ecologically relevant (i.e., found in natural water bodies, regardless of whether they are unusual or typical concentrations, as reported by the authors of the original studies in their publications or in personal communications); (3) the studies clearly specified how many days the experiments lasted; (4) the studies provided means, sample sizes, and measures of variance (i.e., standard deviation, SD, or standard error, SE) for both a control group (i.e., not exposed to contaminant) and an experimental group (i.e., exposed to contaminant); (5) the studies reported the effect for a pollutant in isolation and not in combination with other factors (e.g., a pollutant combined with resource competition, predators, etc.); (6) in the case that a published study reported data for more than one species, population, pollutant, or pollutant concentration, each outcome was considered to be independent in the meta-analyses. When a given study showed the effects of both realistic and nonrealistic concentrations, data were filtered to exclude the latter ones. Therefore, all concentrations considered in the present study were ecologically relevant. Since studies dealing with polychlorinated byphenyls, hydrocarbons, dioxins, furans, and estrogens, and meeting all the abovementioned criteria were not found, the meta-analysis could not be performed on these types of chemicals.

For each study, we obtained the mean, SD, and sample size (*n*) for both the control and the experimental group. When means and measures of variance were presented graphically, we digitized the graph to estimate the values (ImageProPlus version 4.5.0.29 for Windows). If SEs were reported, these values were transformed into SD according to the equation: SD = SE·√*n*. For those studies that did not clearly include the required data, we attempted to contact authors to obtain the data. In addition, for each study we also compiled information regarding family, developmental stage, experimental venue, and type of pollutant.

To assess the effect of pollutants combined with other stressors (both biotic and abiotic), we conducted a factorial meta-analysis (Gurevitch et al. 2000). The factorial meta-analysis examines the magnitude of the effect of the two main factors and their interaction. Data used in the factorial meta-analysis came from publications meeting the above criteria and also showing a 2 × 2 factorial structure (Gurevitch et al. 2000). The original objective was to examine the effect of the interaction between pollutants and other stressors that eventually could include a second pollutant. Consequently, we used a *first group of stressing factors* (STRESS-1) (nitrogenous compounds, pesticides, and wastewater pollutants) and a *second group of stressing factors* (STRESS-2) (competitors, pH, predators [either caged or uncaged], ultraviolet radiation, other wastewater pollutants, and mold). Other types of factors or combinations among chemicals were not included in the analysis because of the scarcity of studies meeting all the selection criteria. When for a given study both groups of stressing factors involved wastewater pollutants, each pollutant was arbitrarily assigned only to either STRESS-1 or STRESS-2. Therefore, each comparison was included only once in the calculation of effect size. Due to the scarcity of experiments addressing the impact of the interaction among pollutants and other stressors for most response variables, the factorial meta-analysis could only be performed on amphibian survival.

### Data analysis

#### Meta-analysis

For all studies reporting the impact of a control treatment (pollutant absent) and at least one polluted treatment (pollutant present) in the absence of other stressors, we used Hedge's *d*^+^ as the metric of standardized effect size. Hedge's *d*^+^ provides a measure of the overall magnitude of the treatment effect while adjusting for small sample sizes (Rosenberg et al. 2000). Since the absence of pollutant was considered as control, negative effect sizes would indicate reduced survival, mass, time to hatching, time to metamorphosis, or abnormality frequency related to pollutant exposure. To calculate Hedge's *d*^+^ for each study, mean, SD, and *n* for both the control and the experimental group were used after dividing mean and SD by the number of days that the corresponding experiments lasted. This approach allowed us to correct for differences among studies in the duration of exposure. Since for most studies and traits data were reported only for the end of the experiment, a linear evolution of the effect of pollutants over time had to be assumed. This transformation was applied to all studied traits, except for time to hatching and time to metamorphosis, which explicitly consider duration of exposure. Since the results obtained after conducting the analyses described below on corrected and uncorrected Hedge's *d*^+^ were qualitatively similar, results for uncorrected data are not reported in the present study.

For each trait, data were analyzed using categorical random effects models to calculate the grand mean effect size. Additionally, any difference among predefined groups was analyzed using mixed-effects models. Such groups included: (1) amphibian family; (2) developmental stage at the time of pollutant application (embryonic, larval, or postmetamorphic individuals); (3) experimental venue (laboratory, mesocosm, or field enclosures, as well whether the animals were collected in the field [i.e., field experiments]); and (4) type of pollutant (nitrogenous compounds, phosphorous compounds, pesticides, road deicers, heavy metals, and other wastewater contaminants [i.e., perchlorate, boron, acetaminophen, caffeine, and triclosan]). When mixed-effects models were conducted, we calculated mean effect sizes and 95% confidence limits for each class. Additionally, heterogeneity statistics were calculated to quantify between-group (*Q*_B_) and within-group (*Q*_W_) variation. Effect sizes were considered significant if the 95% confidence intervals did not cross zero. The magnitude of the overall effect size is generally interpreted as “small” if *d*^+^ = 0.2, “medium” if *d*^+^ = 0.5 and “large” if *d*^+^≥ 0.8 (Cohen 1969). Effect sizes within analyses (e.g., nitrogenous compounds effect vs. pesticides effect) were considered different from one another if their 95% confidence intervals did not overlap. All statistical analyses were performed using MetaWin 2.1 statistical program (Rosenberg et al. 2000). This program eliminates groups with fewer than two valid cases from the analyses when running mixed-effects models. Therefore, mean and 95% confidence interval could not be reported for some of the categories included in the categorical random effect models performed to calculate grand mean effect sizes.

#### Factorial meta-analysis

As in the previous analyses, we calculated Hedge's *d*^+^ standardized effect size and its corresponding sampling variance following Gurevitch et al. (2000). For each study, the following effect sizes were estimated: (1) the average effect of the exposure to STRESS-1; (2) the average effect of the exposure to STRESS-2; (3) their interaction (STRESS-1 × STRESS-2).

#### Phylogenetic comparative analysis

To determine whether effects of pollutants were significantly associated with amphibian phylogeny, we conducted tests for serial independence (TFSI) on continuous characters (Abouheif 1999) for each response variable, for both simple and factorial meta-analyses. The diagnosis is based on a measurement of the autocorrelation of a trait across phylogeny, in the form of a *C*-statistic, resulting from similarity between adjacent phylogenetic observations. The topology and associated numerator distribution were randomized 2000 times and the *C*-statistic was calculated for each randomized topology to build the null hypothesis. The observed *C*-statistic was compared to the randomized distribution to calculate its level of significance. Significant phylogenetic autocorrelation was defined when the observed *C-*statistic falls to the right of the distribution of the randomized *C-*statistics and *P*≤ 5% (Abouheif 1999). Such a result would imply that related species show similar responses regardless of the effect of pollutants.

To conduct the phylogenetic analyses, a topology was constructed following Frost et al. (2006), combined with additional detail from several family phylogenetic assessments: Salamandridae (Zajc and Arntzen 2000; Weisrock et al. 2006; Zhang et al. 2008), Ambystomatidae (Shaffer et al. 1991; Jones et al. 1993), Pelobatidae and Pelodytidae (García-París et al. 2003), Myobatrachidae (Schäuble et al. 2000; Read et al. 2001), Hylidae (Faivovich et al. 2005), Bufonidae (Pauly et al. 2004), and Ranidae (Veith et al. 2003; Hillis and Wilcox 2005; Scott 2005). Branch length information was not available for our composite phylogenies, but all analyses performed can be conducted using only topology without knowledge of branch lengths. Before conducting the phylogenetic analyses, we assigned the same value to all branch lengths and a single effect size was calculated for each species. The phylogenetic comparative analyses were conducted after replacing the names of the different species at the tip of each branch in the topology constructed by the corresponding single effect sizes. Such effect sizes were the tip data (Abouheif 1999) to which TFSI were applied.

#### Publication bias

To assess whether publication bias existed for the datasets used in our study (defined as the selective publication of studies reporting certain types of results over those showing other results; Begg 1994), different approaches were used. For each response variable, for both simple and factorial meta-analyses, we calculated Rosenberg's fail-safe number using a fail-safe number calculator that is applicable to random-effect models (Rosenberg 2005; http://www.public.asu.edu/~mrosenb/software.html#failsafe). Rosenberg's fail-safe number is the number of nonsignificant, unpublished, or missing studies that would need to be added to a meta-analysis to change the results from significant to non-significant (Rosenberg 2005). The results obtained are considered robust when the fail-safe number is larger than 5*n*+ 10 (where *n* = the number of studies; Rosenberg 2005). Additionally, we used the funnel plot technique and Spearman's rank correlation to determine the relationships between effect size and sample size for each response variable. If selective reporting is absent, plots of effect size against sample size should fit a funnel pattern and Spearman's rank correlations should not be significant. All three procedures were used to test for publication bias (Begg and Mazumdar 1994; Palmer 1999).

## Results

Among the published studies examined that met our data selection criteria, only 48 were suitable (Table S1). However, not all studies provided data for all response variables. As a result, survival and mass had more data available (survival: 35 studies, 256 point samples; mass: 19 studies, 181 point samples) than time to hatching (three studies, 23 point samples), time to metamorphosis (nine studies, 37 point samples), and abnormality frequency (four studies, 33 point samples; Table S1). For the factorial meta-analysis of survival, only 12 published studies met our data criteria and these 12 studies provided 45 datapoints (Table S1).

### Meta-analysis

#### Survival

**Figure 1:**
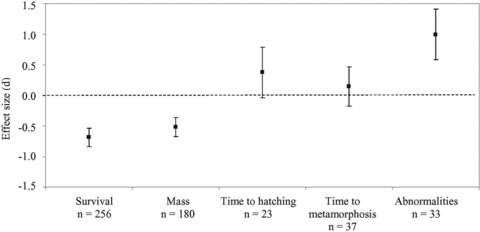
Effect size (mean and 95% confidence interval) for full models for the effect of pollutants on amphibian survival, mass, time to hatching, time to metamorphosis, and abnormality frequency. The number of point samples used to calculate each mean is shown. Means with confidence intervals that overlap the line at zero are not significantly different from zero.

**Figure 2:**
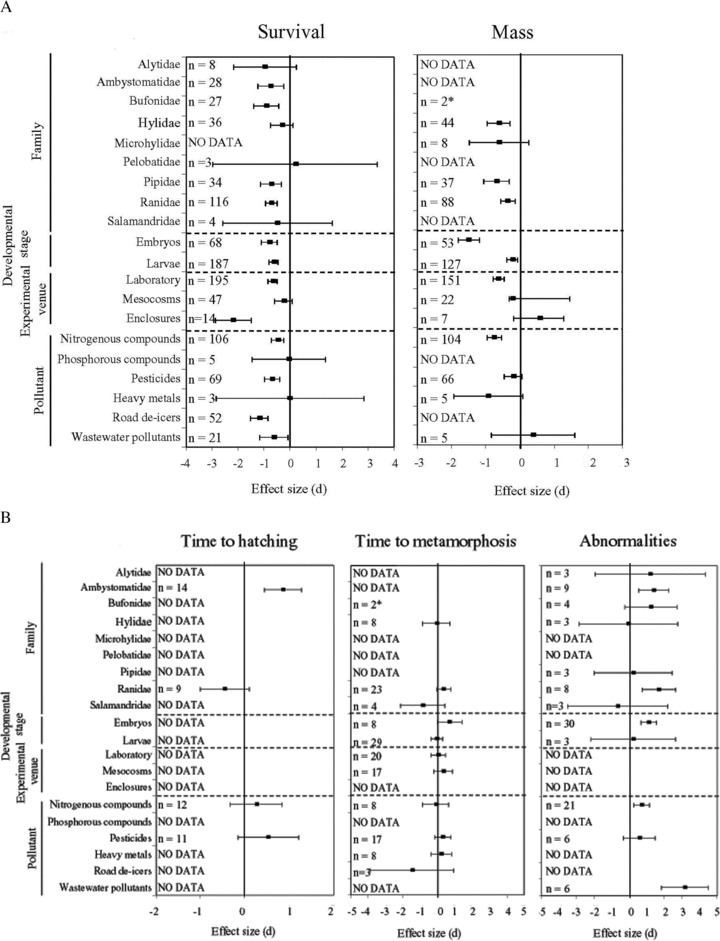
Effect (mean and 95% confidence interval) of pollutants on survival, mass, time to hatching, time to metamorphosis, and abnormality frequency for the categories considered for the a priori defined groups. The number of point samples used to calculate each mean is shown for each analysis. The expression “no data” denotes both those classes for which actually no data were available and those for which fewer than two valid studies (see Material and Methods for more detail). Effect sizes were considered significant if 95% confidence intervals did not overlap with zero. Effect sizes within analyses were considered different from one another if their 95% confidence intervals did not overlap. Notice the different scales for each variable shown. *For clarity, statistics corresponding to categories showing small sampling size (*n*≤ 2) are not shown in the graphic.

Survival was significantly heterogenous (i.e., not all effect sizes were equal) (Table 1). Across all studies, pollutants had a medium (0.5 < *d* < 0.8) negative effect on survival (Fig. 1), which translated to a 14.3% (±1.7 SE) decrease in survival. Ambystomatidae, Bufonidae, Pipidae, and Ranidae families showed significantly reduced survival (Fig. 2), but we found no significant differences either among families or developmental stages. Conversely, significant differences in survival were detected for experimental venue. Although survival was reduced in laboratory and enclosure conditions, the decline in laboratory studies was of medium magnitude whereas the decline in enclosure studies was large. The type of pollutant also mattered; road deicers were more lethal than nitrogenous compounds whereas pesticides and waste-water pollutants caused an intermediate reduction in survival. Heavy metals and phosphorous compounds did not affect survival.

**Table 1:** Heterogeneity statistics for each model in the survival, mass, time to hatching, time to metamorphosis, and abnormality frequency analyses. NA, not applicable; df, degrees of freedom; BG, between groups (referring to the variation in effect size explained by the model, *Q*_B_). For clarity, the residual error heterogeneity (*Q*_W_) corresponding to the different statistical models is not shown. With the exception of time to hatching and time to metamorphosis (for all the models) and abnormality frequency (only for family and pollutant models), the residual error heterogeneity was significant, which implies that there is still heterogeneity among effect sizes not explained by the model (Rosenberg et al. 2000)

	Survival (*n* = 270)			Mass (*n* = 187)			Time to hatching (*n* = 23)		
			
Statistical model	df	*Q*	*P*	df	*Q*	*P*	df	*Q*	*P*
Full model (no structure)	**255**	**445.042**	**0.000001**	**179**	**215.491**	**0.032**	22	22.427	0.435
Family^BG^	7	6.222	0.514	**4**	**10.002**	**0.040**	**1**	**17.747**	**0.00003**
Developmental stage^BG^	1	0.812	0.368	**1**	**54.294**	**0.000001**	NA	NA	NA
Experimental venue^BG^	**2**	**27.988**	**0.000001**	**2**	**12.516**	**0.002**	NA	NA	NA
Pollutant^BG^	**5**	**15.078**	**0.010**	**3**	**16.843**	**0.0008**	1	0.423	0.515

	**Time to metamorphosis (*n* = 37)**			**Abnormality frequency (*n* = 39)**					
					
Statistical model	df	*Q*	*P*	df	*Q*	*P*			
Full model (no structure)	36	42.054	0.225	**32**	**50.754**	**0.019**			
Family^BG^	**3**	**9.004**	**0.029**	**6**	**15.405**	**0.017**			
Developmental stage^BG^	**1**	**4.430**	**0.035**	1	2.184	0.139			
Experimental venue^BG^	1	0.793	0.373	NA	NA	NA			
Pollutant^BG^	**3**	**8.989**	**0.029**	**2**	**20.928**	**0.00003**			

#### Mass

Significant heterogeneity was detected for mass (Table 1). This response variable was significantly affected by pollutants, with an overall negative effect that was medium (*d*∼ 0.5) in size (7.5% mean decrease [±1.8 SE] across all studies) (Fig. 1). A significant family effect was also revealed. Hylidae, Pipidae, and Ranidae exhibited significantly reduced mass, with Pipidae having a smaller effect size than the other two families. Nevertheless, there were not significant pairwise differences between families, as revealed by the overlap of the confidence intervals (Fig. 2). Significant differences were also found among developmental stage, experimental venue, and type of pollutant. Mass was lower when the exposure to pollution occurred at embryonic stages, when experiments were conducted under laboratory conditions, and when the pollutant consisted of nitrogenous compounds.

#### Developmental time

Although both time to hatching (2.3% mean increase [±3.0 SE]) and time to metamorphosis (3.5% mean increase [±3.9 SE]) tended to increase across all studies, the overall analysis of these response variables revealed a lack of both significant heterogeneity and effect (Table 1; Fig. 1). However, we did detect significant differences among families in their time to hatching. Only Ambystomatidae exhibited a significant delay in hatching time when exposed to pollutants (Fig. 2). No significant differences in either development trait were detected for the other predefined groups (Fig. 2).

#### Abnormality frequency

The overall abnormality frequency was significantly heterogenous and increased when amphibians were exposed to pollutants, which had a large effect size (*d* > 0.8; Table 1; Fig. 1). This translated to a 535% mean increase (±287.7 SE) in abnormality frequency across all studies. Among the predefined groups, the only significant differences were among families and pollution types. Increased frequency of abnormalities was observed in the families Ambystomatidae and Ranidae, although the increase observed in these two families was not significantly different from the other families (Fig. 2). Abnormality frequency was affected most strongly by waste-water pollutants and, to a lesser extent, nitrogenous compounds. Though no significant developmental stage effect was detected, increased abnormality frequency was observed when pollutants were applied during embryonic stages.

### Factorial meta-analysis

**Figure 3:**
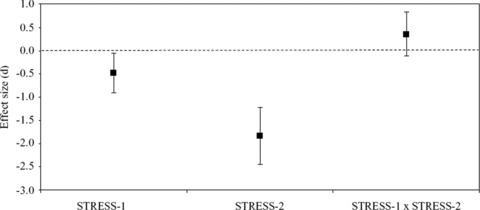
Effect size (mean and 95% confidence interval) for full models for the parameters calculated for the factorial meta-analysis of the effect of the interaction between pollutants and additional stressors on amphibian survival (*n* = 45). Effect sizes were considered significant if 95% confidence intervals did not overlap with zero. STRESS-1: average overall effect sizes of exposure to STRESS-1; STRESS-2: average overall effect sizes of exposure to STRESS-2; STRESS-1 × STRESS-2: average interaction effect size between the exposure to STRESS-1 and to STRESS-2.

In the factorial meta-analysis, which was only conducted on amphibian survival, we detected significant heterogeneity for STRESS-1 and STRESS-2 effect sizes (*P* < 0.0002 in all cases), but not for the interaction term STRESS-1 × STRESS-2 (*Q*_44_ = 45.45; *P* = 0.41). The average overall effect on survival of the exposure to the first group of stressors (nitrogenous compounds, pesticides, and wastewater pollutants; STRESS-1) was significantly weaker than the effect of the second group of stressors (competitors, pH, predators, ultraviolet radiation, other wastewater pollutants, and mold; STRESS-2; Fig. 3). The overall interaction effect size did not differ from zero (Fig. 3).

### Phylogenetic comparative analysis

The phylogenetic comparative analysis found weak evidence of phylogenetic patterns in sensitivity to pollutants. The TFSI detected significant phylogenetic autocorrelation among the tip data for the effect size in time to hatching (mean *C*-statistic = 0.461; *P* = 0.026). However, the test did not detect phylogenetic signal for the remaining response variables.

### Publication bias

The weighted Rosenberg's fail-safe number was large for survival (2304.9) and mass (992.0), whereas it was smaller for the rest of variables (<57.5 in all cases). For the factorial meta-analysis of survival, Rosenberg's fail-safe number was low for all the effect sizes analyzed (<35.8 in all cases).

We also correlated effect size and sample size using Spearman's rank correlation analyses to formally test for publication bias. We found no significant correlation for time to metamorphosis (*r*_s_ = 0.013; *P* = 0.939), time to hatching (*r*_s_ = –0.087; *P* = 0.693), abnormality frequency (*r*_s_ = 0.245; *P* = 0.169), or survival (*r*_s_ = 0.076; *P* = 0.229). For the factorial meta-analysis, no significant correlation was found for the overall effect size of exposure to STRESS-1 (*r*_s_ = 0.168; *P* = 0.270). The only significant correlations were for mass (*r*_s_ = 0.373; *P* = 0.0001) in the meta-analysis and for survival under the exposure to STRESS-2 (STRESS-2; *r*_s_ = –0.447; *P* = 0.002) and for the interaction effect size between the exposure to STRESS-1 and to STRESS-2 (*r*_s_ = 0.276; *P* = 0.039) in the subsequent factorial meta-analysis. Collectively, these results suggest that there was not publication bias for most of the response variables, although it cannot be ruled out in three of the eight cases tested.

## Discussion

The meta-analysis revealed that the exposure of amphibians to chemical pollutants as a group at environmentally relevant concentrations causes medium effect sizes on reduced survival and mass and a large effect on increased frequency of abnormalities. This overall analysis supports the conclusions derived from vote-counting reviews on the general effect of pollutants on amphibians (Cowman and Mazanti 2000; Linder and Grillitsch 2000; Sparling 2000; Camargo et al. 2005; Relyea and Hoverman 2006; Marco and Ortiz-Santaliestra 2009). Although changes caused by pollutants on survival and mass are lower than 15%, any reduction in these traits, or the presence of deformities, could reduce juvenile recruitment affecting amphibian population sizes (Hayes et al. 2010), which agrees with the hypothesis that pollution is one of the major threats faced by amphibians (e.g., Beebee and Griffiths 2005; Hayes et al. 2010). However, our meta-analytic approach highlighted the fact that the impact of pollution was primarily on survival, mass, and abnormality frequency and not on amphibian developmental time (i.e., time to hatching and to metamorphosis). Although some evidences of publication bias were detected for some of the variables analyzed, the effect of such a bias is possibly small. Therefore, the results obtained appear to be a reliable review of the published evidences on the impact of pollution on amphibians analyzed.

Although it can be difficult to know when negative effects on individuals may also affect population dynamics (e.g., Schmidt 2004), understanding how individuals are affected by such single and multiple stressors is essential because individual traits such as larval mortality may have deleterious effects at the population level (Gamradt and Kats 1996; Sih et al. 2004; Vredenburg 2004; Hayes et al. 2010). The overall effects of pollutants detected in the meta-analysis certainly could lead to population declines both directly by reducing individual survival and indirectly by decreasing mass or by increasing the frequency of abnormalities. We discuss these issues in more detail below.

### Survival

Embryonic and larval amphibian survival was affected by pollutants, although significant differences among experimental venues and types of pollutants were observed. The negative overall effect of pollutants on survival is due to physio-logical alterations, such as increased methemoglobin concentrations, modification of enzyme activities, and even DNA damage (e.g., Huey and Beitinger 1980a; Ralph and Petras 1997; Widder and Bidwell 2006).

Road deicers had a significantly larger effect relative to nitrogenous compounds. This fact may be due to the high electrical conductivity of water considered in those studies examining the effect of road deicers (e.g., Karraker 2007; Karraker et al. 2008). Although such conductivity levels were found in natural ponds, it is likely that, overall, they were much higher than in the case of pollution by nitrogenous compounds. Consequently, osmotic stress was more deadly than the effect of these latter pollutants. The lack of effect of phosphorous compounds on amphibians agrees with previous findings (Smith 2007, but see Hamer et al. 2004). However, the lack of effect of heavy metal exposure contradicts the conclusions of past studies (Linder and Grillitsch 2000), although this may be due to the small sample size and low statistical power incurring in type II errors. Moreover, the reported study revealed that the lack of effect of heavy metals on survival may be mediated by the elevated mortality in control treatment (Chen et al. 2006), also increasing type II errors. Further research is needed to verify the effects of heavy metals on amphibian survival.

The exposure to pollutants in field enclosure experiments exhibited higher mortality than in laboratory or mesocosms experiments. Since high salinity, acidity, or ultraviolet-B radiation, and even additional pollutants become additional stressors present in natural ponds and streams, these factors may be the cause of the higher mortality under enclosure conditions (e.g., Hatch and Blaustein 2000; Boone et al. 2005; Macías et al. 2007; Egea-Serrano et al. 2009b; Egea-Serrano 2010; Ortiz-Santaliestra et al. 2010).

Evidence of ontogenic variation in vulnerability to pollutants has been previously reported for individual pollutants (e.g., Bridges 2000; Bridges and Semlitsch 2000; Greulich and Pflugmacher 2003; Ortiz-Santaliestra et al. 2006). Differences in sensitivity to pollutants between embryos and larvae have often been attributed to the gelatinous egg matrix in which embryos live and the complete tissue and organ differentiation of larvae. The jelly coat may protect embryos from some chemicals (Berrill et al. 1998; Pauli et al. 1999), but it can react with other chemicals and become more toxic (Räsänen et al. 2003a; Marquis et al. 2006a). Incomplete development of the nervous system may protect embryos from those chemicals affecting nervous system (Ortiz-Santaliestra et al. 2006). Additionally, nitrate in the gut may be transformed into nitrosamines, which are carcinogenic (Committee on Nitrate Accumulation 1972), and symbiotic gut bacteria are involved in digestion (affecting food ingestion by larvae) and in the transformation of nitrate into nitrite (compound increasing methemoglobin concentration) (Huey and Beitinger 1980b; Hecnar 1995). Thus, incomplete gut differentiation can protect embryos from some chemicals. On the other hand, later larval stages may be more tolerant to chemicals than embryos because of increased detoxifying ability (Bucciarelli et al. 1999) and because of their internal gills (Duellman and Trueb 1994) and increased skin thickness, which protect them against osmoregulatory alterations (McDiarmid and Altig 1999). All these aspects did not make us to expect significant differences among developmental stages. Thus, while individual studies have found differences in tolerance among developmental stages, our analysis across all studies indicates there is not a general pattern of differential sensitivity to pollutants between embryos and larvae. Some of the original studies included in the analysis lasted only until embryos hatched. Consequently, carry-over effects were not taken into account, which may underestimate the observed effect when the exposure to pollutants started at embryonic stages. Therefore, future research identifying the effect of embryonic exposure on tadpole performance is relevant to establish the actual role of pollutants on amphibian population decline.

The general lack of variation in survival effects among amphibian families and weak phylogenetic signal disagrees with individual studies that have suggested the existence of a phylogenetic signal in the impact of particular pesticide or nitrogenous compound (e.g., Marco et al. 1999; Bridges and Semlitsch 2000; Shinn et al. 2008; Snodgrass et al. 2008; Jones et al. 2009; Relyea and Jones 2009). Thus, while some chemicals can have species-specific effects, when we combined all studies, we found no general phylogenetic pattern of sensitivity. However, the fact that some families (Alytidae, Pelobatidae, and Pipidae) were represented by a single species may contribute to explain the general lack of a pattern observed. Moreover, population-level variation in chemical tolerance can occur (e.g., Shinn et al. 2008; Egea-Serrano et al. 2009b) and high levels of such a variation may be obscuring species-level patterns. Indeed, this is an issue in any phylogenetic analysis and researchers commonly must work under the assumption that population-level variation is lower than species-level variation. To assess the magnitude of this potential problem, future research should examine both species- and population-level tolerance within a phylogenetic framework.

### Mass

Overall, pollutants significantly reduced amphibian mass, although this effect depended on developmental stage, experimental venue, and pollutant type. No significant differences among amphibian families were detected, which would support the lack of phylogenetic autocorrelation detected for this trait. As mentioned earlier, this nonsignificant effect could be influenced by that for families Microhylidae and Pipidae, all data came from a single species and/or interpopulation variation in tolerance, a factor that should be analyzed in future research.

The overall negative effect of pollutants on mass can be a combination of direct and indirect effects. Direct effects include reduced foraging efficiency or increased physiological stress due to detoxification pathways (Wright and Wrigth 1996; Egea-Serrano et al. 2009b). Indirect effects occur when pollutants may impact either positively or negatively mass by affecting algal growth in more realistic experimental venues (Boone et al. 2007; Relyea 2009; Egea-Serrano 2010).

Pollutant effect on mass was significantly more negative when the exposure began in embryonic stages than when it began in larval stages. Such effects may be mediated by simply longer exposure to pollutants when experiments began at earlier stages (mean ± 1 SE; embryos: 751 ± 107 h, *n* = 76; larvae: 446 ± 34 h, *n* = 286; *F*_1,360_ = 12.514; *P* = 0.0001) or may be due to a higher sensitivity of embryos to pollution or some lag effect that reduce their subsequent growth rates (Herkovits and Fernández 1978; McDiarmid and Altig 1999; Marquis et al. 2006a). Declines in mass, especially when amphibians are exposed as embryos, may subsequently result in additional detrimental effects by making individuals more vulnerable to gape-size predators for longer periods of time (Semlitsch and Gibbons 1988), reducing competitive abilities, increasing larval development duration (Snodgrass et al. 2004), or affecting future survival and fitness (Berven and Gill 1983; Smith 1987; Semlitsch et al. 1988; Reques and Tejedo 1997; Altwegg and Reyer 2003).

The smaller decline in mass observed in mesocosm and enclosure studies compared to lab studies may be attributed to the indirect effect of pollutants. Under the more realistic conditions of mesocosms and enclosures, these chemicals can affect algal growth (Boone et al. 2007; Relyea 2009; Egea-Serrano 2010) in ways that can positively and negatively affect amphibian growth. In addition, there is typically a reduction in larval crowding conditions in mesocosms and enclosures compared to laboratory studies, which should reduce competitive stress, and allow individuals to get more energy to invest in detoxification pathways. This reduction in density in mesocosms and enclosures may counterbalance negative effects of pollutants on mass, and weaken their impact, an outcome already suggested for other stressors such as predation risk (Peacor and Werner 2000).

A second explanation for the observed differences among experimental venues is that water is frequently changed and pollutants are reapplied in lab experiments. In contrast, mesocosm or enclosure experiments typically use a single application of pollutants. Therefore, pollutants in mesocosm and enclosure experiments have a greater opportunity to break down over time. As a result, amphibians in these venues should experience lower average exposures, resulting in less-severe effects on mass.

Across all studies, nitrogenous compounds generally reduced amphibian mass more than pesticides. This overall effect contradicts a number of individual studies reporting that ecologically relevant concentrations of some pesticides and nitrogenous compounds caused similar effects on mass (e.g., Boone et al. 2005; Boone and Bridges-Britton 2006). Nitrogenous compounds may increase water salinity (e.g., Egea-Serrano 2010) and lead to oxygen depletion because of eutrophication processes (see review Camargo and Alonso 2006). The interaction between the nitrogen toxicity and these additional stressing effects may exacerbate the effects of nitrogen toxicity isolated and reduce larval mass synergistically (e.g., Ortiz-Santaliestra 2008), which would explain the results obtained.

### Developmental time

Overall, pollutants did not affect time to hatching or time to metamophosis, although their effects varied among families. Although some individual studies have detected significant effect of pollutants on time to hatching (e.g., Ingermann et al. 1997; Räsänen et al. 2003a, b; Rohr et al. 2003, 2004; Griffis-Kyle 2007), many studies have reported no impact (Berrill et al. 1994, 1998; Berrill and Bertram 1997; Pauli et al. 1999; Greulich and Pflugmacher 2003; Griffis-Kyle 2007). The frequent lack of pollutant effect on time to hatching may be due to the protective role of the embryonic jelly coat (Räsänen et al. 2003c; Marquis et al. 2006b; Edginton et al. 2007) or incomplete embryonic organ development (Hecnar 1995). However, the hypothesized protective mechanisms were not effective for other response variables, including survival, suggesting that pathways involved in embryonic development do not affect other traits.

Significant heterogeneity among ambystomatids and ranids was observed in relation to time to hatching, which is supported by the significant autocorrelation detected for this variable. Ranids were unaffected by pollutants whereas ambystomatids delayed their embryonic development, likely due to the impact of chemicals on the rate of cellular division. The lack of effect of pollutants on time to hatching detected for ranids is consistent with the results obtained for time to metamorphosis for this family, the only one for which data for both variables were available. Although for some species larval period may be affected by pollutants (e.g., Hayes et al. 2006), the overall lack of impact of these compounds on time to metamorphosis suggests a general lack of escape strategy from polluted aquatic environments.

### Abnormality frequency

Exposure to pollutants increased the overall abnormality frequency and there were differences among pollutant types. The exposure to wastewater and nitrogenous pollutants significantly increased the incidence of abnormality frequency, likely due to the alteration of those enzymes involved in development or to DNA damage (Dunson and Connell 1982; Ralph and Petras 1997). In the particular case of the wastewater boron cation, it has been argued that cations may more severely affect the hatching enzyme responsible for enlarging the perivitelline membrane surrounding the embryo than anions (e.g., nitrate) (Dunson and Connell 1982; Laposata and Dunson 1998), thereby increasing the incidence of malformations. Physical abnormalities are correlated with reduced speed and anomalous movements (Laposata and Dunson 1998) that may increase mortality by both predation (e.g., Watkins 1996; Dayton and Fitzgerald 2001) and increased metabolic costs (Rowe et al. 2002). Therefore, the effect of pollutants on abnormality frequency may severely affect individual fitness.

### The effect of multiple stressors on survival: do pollutants act synergistically?

The combination of chemicals such as fertilizers or pesticides with other factors (e.g., predator cues, ultraviolet-B radiation, or even other chemicals) has been reported to have synergistic effects on amphibian survival, growth, and development (e.g., Hatch and Blaustein 2000; Relyea and Mills 2001; Boone et al. 2005; Hayes et al. 2006; Macías et al. 2007; Egea-Serrano et al. 2009b). However, other studies have not found synergistic effects (Boone et al. 2005; Boone and Bridges-Britton 2006). Our factorial analysis across all studies found no overall synergistic effect of pollutants when combined with other stressors. This is important since it implies that while individual chemicals or stressors can interact, there is not an overall pattern that additional stressors consistently increase the lethality of pollutants.

### Conclusions and future research directions

The results of our meta-analysis found that pollution is a major threat to amphibians by having large effects on abnormality frequency and medium effects on survival and mass. Such effects may explain the link between pollutants and population declines described for several areas around the world (Davidson et al. 2001; Sparling et al. 2001; Fellers et al. 2004; Hamer et al. 2004). In contrast, developmental time was not significantly affected by pollutants and the addition of other stressors did not generally exacerbate the effects of the chemicals. To our knowledge, this study is the first quantitative summary analysis to examine the impact of pollutants on amphibians at ecologically relevant concentrations. However, we have to be cautious when interpreting the results of a meta-analysis as evidence for the overall impact of pollution on amphibians in the wild. We stress that more research is needed to achieve more robust conclusions on the traits studied, especially, for development traits and also for factorial designs exhibiting low Rosenberg's fail-safe number. In particular, because pollutants in the environment are expected to be experienced in the presence of other stressors, the study of interactions among factors is of great relevance to fully understand the role of pollutants in amphibian declines (Hayes et al. 2006).

The response variables selected for our meta-analysis are related to amphibian fitness. Nevertheless, they are just a sample of the possible effects that pollutants may have on amphibians. For instance, activity level, habitat use, courtship, and swimming performance are all affected by pollution (e.g., Bridges 1997, 1999; Marco and Blaustein 1999; Shinn et al. 2008; Ortiz-Santaliestra et al. 2009). Therefore, since the ability of individuals to escape from predators (or other stressors such as ultraviolet-B radiation), to feed or even to reproduce, may be affected, it is likely that the impact of pollutants is even higher than that reported in our study and, consequently, such traits should be considered in future meta-analyses.

A final remark is that the response variables were all recorded at the individual level. Nevertheless, they are the expression of the alterations of the physiology of the individuals affected (e.g., Huey and Beitinger 1980a; Widder and Bidwell 2006). Moreover, different species may be differently susceptible to pollution and other stressors, and the interaction among all these factors may lead to impoverished communities dominated by tolerant species (Christensen et al. 2006). All of these considerations underscore the relevance of running meta-analyses at both physiological and community scales to get a broad and comprehensive view of the causes and consequences of chemical pollution on amphibians.

## Supporting Information

**Table S1.** Summary information for each point sample included in the meta-analyses performed for each response variable.
