# Supplementary material for: Understanding of the impact of chemicals on amphibians: a meta-analytic review
Source: Ecol Evol. 2012 Jul;2(7):1382–97. doi: 10.1002/ece3.249 (PMC3434931; doi:10.1002/ece3.249)
Supplement: Supplementary file 1 [file ece30002-1382-SD1.doc]

**Table S1.** Summary information for each point simple included in the meta-analyses performed for each response variable. Hedge´s d+ was calculated after correcting mean and SD by the days each experiment lasted (see Material and Methods section for details). The number of days each experiment lasted is not reported for time to hatching and time to metamorphosis since for these traits time is intrinsically considered.

| Proportion of surviving individuals | | | | | | | | | | | | | | |
| --- | --- | --- | --- | --- | --- | --- | --- | --- | --- | --- | --- | --- | --- | --- |
|  |  |  |  |  |  | n | | Control treatment | | Polluted treatment | |  |  |  |
| Species | Family | Developmental stage | Experimental venue | Pollutant | Days of exposure | Control | Polluted | Mean | SD | Mean | SD | d | Var(d) | Reference |
| *Pseudacris triseriata* | Hylidae | Larvae | Laboratory | Pesticides | 1 | 5 | 5 | 1.00 | 0.00 | 0.45 | 0.27 | -2.62 | 0.74 | Smith, 2001 |
| *Lithobates blairi* | Ranidae | Larvae | Laboratory | Pesticides | 1 | 5 | 5 | 0.95 | 0.11 | 0.00 | 0.00 | -10.85 | 6.29 | Smith, 2001 |
| *Lithobates catesbeianus* | Ranidae | Larvae | Laboratory | Nitrogenous compounds | 15 | 4 | 4 | 1.00 | 0.00 | 0.88 | 0.16 | -0.96 | 0.56 | Smith *et al.*, 2005 |
| *Lithobates clamitans* | Ranidae | Larvae | Laboratory | Nitrogenous compounds | 15 | 4 | 4 | 0.92 | 0.10 | 0.75 | 0.40 | -0.50 | 0.52 | Smith *et al.*, 2005 |
| *Lithobates catesbeianus* | Ranidae | Larvae | Laboratory | Nitrogenous compounds | 15 | 4 | 4 | 0.94 | 0.12 | 0.88 | 0.25 | -0.28 | 0.50 | Smith *et al.*, 2004 |
| *Lithobates catesbeianus* | Ranidae | Larvae | Mesocosms | Nitrogenous compounds | 42 | 3 | 3 | 0.96 | 0.02 | 0.79 | 0.14 | -1.38 | 0.83 | Smith *et al.*, 2006 |
| *Lithobates clamitans* | Ranidae | Larvae | Mesocosms | Nitrogenous compounds | 42 | 3 | 3 | 0.52 | 0.28 | 0.86 | 0.11 | 1.29 | 0.81 | Smith *et al.*, 2006 |
| *Lithobates sylvaticus* | Ranidae | Larvae | Laboratory | Nitrogenous compounds | 4 | 5 | 5 | 0.92 | 0.11 | 0.76 | 0.33 | -0.59 | 0.42 | Smith, 2007 |
| *Lithobates sylvaticus* | Ranidae | Larvae | Laboratory | Phosphorous compounds | 4 | 5 | 5 | 0.92 | 0.11 | 0.84 | 0.22 | -0.42 | 0.41 | Smith, 2007 |
| *Lithobates sylvaticus* | Ranidae | Larvae | Laboratory | Nitrogenous compounds | 4 | 5 | 5 | 0.92 | 0.11 | 0.92 | 0.18 | 0.00 | 0.40 | Smith, 2007 |
| *Lithobates sylvaticus* | Ranidae | Larvae | Laboratory | Nitrogenous compounds | 7 | 5 | 5 | 0.82 | 0.19 | 0.48 | 0.21 | -1.53 | 0.52 | Burgett *et al.*, 2007 |
| *Lithobates sylvaticus* | Ranidae | Larvae | Laboratory | Nitrogenous compounds | 7 | 5 | 5 | 0.89 | 0.14 | 0.31 | 0.14 | -3.75 | 1.10 | Burgett *et al.*, 2007 |
| *Anaxyrus americanus* | Bufonidae | Larvae | Laboratory | Wastewater contaminants | 14 | 5 | 5 | 0.83 | 0.14 | 0.75 | 0.09 | -0.57 | 0.42 | Smith & Burgett, 2005 |
| *Anaxyrus americanus* | Bufonidae | Larvae | Laboratory | Wastewater contaminants | 14 | 5 | 5 | 0.83 | 0.14 | 0.88 | 0.15 | 0.30 | 0.40 | Smith & Burgett, 2005 |
| *Anaxyrus americanus* | Bufonidae | Larvae | Laboratory | Wastewater contaminants | 14 | 5 | 5 | 0.83 | 0.14 | 0.68 | 0.19 | -0.81 | 0.43 | Smith & Burgett, 2005 |
| *Hyla versicolor* | Hylidae | Embryos | Laboratory | Nitrogenous compounds | 15 | 4 | 4 | 0.90 | 0.12 | 1.00 | 0.00 | 1.06 | 0.57 | Vaala *et al.*, 2004 |
| *Lithobates sylvaticus* | Ranidae | Embryos | Laboratory | Nitrogenous compounds | 23 | 5 | 5 | 0.97 | 0.04 | 0.99 | 0.03 | 0.39 | 0.41 | Laposata & Dunson, 1998 |
| *Ambystoma jeffersonianum* | Ambystomatidae | Embryos | Laboratory | Nitrogenous compounds | 25 | 5 | 5 | 0.92 | 0.03 | 0.91 | 0.15 | -0.11 | 0.40 | Laposata & Dunson, 1998 |
| *Anaxyrus americanus* | Bufonidae | Embryos | Laboratory | Nitrogenous compounds | 23 | 5 | 5 | 0.77 | 0.12 | 0.57 | 0.11 | -1.54 | 0.52 | Laposata & Dunson, 1998 |
| *Pleurodeles waltl* | Salamandridae | Embryos | Laboratory | Nitrogenous compounds | 15 | 3 | 3 | 1.00 | 0.00 | 0.94 | 0.05 | -1.32 | 0.81 | Ortiz *et al.*, 2004 |
| *Discoglossus galganoi* | Alytidae | Embryos | Laboratory | Nitrogenous compounds | 15 | 3 | 3 | 0.78 | 0.04 | 0.44 | 0.04 | -7.07 | 4.84 | Ortiz *et al.*, 2004 |
| *Pelobates cultripes* | Pelobatidae | Embryos | Laboratory | Nitrogenous compounds | 15 | 3 | 3 | 0.97 | 0.06 | 0.98 | 0.03 | 0.28 | 0.67 | Ortiz *et al.*, 2004 |
| *Bufo bufo* | Bufonidae | Embryos | Laboratory | Nitrogenous compounds | 15 | 9 | 9 | 0.79 | 0.12 | 0.61 | 0.11 | -1.50 | 0.28 | Ortiz *et al.*, 2004 |
| *Epidalea calamita* | Bufonidae | Embryos | Laboratory | Nitrogenous compounds | 15 | 9 | 9 | 0.92 | 0.07 | 0.91 | 0.08 | -0.20 | 0.22 | Ortiz *et al.*, 2004 |
| *Hyla arborea* | Hylidae | Embryos | Laboratory | Nitrogenous compounds | 15 | 3 | 3 | 0.95 | 0.05 | 0.03 | 0.06 | -13.55 | 15.96 | Ortiz *et al.*, 2004 |
| *Lithobates sphenocephalus* | Ranidae | Larvae | Laboratory | Nitrogenous compounds | 105 | 3 | 3 | 0.83 | 0.19 | 0.55 | 0.21 | -1.12 | 0.77 | Ortiz-Santaliestra & Sparling, 2007 |
| *Lithobates sphenocephalus* | Ranidae | Larvae | Laboratory | Wastewater contaminants | 105 | 3 | 3 | 0.83 | 0.19 | 0.55 | 0.28 | -0.94 | 0.74 | Ortiz-Santaliestra & Sparling, 2007 |
| *Triturus pygmaeus* | Salamandridae | Embryos | Laboratory | Nitrogenous compounds | 25 | 4 | 4 | 0.35 | 0.03 | 0.44 | 0.03 | 2.61 | 0.93 | Ortiz-Santaliestra *et al.*, 2007 |
| *Hyla versicolor* | Hylidae | Larvae | Mesocosms | Pesticides | 23 | 5 | 5 | 0.71 | 0.20 | 0.57 | 0.30 | -0.52 | 0.41 | Relyea *et al.*, 2005 |
| *Hyla versicolor* | Hylidae | Larvae | Mesocosms | Pesticides | 23 | 5 | 5 | 0.71 | 0.20 | 0.53 | 0.10 | -1.02 | 0.45 | Relyea *et al.*, 2005 |
| *Anaxyrus americanus* | Bufonidae | Larvae | Mesocosms | Pesticides | 23 | 5 | 5 | 0.94 | 0.10 | 0.83 | 0.10 | -1.01 | 0.45 | Relyea *et al.*, 2005 |
| *Anaxyrus americanus* | Bufonidae | Larvae | Mesocosms | Pesticides | 23 | 5 | 5 | 0.94 | 0.10 | 0.23 | 0.10 | -6.47 | 2.49 | Relyea *et al.*, 2005 |
| *Lithobates pipiens* | Ranidae | Larvae | Mesocosms | Pesticides | 23 | 5 | 5 | 0.99 | 0.05 | 0.97 | 0.05 | -0.41 | 0.41 | Relyea *et al.*, 2005 |
| *Lithobates pipiens* | Ranidae | Larvae | Mesocosms | Pesticides | 23 | 5 | 5 | 0.99 | 0.05 | 0.69 | 0.20 | -1.87 | 0.58 | Relyea *et al.*, 2005 |
| *Ambystoma maculatum* | Ambystomatidae | Larvae | Mesocosms | Pesticides | 137 | 4 | 4 | 0.84 | 0.13 | 0.80 | 0.26 | -0.18 | 0.50 | Metts *et al.*, 2005 |
| *Ambystoma opacum* | Ambystomatidae | Larvae | Mesocosms | Pesticides | 137 | 4 | 4 | 0.96 | 0.09 | 0.21 | 0.03 | -10.10 | 6.88 | Metts *et al.*, 2005 |
| *Xenopus laevis* | Pipidae | Larvae | Laboratory | Pesticides | 78 | 11 | 11 | 0.95 | 0.03 | 0.95 | 0.03 | -0.12 | 0.18 | Carr *et al.*, 2003 |
| *Rana arvalis* | Ranidae | Embryos | Laboratory | Pesticides | 2 | 3 | 3 | 0.98 | 0.03 | 0.97 | 0.04 | -0.15 | 0.67 | Greulich & Pflumacher, 2003 |
| *Lithobates catesbeianus* | Ranidae | Larvae | Mesocosms | Pesticides | 31 | 4 | 4 | 0.68 | 0.08 | 0.68 | 0.08 | -0.06 | 0.50 | Relyea, 2006 |
| *Lithobates clamitans* | Ranidae | Larvae | Mesocosms | Pesticides | 31 | 4 | 4 | 0.61 | 0.27 | 0.64 | 0.26 | 0.09 | 0.50 | Relyea, 2006 |
| *Hyla versicolor* | Hylidae | Larvae | Laboratory | Pesticides | 10 | 4 | 4 | 0.91 | 0.09 | 0.40 | 0.31 | -1.91 | 0.73 | Relyea & Mills, 2001 |
| *Hyla versicolor* | Hylidae | Larvae | Laboratory | Pesticides | 16 | 4 | 4 | 0.96 | 0.04 | 1.00 | 0.00 | 1.38 | 0.62 | Relyea & Mills, 2001 |
| *Hyla versicolor* | Hylidae | Larvae | Mesocosms | Pesticides | 20 | 5 | 5 | 0.66 | 0.15 | 0.01 | 0.04 | -5.19 | 1.75 | Relyea, 2005 |
| *Lithobates pipiens* | Ranidae | Larvae | Mesocosms | Pesticides | 20 | 5 | 5 | 0.98 | 0.04 | 0.09 | 0.08 | -13.29 | 9.23 | Relyea, 2005 |
| *Lithobates sylvaticus* | Ranidae | Metamorphs | Laboratory | Pesticides | 1 | 4 | 4 | 0.95 | 0.09 | 0.31 | 0.14 | -4.63 | 1.84 | Relyea, 2005 |
| *Lithobates sphenocephalus* | Ranidae | Larvae | Mesocosms | Pesticides | 12 | 4 | 4 | 0.92 | 0.04 | 0.91 | 0.04 | -0.22 | 0.50 | Widder & Bidwell, 2006 |
| *Lithobates catesbeianus* | Ranidae | Larvae | Mesocosms | Pesticides | 69 | 3 | 3 | 0.64 | 0.21 | 0.91 | 0.21 | 1.04 | 0.76 | Boone & Semlitsch, 2003 |
| *Ambystoma maculatum* | Ambystomatidae | Larvae | Mesocosms | Pesticides | 14 | 6 | 6 | 0.37 | 0.32 | 0.73 | 0.27 | 1.13 | 0.39 | Relyea, 2005 |
| *Lithobates pipiens* | Ranidae | Larvae | Mesocosms | Pesticides | 14 | 6 | 6 | 0.28 | 0.15 | 0.59 | 0.27 | 1.26 | 0.40 | Relyea, 2005 |
| *Hyla versicolor* | Hylidae | Larvae | Mesocosms | Pesticides | 14 | 6 | 6 | 0.40 | 0.36 | 0.27 | 0.23 | -0.40 | 0.34 | Relyea, 2005 |
| *Lithobates sylvaticus* | Ranidae | Larvae | Mesocosms | Pesticides | 14 | 6 | 6 | 0.50 | 0.30 | 0.72 | 0.34 | 0.63 | 0.35 | Relyea, 2005 |
| *Anaxyrus americanus* | Bufonidae | Larvae | Mesocosms | Pesticides | 14 | 6 | 6 | 0.15 | 0.30 | 0.09 | 0.15 | -0.24 | 0.34 | Relyea, 2005 |
| *Pseudacris crucifer* | Hylidae | Larvae | Mesocosms | Pesticides | 14 | 6 | 6 | 0.70 | 0.19 | 0.72 | 0.11 | 0.10 | 0.33 | Relyea, 2005 |
| *Ambystoma barbouri* | Ambystomatidae | Embryos | Laboratory | Pesticides | 37 | 4 | 4 | 1.00 | 0.00 | 0.85 | 0.12 | -1.49 | 0.64 | Rohr *et al.*, 2003 |
| *Ambystoma barbouri* | Ambystomatidae | Embryos | Laboratory | Pesticides | 37 | 4 | 4 | 1.00 | 0.00 | 0.92 | 0.06 | -1.60 | 0.66 | Rohr *et al.*, 2003 |
| *Ambystoma barbouri* | Ambystomatidae | Embryos | Laboratory | Pesticides | 37 | 4 | 4 | 1.00 | 0.00 | 0.94 | 0.08 | -0.90 | 0.55 | Rohr *et al.*, 2003 |
| *Ambystoma barbouri* | Ambystomatidae | Embryos | Laboratory | Pesticides | 37 | 4 | 4 | 1.00 | 0.00 | 0.79 | 0.25 | -1.04 | 0.57 | Rohr *et al.*, 2003 |
| *Rana cascadae* | Ranidae | Larvae | Laboratory | Nitrogenous compounds | 21 | 4 | 4 | 0.99 | 0.00 | 0.94 | 0.10 | -0.61 | 0.52 | Hatch & Blaustein, 2000 |
| *Rana cascadae* | Ranidae | Larvae | Laboratory | Nitrogenous compounds | 21 | 4 | 4 | 0.94 | 0.18 | 1.00 | 0.00 | 0.41 | 0.51 | Hatch & Blaustein, 2000 |
| *Lithobates catesbeianus* | Ranidae | Larvae | Laboratory | Nitrogenous compounds | 15 | 4 | 4 | 1.00 | 0.00 | 0.96 | 0.08 | -0.62 | 0.52 | Smith *et al.*, 2005 |
| *Lithobates catesbeianus* | Ranidae | Larvae | Laboratory | Nitrogenous compounds | 15 | 4 | 4 | 1.00 | 0.00 | 0.79 | 0.25 | -1.02 | 0.57 | Smith *et al.*, 2005 |
| *Lithobates clamitans* | Ranidae | Larvae | Laboratory | Nitrogenous compounds | 15 | 4 | 4 | 0.92 | 0.10 | 0.71 | 0.48 | -0.53 | 0.52 | Smith *et al.*, 2005 |
| *Lithobates clamitans* | Ranidae | Larvae | Laboratory | Nitrogenous compounds | 15 | 4 | 4 | 0.92 | 0.10 | 0.50 | 0.58 | -0.88 | 0.55 | Smith *et al.*, 2005 |
| *Lithobates clamitans* | Ranidae | Larvae | Laboratory | Nitrogenous compounds | 15 | 4 | 4 | 0.92 | 0.10 | 0.71 | 0.25 | -0.96 | 0.56 | Smith *et al.*, 2005 |
| *Lithobates clamitans* | Ranidae | Larvae | Laboratory | Nitrogenous compounds | 15 | 4 | 4 | 0.92 | 0.10 | 0.08 | 0.17 | -5.32 | 2.27 | Smith *et al.*, 2005 |
| *Lithobates catesbeianus* | Ranidae | Larvae | Laboratory | Nitrogenous compounds | 15 | 4 | 4 | 0.94 | 0.12 | 0.81 | 0.12 | -0.88 | 0.55 | Smith *et al.*, 2004 |
| *Lithobates catesbeianus* | Ranidae | Larvae | Laboratory | Nitrogenous compounds | 15 | 4 | 4 | 0.94 | 0.12 | 0.88 | 0.14 | -0.41 | 0.51 | Smith *et al.*, 2004 |
| *Lithobates catesbeianus* | Ranidae | Larvae | Laboratory | Nitrogenous compounds | 15 | 4 | 4 | 0.94 | 0.12 | 1.00 | 0.00 | 0.61 | 0.52 | Smith *et al.*, 2004 |
| *Lithobates catesbeianus* | Ranidae | Larvae | Laboratory | Nitrogenous compounds | 15 | 4 | 4 | 0.94 | 0.12 | 1.00 | 0.00 | 0.61 | 0.52 | Smith *et al.*, 2004 |
| *Lithobates sylvaticus* | Ranidae | Larvae | Laboratory | Nitrogenous compounds | 4 | 5 | 5 | 0.92 | 0.11 | 0.72 | 0.11 | -1.65 | 0.54 | Smith, 2007 |
| *Lithobates sylvaticus* | Ranidae | Larvae | Laboratory | Nitrogenous compounds | 4 | 5 | 5 | 0.92 | 0.11 | 0.72 | 0.23 | -1.01 | 0.45 | Smith, 2007 |
| *Lithobates sylvaticus* | Ranidae | Larvae | Laboratory | Nitrogenous compounds | 4 | 5 | 5 | 0.92 | 0.11 | 0.96 | 0.09 | 0.36 | 0.41 | Smith, 2007 |
| *Lithobates sylvaticus* | Ranidae | Larvae | Laboratory | Nitrogenous compounds | 4 | 5 | 5 | 0.92 | 0.11 | 0.84 | 0.26 | -0.36 | 0.41 | Smith, 2007 |
| *Lithobates sylvaticus* | Ranidae | Larvae | Laboratory | Phosphorous compounds | 4 | 5 | 5 | 0.92 | 0.11 | 0.96 | 0.09 | 0.36 | 0.41 | Smith, 2007 |
| *Lithobates sylvaticus* | Ranidae | Larvae | Laboratory | Phosphorous compounds | 4 | 5 | 5 | 0.92 | 0.11 | 0.96 | 0.09 | 0.36 | 0.41 | Smith, 2007 |
| *Lithobates sylvaticus* | Ranidae | Larvae | Laboratory | Phosphorous compounds | 4 | 5 | 5 | 0.92 | 0.11 | 0.88 | 0.11 | -0.33 | 0.41 | Smith, 2007 |
| *Lithobates sylvaticus* | Ranidae | Larvae | Laboratory | Phosphorous compounds | 4 | 5 | 5 | 0.92 | 0.11 | 0.89 | 0.09 | -0.27 | 0.40 | Smith, 2007 |
| *Lithobates sylvaticus* | Ranidae | Larvae | Laboratory | Nitrogenous compounds | 4 | 5 | 5 | 0.92 | 0.11 | 0.84 | 0.17 | -0.51 | 0.41 | Smith, 2007 |
| *Lithobates sylvaticus* | Ranidae | Larvae | Laboratory | Nitrogenous compounds | 4 | 5 | 5 | 0.92 | 0.11 | 0.80 | 0.20 | -0.67 | 0.42 | Smith, 2007 |
| *Lithobates sylvaticus* | Ranidae | Larvae | Laboratory | Nitrogenous compounds | 4 | 5 | 5 | 0.92 | 0.11 | 0.92 | 0.11 | 0.00 | 0.40 | Smith, 2007 |
| *Lithobates sylvaticus* | Ranidae | Larvae | Laboratory | Nitrogenous compounds | 4 | 5 | 5 | 0.92 | 0.11 | 0.92 | 0.18 | 0.00 | 0.40 | Smith, 2007 |
| *Lithobates sylvaticus* | Ranidae | Larvae | Laboratory | Nitrogenous compounds | 7 | 5 | 5 | 0.82 | 1.88 | 0.68 | 0.12 | -0.10 | 0.40 | Burgett *et al.*, 2007 |
| *Lithobates sylvaticus* | Ranidae | Larvae | Laboratory | Nitrogenous compounds | 7 | 5 | 5 | 0.89 | 0.14 | 0.48 | 0.31 | -1.53 | 0.52 | Burgett *et al.*, 2007 |
| *Lithobates sylvaticus* | Ranidae | Larvae | Laboratory | Nitrogenous compounds | 7 | 5 | 5 | 0.89 | 0.14 | 0.34 | 0.17 | -3.20 | 0.91 | Burgett *et al.*, 2007 |
| *Xenopus laevis* | Pipidae | Larvae | Laboratory | Wastewater contaminants | 11 | 5 | 5 | 0.90 | 0.13 | 0.30 | 0.40 | -1.81 | 0.56 | Fraker & Smith, 2005 |
| *Xenopus laevis* | Pipidae | Larvae | Laboratory | Wastewater contaminants | 11 | 5 | 5 | 0.90 | 0.13 | 0.60 | 0.54 | -0.69 | 0.42 | Fraker & Smith, 2005 |
| *Xenopus laevis* | Pipidae | Larvae | Laboratory | Wastewater contaminants | 11 | 4 | 4 | 0.88 | 0.14 | 0.88 | 0.24 | 0.00 | 0.50 | Fraker & Smith, 2005 |
| *Xenopus laevis* | Pipidae | Larvae | Laboratory | Wastewater contaminants | 11 | 4 | 4 | 0.88 | 0.14 | 0.94 | 0.12 | 0.40 | 0.51 | Fraker & Smith, 2005 |
| *Anaxyrus americanus* | Bufonidae | Larvae | Laboratory | Wastewater contaminants | 14 | 5 | 5 | 0.83 | 0.14 | 0.75 | 0.18 | -0.42 | 0.41 | Smith & Burgett, 2005 |
| *Anaxyrus americanus* | Bufonidae | Larvae | Laboratory | Wastewater contaminants | 14 | 5 | 5 | 0.83 | 0.14 | 0.90 | 0.11 | 0.54 | 0.41 | Smith & Burgett, 2005 |
| *Anaxyrus americanus* | Bufonidae | Larvae | Laboratory | Wastewater contaminants | 14 | 5 | 5 | 0.83 | 0.14 | 0.88 | 0.09 | 0.38 | 0.41 | Smith & Burgett, 2005 |
| *Hyla versicolor* | Hylidae | Larvae | Laboratory | Nitrogenous compounds | 15 | 4 | 4 | 0.90 | 0.12 | 0.95 | 0.10 | 0.40 | 0.51 | Vaala *et al.*, 2004 |
| *Hyla versicolor* | Hylidae | Larvae | Laboratory | Nitrogenous compounds | 15 | 4 | 4 | 0.90 | 0.12 | 0.95 | 0.10 | 0.40 | 0.51 | Vaala *et al.*, 2004 |
| *Hyla versicolor* | Hylidae | Larvae | Laboratory | Nitrogenous compounds | 15 | 4 | 4 | 0.90 | 0.12 | 1.00 | 0.00 | 1.06 | 0.57 | Vaala *et al.*, 2004 |
| *Hyla versicolor* | Hylidae | Larvae | Laboratory | Nitrogenous compounds | 15 | 4 | 4 | 0.90 | 0.12 | 0.95 | 0.10 | 0.40 | 0.51 | Vaala *et al.*, 2004 |
| *Lithobates sylvaticus* | Ranidae | Embryos | Laboratory | Wastewater contaminants | 23 | 5 | 5 | 0.97 | 0.04 | 0.97 | 0.04 | 0.00 | 0.40 | Laposata & Dunson, 1998 |
| *Ambystoma jeffersonianum* | Ambystomatidae | Embryos | Laboratory | Wastewater contaminants | 25 | 5 | 5 | 0.97 | 0.06 | 0.99 | 0.03 | 0.27 | 0.40 | Laposata & Dunson, 1998 |
| *Ambystoma maculatum* | Ambystomatidae | Embryos | Laboratory | Wastewater contaminants | 44 | 5 | 5 | 0.89 | 0.17 | 0.80 | 0.14 | -0.54 | 0.41 | Laposata & Dunson, 1998 |
| *Anaxyrus americanus* | Bufonidae | Embryos | Laboratory | Wastewater contaminants | 23 | 5 | 5 | 0.82 | 0.07 | 0.11 | 0.06 | -9.82 | 5.23 | Laposata & Dunson, 1998 |
| *Lithobates sylvaticus* | Ranidae | Embryos | Laboratory | Nitrogenous compounds | 23 | 5 | 5 | 0.97 | 0.04 | 1.00 | 0.00 | 0.96 | 0.45 | Laposata & Dunson, 1998 |
| *Ambystoma jeffersonianum* | Ambystomatidae | Embryos | Laboratory | Nitrogenous compounds | 25 | 5 | 5 | 0.92 | 0.03 | 0.96 | 0.04 | 1.11 | 0.46 | Laposata & Dunson, 1998 |
| *Ambystoma maculatum* | Ambystomatidae | Embryos | Laboratory | Nitrogenous compounds | 44 | 5 | 5 | 0.87 | 0.00 | 0.87 | 0.08 | 0.00 | 0.40 | Laposata & Dunson, 1998 |
| *Anaxyrus americanus* | Bufonidae | Embryos | Laboratory | Nitrogenous compounds | 23 | 5 | 5 | 0.77 | 0.12 | 0.72 | 0.10 | -0.39 | 0.41 | Laposata & Dunson, 1998 |
| *Lithobates sylvaticus* | Ranidae | Embryos | Laboratory | Nitrogenous compounds | 23 | 5 | 5 | 0.97 | 0.04 | 0.99 | 0.03 | 0.39 | 0.41 | Laposata & Dunson, 1998 |
| *Ambystoma jeffersonianum* | Ambystomatidae | Embryos | Laboratory | Nitrogenous compounds | 25 | 5 | 5 | 0.92 | 0.03 | 0.97 | 0.04 | 1.47 | 0.51 | Laposata & Dunson, 1998 |
| *Ambystoma maculatum* | Ambystomatidae | Embryos | Laboratory | Nitrogenous compounds | 44 | 5 | 5 | 0.87 | 0.00 | 0.87 | 0.13 | 0.00 | 0.40 | Laposata & Dunson, 1998 |
| *Anaxyrus americanus* | Bufonidae | Embryos | Laboratory | Nitrogenous compounds | 23 | 5 | 5 | 0.76 | 0.12 | 0.67 | 0.10 | -0.80 | 0.43 | Laposata & Dunson, 1998 |
| *Pleurodeles waltl* | Salamandridae | Embryos | Laboratory | Nitrogenous compounds | 15 | 3 | 3 | 1.00 | 0.00 | 0.97 | 0.05 | -0.66 | 0.70 | Ortiz *et al.*, 2004 |
| *Discoglossus galganoi* | Alytidae | Embryos | Laboratory | Nitrogenous compounds | 15 | 3 | 3 | 0.98 | 0.04 | 0.96 | 0.04 | -0.46 | 0.68 | Ortiz *et al.*, 2004 |
| *Pelobates cultripes* | Pelobatidae | Embryos | Laboratory | Nitrogenous compounds | 15 | 3 | 3 | 0.97 | 0.58 | 0.98 | 0.03 | 0.03 | 0.67 | Ortiz *et al.*, 2004 |
| *Bufo bufo* | Bufonidae | Embryos | Laboratory | Nitrogenous compounds | 15 | 9 | 9 | 0.79 | 0.12 | 0.80 | 0.06 | 0.06 | 0.22 | Ortiz *et al.*, 2004 |
| *Epidalea calamita* | Bufonidae | Embryos | Laboratory | Nitrogenous compounds | 15 | 9 | 9 | 0.92 | 0.07 | 0.88 | 0.06 | -0.56 | 0.23 | Ortiz *et al.*, 2004 |
| *Hyla arborea* | Hylidae | Embryos | Laboratory | Nitrogenous compounds | 15 | 3 | 3 | 0.95 | 0.05 | 0.18 | 0.08 | -9.54 | 8.25 | Ortiz *et al.*, 2004 |
| *Lithobates sphenocephalus* | Ranidae | Larvae | Laboratory | Wastewater contaminants | 105 | 3 | 3 | 0.83 | 0.19 | 0.15 | 0.12 | -3.41 | 1.63 | Ortiz-Santaliestra & Sparling, 2007 |
| *Ambystoma maculatum* | Ambystomatidae | Larvae | Mesocosms | Pesticides | 137 | 4 | 4 | 0.84 | 0.13 | 0.04 | 0.09 | -6.42 | 3.08 | Metts *et al.*, 2005 |
| *Xenopus laevis* | Pipidae | Larvae | Laboratory | Pesticides | 78 | 11 | 11 | 0.95 | 0.03 | 0.94 | 0.04 | -0.35 | 0.18 | Carr *et al.*, 2003 |
| *Xenopus laevis* | Pipidae | Larvae | Laboratory | Pesticides | 78 | 11 | 11 | 0.95 | 0.03 | 0.96 | 0.03 | 0.34 | 0.18 | Carr *et al.*, 2003 |
| *Rana arvalis* | Ranidae | Larvae | Laboratory | Pesticides | 2 | 3 | 3 | 0.98 | 0.03 | 0.77 | 0.03 | -4.80 | 2.59 | Greulich & Pfluggmacher, 2003 |
| *Lithobates catesbeianus* | Ranidae | Larvae | Mesocosms | Pesticides | 31 | 4 | 4 | 0.68 | 0.08 | 0.58 | 0.20 | -0.60 | 0.52 | Relyea, 2006 |
| *Lithobates clamitans* | Ranidae | Larvae | Mesocosms | Pesticides | 31 | 4 | 4 | 0.61 | 0.27 | 0.74 | 0.12 | 0.54 | 0.52 | Relyea, 2006 |
| *Hyla versicolor* | Hylidae | Larvae | Laboratory | Pesticides | 16 | 4 | 4 | 0.96 | 0.04 | 0.78 | 0.24 | -0.91 | 0.55 | Relyea & Mills, 2001 |
| *Hyla versicolor* | Hylidae | Larvae | Laboratory | Pesticides | 16 | 4 | 4 | 0.96 | 0.04 | 0.68 | 0.43 | -0.80 | 0.54 | Relyea & Mills, 2001 |
| *Hyla versicolor* | Hylidae | Larvae | Laboratory | Pesticides | 16 | 4 | 4 | 0.96 | 0.04 | 0.91 | 0.18 | -0.29 | 0.51 | Relyea & Mills, 2001 |
| *Ambystoma maculatum* | Ambystomatidae | Larvae | Mesocosms | Pesticides | 14 | 6 | 6 | 0.37 | 0.32 | 0.31 | 0.42 | -0.14 | 0.33 | Relyea, 2005 |
| *Ambystoma maculatum* | Ambystomatidae | Larvae | Mesocosms | Pesticides | 14 | 6 | 6 | 0.37 | 0.32 | 0.91 | 0.20 | 1.86 | 0.48 | Relyea, 2005 |
| *Ambystoma maculatum* | Ambystomatidae | Larvae | Mesocosms | Pesticides | 14 | 6 | 6 | 0.37 | 0.32 | 0.56 | 0.36 | 0.51 | 0.34 | Relyea, 2005 |
| *Lithobates pipiens* | Ranidae | Larvae | Mesocosms | Pesticides | 14 | 6 | 6 | 0.28 | 0.15 | 0.40 | 0.28 | 0.49 | 0.34 | Relyea, 2005 |
| *Lithobates pipiens* | Ranidae | Larvae | Mesocosms | Pesticides | 14 | 6 | 6 | 0.28 | 0.15 | 0.30 | 0.18 | 0.08 | 0.33 | Relyea, 2005 |
| *Hyla versicolor* | Hylidae | Larvae | Mesocosms | Pesticides | 14 | 6 | 6 | 0.40 | 0.36 | 0.37 | 0.32 | -0.07 | 0.33 | Relyea, 2005 |
| *Hyla versicolor* | Hylidae | Larvae | Mesocosms | Pesticides | 14 | 6 | 6 | 0.40 | 0.36 | 0.26 | 0.22 | -0.43 | 0.34 | Relyea, 2005 |
| *Lithobates sylvaticus* | Ranidae | Larvae | Mesocosms | Pesticides | 14 | 6 | 6 | 0.50 | 0.30 | 0.63 | 0.24 | 0.44 | 0.34 | Relyea, 2005 |
| *Lithobates sylvaticus* | Ranidae | Larvae | Mesocosms | Pesticides | 14 | 6 | 6 | 0.50 | 0.30 | 0.56 | 0.18 | 0.21 | 0.34 | Relyea, 2005 |
| *Anaxyrus americanus* | Bufonidae | Larvae | Mesocosms | Pesticides | 14 | 6 | 6 | 0.15 | 0.30 | 0.08 | 0.16 | -0.26 | 0.34 | Relyea, 2005 |
| *Anaxyrus americanus* | Bufonidae | Larvae | Mesocosms | Pesticides | 14 | 6 | 6 | 0.15 | 0.30 | 0.15 | 0.06 | 0.03 | 0.33 | Relyea, 2005 |
| *Anaxyrus americanus* | Bufonidae | Larvae | Mesocosms | Pesticides | 14 | 6 | 6 | 0.15 | 0.30 | 0.02 | 0.06 | -0.53 | 0.35 | Relyea, 2005 |
| *Pseudacris crucifer* | Hylidae | Larvae | Mesocosms | Pesticides | 14 | 6 | 6 | 0.70 | 0.19 | 0.71 | 0.20 | 0.05 | 0.33 | Relyea, 2005 |
| *Pseudacris crucifer* | Hylidae | Larvae | Mesocosms | Pesticides | 14 | 6 | 6 | 0.70 | 0.19 | 0.72 | 0.14 | 0.11 | 0.33 | Relyea, 2005 |
| *Pseudacris crucifer* | Hylidae | Larvae | Mesocosms | Pesticides | 14 | 6 | 6 | 0.70 | 0.19 | 0.45 | 0.26 | -1.01 | 0.38 | Relyea, 2005 |
| *Rana cascadae* | Ranidae | Larvae | Laboratory | Nitrogenous compounds | 21 | 4 | 4 | 0.99 | 0.00 | 0.90 | 0.12 | -0.92 | 0.55 | Hatch & Blaustein, 2000 |
| *Rana cascadae* | Ranidae | Larvae | Laboratory | Nitrogenous compounds | 21 | 4 | 4 | 0.94 | 0.18 | 1.00 | 0.00 | 0.41 | 0.51 | Hatch & Blaustein, 2000 |
| *Ambystoma barbouri* | Ambystomatidae | Embryos | Laboratory | Pesticides | 37 | 4 | 4 | 1.00 | 0.00 | 0.90 | 0.08 | -1.47 | 0.63 | Rohr *et al.*, 2003 |
| *Ambystoma barbouri* | Ambystomatidae | Embryos | Laboratory | Pesticides | 37 | 4 | 4 | 1.00 | 0.00 | 0.83 | 0.13 | -1.55 | 0.65 | Rohr *et al.*, 2003 |
| *Ambystoma barbouri* | Ambystomatidae | Embryos | Laboratory | Pesticides | 37 | 4 | 4 | 1.00 | 0.00 | 0.84 | 0.16 | -1.23 | 0.59 | Rohr *et al.*, 2003 |
| *Ambystoma barbouri* | Ambystomatidae | Embryos | Laboratory | Pesticides | 37 | 4 | 4 | 1.00 | 0.00 | 0.83 | 0.14 | -1.42 | 0.63 | Rohr *et al.*, 2003 |
| *Ambystoma maculatum* | Ambystomatidae | Embryos | Enclosures | Road de-icers | 18 | 17 | 17 | 0.84 | 0.03 | 0.68 | 0.09 | -2.33 | 0.20 | Karraker *et al.*, 2008 |
| *Ambystoma maculatum* | Ambystomatidae | Embryos | Enclosures | Road de-icers | 18 | 17 | 17 | 0.84 | 0.03 | 0.03 | 0.02 | -31.02 | 14.27 | Karraker *et al.*, 2008 |
| *Ambystoma maculatum* | Ambystomatidae | Larvae | Enclosures | Road de-icers | 70 | 5 | 5 | 0.59 | 0.05 | 0.38 | 0.09 | -2.61 | 0.74 | Karraker *et al.*, 2008 |
| *Ambystoma maculatum* | Ambystomatidae | Larvae | Enclosures | Road de-icers | 70 | 5 | 5 | 0.59 | 0.05 | 0.11 | 0.03 | -10.52 | 5.93 | Karraker *et al.*, 2008 |
| *Lithobates sylvaticus* | Ranidae | Embryos | Enclosures | Road de-icers | 18 | 17 | 17 | 0.91 | 0.02 | 0.77 | 0.04 | -4.32 | 0.39 | Karraker *et al.*, 2008 |
| *Lithobates sylvaticus* | Ranidae | Embryos | Enclosures | Road de-icers | 18 | 17 | 17 | 0.91 | 0.02 | 0.41 | 0.07 | -9.48 | 1.44 | Karraker *et al.*, 2008 |
| *Lithobates sylvaticus* | Ranidae | Larvae | Enclosures | Road de-icers | 70 | 5 | 5 | 0.64 | 0.02 | 0.54 | 0.13 | -0.97 | 0.45 | Karraker *et al.*, 2008 |
| *Lithobates sylvaticus* | Ranidae | Larvae | Enclosures | Road de-icers | 70 | 5 | 5 | 0.64 | 0.02 | 0.20 | 0.09 | -6.10 | 2.26 | Karraker *et al.*, 2008 |
| *Pseudacris regilla* | Hylidae | Larvae | Laboratory | Nitrogenous compounds | 7 | 5 | 5 | 0.96 | 0.05 | 0.97 | 0.05 | 0.15 | 0.40 | Romansic *et al*., 2006 |
| *Pseudacris regilla* | Hylidae | Larvae | Laboratory | Nitrogenous compounds | 7 | 5 | 5 | 0.96 | 0.05 | 1.00 | 0.00 | 1.00 | 0.45 | Romansic *et al.*, 2006 |
| *Rana aurora* | Ranidae | Larvae | Laboratory | Nitrogenous compounds | 7 | 5 | 5 | 0.92 | 0.07 | 0.78 | 0.07 | -1.76 | 0.56 | Romansic *et al.*, 2006 |
| *Rana aurora* | Ranidae | Larvae | Laboratory | Nitrogenous compounds | 7 | 5 | 5 | 0.92 | 0.07 | 0.79 | 0.08 | -1.65 | 0.54 | Romansic *et al.*, 2006 |
| *Discoglossus galganoi* | Alytidae | Embryos | Laboratory | Nitrogenous compounds | 15 | 3 | 3 | 0.98 | 0.04 | 0.96 | 0.04 | -0.46 | 0.68 | Ortiz-Santaliestra *et al.*, 2006 |
| *Discoglossus galganoi* | Alytidae | Embryos | Laboratory | Nitrogenous compounds | 15 | 3 | 3 | 0.98 | 0.04 | 0.98 | 0.04 | 0.00 | 0.67 | Ortiz-Santaliestra *et al.*, 2006 |
| *Discoglossus galganoi* | Alytidae | Embryos | Laboratory | Nitrogenous compounds | 15 | 3 | 3 | 1.00 | 0.00 | 0.96 | 0.08 | -0.65 | 0.70 | Ortiz-Santaliestra *et al.*, 2006 |
| *Discoglossus galganoi* | Alytidae | Embryos | Laboratory | Nitrogenous compounds | 15 | 3 | 3 | 0.98 | 0.04 | 0.44 | 0.04 | -11.24 | 11.20 | Ortiz-Santaliestra *et al.*, 2006 |
| *Discoglossus galganoi* | Alytidae | Embryos | Laboratory | Nitrogenous compounds | 15 | 3 | 3 | 1.00 | 0.00 | 0.93 | 0.07 | -1.13 | 0.77 | Ortiz-Santaliestra *et al.*, 2006 |
| *Hyla meridionalis* | Hylidae | Embryos | Laboratory | Nitrogenous compounds | 15 | 3 | 3 | 0.95 | 0.06 | 0.95 | 0.04 | 0.15 | 0.67 | Ortiz-Santaliestra *et al.*, 2006 |
| *Hyla meridionalis* | Hylidae | Embryos | Laboratory | Nitrogenous compounds | 15 | 3 | 3 | 1.00 | 0.00 | 0.69 | 0.49 | -0.73 | 0.71 | Ortiz-Santaliestra *et al.*, 2006 |
| *Hyla meridionalis* | Hylidae | Embryos | Laboratory | Nitrogenous compounds | 15 | 3 | 3 | 0.95 | 0.06 | 1.00 | 0.00 | 1.11 | 0.77 | Ortiz-Santaliestra *et al.*, 2006 |
| *Hyla meridionalis* | Hylidae | Embryos | Laboratory | Nitrogenous compounds | 15 | 3 | 3 | 1.00 | 0.00 | 0.98 | 0.04 | -0.66 | 0.70 | Ortiz-Santaliestra *et al.*, 2006 |
| *Hyla meridionalis* | Hylidae | Embryos | Laboratory | Nitrogenous compounds | 15 | 3 | 3 | 0.95 | 0.06 | 1.00 | 0.00 | 1.11 | 0.77 | Ortiz-Santaliestra *et al.*, 2006 |
| *Hyla meridionalis* | Hylidae | Embryos | Laboratory | Nitrogenous compounds | 15 | 3 | 3 | 1.00 | 0.00 | 0.56 | 0.50 | -1.00 | 0.75 | Ortiz-Santaliestra *et al.*, 2006 |
| *Epidalea calamita* | Bufonidae | Embryos | Laboratory | Nitrogenous compounds | 12 | 3 | 3 | 1.00 | 0.00 | 0.98 | 0.03 | -0.66 | 0.70 | Ortiz-Santaliestra *et al.*, 2006 |
| *Pelobates cultripes* | Pelobatidae | Embryos | Laboratory | Nitrogenous compounds | 15 | 3 | 3 | 0.97 | 0.06 | 0.98 | 0.03 | 0.28 | 0.67 | Ortiz *et al.*, 2004 |
| *Discoglossus galganoi* | Alytidae | Embryos | Laboratory | Nitrogenous compounds | 15 | 3 | 3 | 0.98 | 0.04 | 0.98 | 0.04 | 0.00 | 0.67 | Ortiz *et al.*, 2004 |
| *Hyla arborea* | Hylidae | Embryos | Laboratory | Nitrogenous compounds | 15 | 3 | 3 | 0.95 | 0.05 | 0.07 | 0.06 | -13.05 | 14.85 | Ortiz *et al.*, 2004 |
| *Lithobates clamitans* | Ranidae | Larvae | Laboratory | Road de-icers | 7 | 5 | 5 | 0.64 | 0.39 | 0.84 | 0.09 | 0.65 | 0.42 | Dougherty & Smith, 2006 |
| *Lithobates clamitans* | Ranidae | Larvae | Laboratory | Road de-icers | 7 | 5 | 5 | 0.64 | 0.39 | 0.68 | 0.27 | 0.11 | 0.40 | Dougherty & Smith, 2006 |
| *Lithobates clamitans* | Ranidae | Larvae | Laboratory | Road de-icers | 7 | 5 | 5 | 0.64 | 0.39 | 0.76 | 0.17 | 0.37 | 0.41 | Dougherty & Smith, 2006 |
| *Lithobates clamitans* | Ranidae | Larvae | Laboratory | Road de-icers | 7 | 5 | 5 | 0.64 | 0.39 | 0.64 | 0.43 | 0.00 | 0.40 | Dougherty & Smith, 2006 |
| *Lithobates clamitans* | Ranidae | Larvae | Laboratory | Road de-icers | 7 | 5 | 5 | 0.64 | 0.39 | 0.24 | 0.36 | -0.97 | 0.45 | Dougherty & Smith, 2006 |
| *Lithobates clamitans* | Ranidae | Larvae | Laboratory | Road de-icers | 7 | 5 | 5 | 0.64 | 0.39 | 0.32 | 0.11 | -1.02 | 0.45 | Dougherty & Smith, 2006 |
| *Lithobates clamitans* | Ranidae | Larvae | Laboratory | Road de-icers | 7 | 5 | 5 | 0.92 | 0.11 | 0.88 | 0.18 | -0.24 | 0.40 | Dougherty & Smith, 2006 |
| *Lithobates clamitans* | Ranidae | Larvae | Laboratory | Road de-icers | 7 | 5 | 5 | 0.92 | 0.11 | 0.84 | 0.26 | -0.36 | 0.41 | Dougherty & Smith, 2006 |
| *Lithobates clamitans* | Ranidae | Larvae | Laboratory | Road de-icers | 7 | 5 | 5 | 0.92 | 0.11 | 0.88 | 0.18 | -0.24 | 0.40 | Dougherty & Smith, 2006 |
| *Lithobates clamitans* | Ranidae | Larvae | Laboratory | Road de-icers | 7 | 5 | 5 | 0.92 | 0.11 | 0.88 | 0.27 | -0.18 | 0.40 | Dougherty & Smith, 2006 |
| *Lithobates clamitans* | Ranidae | Larvae | Laboratory | Road de-icers | 7 | 5 | 5 | 0.92 | 0.11 | 0.84 | 0.17 | -0.51 | 0.41 | Dougherty & Smith, 2006 |
| *Lithobates clamitans* | Ranidae | Larvae | Laboratory | Road de-icers | 7 | 5 | 5 | 0.92 | 0.11 | 0.64 | 0.17 | -1.79 | 0.56 | Dougherty & Smith, 2006 |
| *Anaxyrus americanus* | Bufonidae | Larvae | Laboratory | Road de-icers | 7 | 5 | 5 | 1.00 | 0.00 | 0.64 | 0.26 | -1.76 | 0.56 | Dougherty & Smith, 2006 |
| *Anaxyrus americanus* | Bufonidae | Larvae | Laboratory | Road de-icers | 7 | 5 | 5 | 1.00 | 0.00 | 0.56 | 0.36 | -1.57 | 0.52 | Dougherty & Smith, 2006 |
| *Anaxyrus americanus* | Bufonidae | Larvae | Laboratory | Road de-icers | 7 | 5 | 5 | 1.00 | 0.00 | 0.60 | 0.20 | -2.55 | 0.73 | Dougherty & Smith, 2006 |
| *Anaxyrus americanus* | Bufonidae | Larvae | Laboratory | Road de-icers | 7 | 5 | 5 | 1.00 | 0.00 | 0.40 | 0.47 | -1.63 | 0.53 | Dougherty & Smith, 2006 |
| *Anaxyrus americanus* | Bufonidae | Larvae | Laboratory | Road de-icers | 7 | 5 | 5 | 1.00 | 0.00 | 0.60 | 0.40 | -1.28 | 0.48 | Dougherty & Smith, 2006 |
| *Xenopus laevis* | Pipidae | Larvae | Laboratory | Road de-icers | 7 | 5 | 5 | 1.00 | 0.00 | 0.96 | 0.09 | -0.57 | 0.42 | Dougherty & Smith, 2006 |
| *Xenopus laevis* | Pipidae | Larvae | Laboratory | Road de-icers | 7 | 5 | 5 | 1.00 | 0.00 | 0.92 | 0.11 | -0.93 | 0.44 | Dougherty & Smith, 2006 |
| *Xenopus laevis* | Pipidae | Larvae | Laboratory | Road de-icers | 7 | 5 | 5 | 1.00 | 0.00 | 0.92 | 0.11 | -0.93 | 0.44 | Dougherty & Smith, 2006 |
| *Xenopus laevis* | Pipidae | Larvae | Laboratory | Road de-icers | 7 | 5 | 5 | 1.00 | 0.00 | 0.92 | 0.11 | -0.93 | 0.44 | Dougherty & Smith, 2006 |
| *Xenopus laevis* | Pipidae | Larvae | Laboratory | Road de-icers | 7 | 5 | 5 | 1.00 | 0.00 | 0.88 | 0.18 | -0.86 | 0.44 | Dougherty & Smith, 2006 |
| *Xenopus laevis* | Pipidae | Larvae | Laboratory | Road de-icers | 7 | 5 | 5 | 1.00 | 0.00 | 0.96 | 0.09 | -0.57 | 0.42 | Dougherty & Smith, 2006 |
| *Xenopus laevis* | Pipidae | Larvae | Laboratory | Road de-icers | 7 | 5 | 5 | 1.00 | 0.00 | 0.96 | 0.09 | -0.57 | 0.42 | Dougherty & Smith, 2006 |
| *Xenopus laevis* | Pipidae | Larvae | Laboratory | Road de-icers | 7 | 5 | 5 | 1.00 | 0.00 | 0.76 | 0.17 | -1.84 | 0.57 | Dougherty & Smith, 2006 |
| *Xenopus laevis* | Pipidae | Larvae | Laboratory | Road de-icers | 7 | 5 | 5 | 1.00 | 0.00 | 0.80 | 0.35 | -0.74 | 0.43 | Dougherty & Smith, 2006 |
| *Xenopus laevis* | Pipidae | Larvae | Laboratory | Road de-icers | 7 | 5 | 5 | 1.00 | 0.00 | 0.96 | 0.09 | -0.57 | 0.42 | Dougherty & Smith, 2006 |
| *Xenopus laevis* | Pipidae | Larvae | Laboratory | Road de-icers | 7 | 5 | 5 | 1.00 | 0.00 | 0.92 | 0.11 | -0.93 | 0.44 | Dougherty & Smith, 2006 |
| *Xenopus laevis* | Pipidae | Larvae | Laboratory | Road de-icers | 7 | 5 | 5 | 1.00 | 0.00 | 0.88 | 0.18 | -0.86 | 0.44 | Dougherty & Smith, 2006 |
| *Xenopus laevis* | Pipidae | Larvae | Laboratory | Road de-icers | 7 | 5 | 5 | 1.00 | 0.00 | 0.84 | 0.26 | -0.78 | 0.43 | Dougherty & Smith, 2006 |
| *Xenopus laevis* | Pipidae | Larvae | Laboratory | Road de-icers | 7 | 5 | 5 | 1.00 | 0.00 | 0.80 | 0.14 | -1.81 | 0.56 | Dougherty & Smith, 2006 |
| *Xenopus laevis* | Pipidae | Larvae | Laboratory | Road de-icers | 7 | 5 | 5 | 1.00 | 0.00 | 0.92 | 0.11 | -0.93 | 0.44 | Dougherty & Smith, 2006 |
| *Xenopus laevis* | Pipidae | Larvae | Laboratory | Road de-icers | 7 | 5 | 5 | 1.00 | 0.00 | 0.96 | 0.09 | -0.57 | 0.42 | Dougherty & Smith, 2006 |
| *Xenopus laevis* | Pipidae | Larvae | Laboratory | Road de-icers | 7 | 5 | 5 | 1.00 | 0.00 | 0.92 | 0.18 | -0.57 | 0.42 | Dougherty & Smith, 2006 |
| *Xenopus laevis* | Pipidae | Larvae | Laboratory | Road de-icers | 7 | 5 | 5 | 0.96 | 0.09 | 0.80 | 0.14 | -1.23 | 0.48 | Dougherty & Smith, 2006 |
| *Xenopus laevis* | Pipidae | Larvae | Laboratory | Road de-icers | 7 | 5 | 5 | 0.96 | 0.09 | 0.88 | 0.18 | -0.51 | 0.41 | Dougherty & Smith, 2006 |
| *Xenopus laevis* | Pipidae | Larvae | Laboratory | Road de-icers | 7 | 5 | 5 | 0.96 | 0.09 | 0.68 | 0.23 | -1.46 | 0.51 | Dougherty & Smith, 2006 |
| *Xenopus laevis* | Pipidae | Larvae | Laboratory | Road de-icers | 7 | 5 | 5 | 0.96 | 0.09 | 0.72 | 0.18 | -1.53 | 0.52 | Dougherty & Smith, 2006 |
| *Xenopus laevis* | Pipidae | Larvae | Laboratory | Road de-icers | 7 | 5 | 5 | 0.96 | 0.09 | 0.88 | 0.11 | -0.72 | 0.43 | Dougherty & Smith, 2006 |
| *Xenopus laevis* | Pipidae | Larvae | Laboratory | Road de-icers | 7 | 5 | 5 | 0.96 | 0.09 | 0.80 | 0.20 | -0.93 | 0.44 | Dougherty & Smith, 2006 |
| *Xenopus laevis* | Pipidae | Larvae | Laboratory | Road de-icers | 7 | 5 | 5 | 0.96 | 0.09 | 0.88 | 0.11 | -0.72 | 0.43 | Dougherty & Smith, 2006 |
| *Xenopus laevis* | Pipidae | Larvae | Laboratory | Road de-icers | 7 | 5 | 5 | 0.96 | 0.09 | 0.84 | 0.17 | -0.81 | 0.43 | Dougherty & Smith, 2006 |
| *Xenopus laevis* | Pipidae | Larvae | Laboratory | Road de-icers | 7 | 5 | 5 | 0.96 | 0.09 | 0.92 | 0.18 | -0.26 | 0.40 | Dougherty & Smith, 2006 |
| *Xenopus laevis* | Pipidae | Larvae | Laboratory | Road de-icers | 7 | 5 | 5 | 0.96 | 0.09 | 0.92 | 0.18 | -0.26 | 0.40 | Dougherty & Smith, 2006 |
| *Lithobates pipiens* | Ranidae | Larvae | Laboratory | Wastewater contaminants | 24 | 3 | 3 | 1.00 | 0.00 | 0.93 | 0.12 | -0.66 | 0.70 | Fraker & Smith, 2004 |
| *Lithobates blairi* | Ranidae | Larvae | Laboratory | Pesticides | 1 | 5 | 5 | 0.95 | 0.11 | 0.00 | 0.11 | -7.67 | 3.35 | Smith, 2001 |
| *Lithobates blairi* | Ranidae | Larvae | Laboratory | Pesticides | 1 | 5 | 5 | 0.95 | 0.11 | 0.00 | 0.11 | -7.67 | 3.35 | Smith, 2001 |
| *Lithobates blairi* | Ranidae | Larvae | Laboratory | Pesticides | 1 | 5 | 5 | 0.95 | 0.11 | 0.00 | 0.11 | -7.67 | 3.35 | Smith, 2001 |
| *Lithobates sylvaticus* | Ranidae | Larvae | Laboratory | Nitrogenous compounds | 7 | 5 | 5 | 0.82 | 0.19 | 0.00 | 0.00 | -5.58 | 1.95 | Burgett *et al.*, 2007 |
| *Triturus pygmaeus* | Salamandridae | Embryos | Laboratory | Nitrogenous compounds | 25 | 4 | 4 | 0.42 | 0.07 | 0.22 | 0.07 | -2.42 | 0.87 | Ortiz-Santaliestra *et al.*, 2007 |
| *Lithobates sylvaticus* | Ranidae | Embryos | Laboratory | Wastewater contaminants | 23 | 5 | 5 | 0.97 | 0.04 | 0.89 | 0.06 | -1.46 | 0.51 | Laposata & Dunson, 1998 |
| *Ambystoma jeffersonianum* | Ambystomatidae | Embryos | Laboratory | Wastewater contaminants | 25 | 5 | 5 | 0.97 | 0.06 | 0.96 | 0.04 | -0.24 | 0.40 | Laposata & Dunson, 1998 |
| *Ambystoma maculatum* | Ambystomatidae | Embryos | Laboratory | Wastewater contaminants | 44 | 5 | 5 | 0.89 | 0.17 | 0.93 | 0.08 | 0.27 | 0.40 | Laposata & Dunson, 1998 |
| *Anaxyrus americanus* | Bufonidae | Embryos | Laboratory | Wastewater contaminants | 23 | 5 | 5 | 0.82 | 0.07 | 0.47 | 0.07 | -4.53 | 1.42 | Laposata & Dunson, 1998 |
| *Pelophylax perezi* | Ranidae | Larvae | Laboratory | Nitrogenous compounds | 21 | 7 | 7 | 1.00 | 0.00 | 0.86 | 0.38 | -0.50 | 0.29 | Egea-Serrano *et al*., 2010 |
| *Pelophylax perezi* | Ranidae | Larvae | Laboratory | Nitrogenous compounds | 21 | 7 | 7 | 1.00 | 0.00 | 0.86 | 0.38 | -0.50 | 0.29 | Egea-Serrano *et al*., 2010 |
| *Pelophylax perezi* | Ranidae | Larvae | Laboratory | Nitrogenous compounds | 21 | 7 | 7 | 1.00 | 0.00 | 0.71 | 0.49 | -0.78 | 0.31 | Egea-Serrano *et al*., 2010 |
| *Pelophylax perezi* | Ranidae | Larvae | Laboratory | Nitrogenous compounds | 21 | 7 | 7 | 1.00 | 0.00 | 0.57 | 0.53 | -1.06 | 0.33 | Egea-Serrano *et al*., 2010 |
| *Pelophylax perezi* | Ranidae | Larvae | Laboratory | Nitrogenous compounds | 21 | 7 | 7 | 1.00 | 0.00 | 0.86 | 0.38 | -0.50 | 0.29 | Egea-Serrano *et al*., 2010 |
| *Pelophylax perezi* | Ranidae | Larvae | Laboratory | Nitrogenous compounds | 21 | 7 | 7 | 1.00 | 0.00 | 0.71 | 0.49 | -0.78 | 0.31 | Egea-Serrano *et al*., 2010 |
| *Pelophylax perezi* | Ranidae | Larvae | Laboratory | Nitrogenous compounds | 21 | 7 | 7 | 1.00 | 0.00 | 0.86 | 0.38 | -0.50 | 0.29 | Egea-Serrano *et al*., 2010 |
| *Pelophylax perezi* | Ranidae | Larvae | Laboratory | Nitrogenous compounds | 21 | 5 | 5 | 1.00 | 0.00 | 0.80 | 0.45 | -0.57 | 0.42 | Egea-Serrano *et al*., 2010 |
| *Pelophylax perezi* | Ranidae | Larvae | Laboratory | Nitrogenous compounds | 21 | 5 | 5 | 1.00 | 0.00 | 0.80 | 0.45 | -0.57 | 0.42 | Egea-Serrano *et al*., 2010 |
| *Pelophylax perezi* | Ranidae | Larvae | Laboratory | Nitrogenous compounds | 21 | 5 | 5 | 1.00 | 0.00 | 0.80 | 0.45 | -0.57 | 0.42 | Egea-Serrano *et al*., 2010 |
| *Pelophylax perezi* | Ranidae | Larvae | Laboratory | Nitrogenous compounds | 21 | 5 | 5 | 1.00 | 0.00 | 0.80 | 0.45 | -0.57 | 0.42 | Egea-Serrano *et al*., 2010 |
| *Pelophylax perezi* | Ranidae | Larvae | Laboratory | Nitrogenous compounds | 21 | 5 | 5 | 1.00 | 0.00 | 0.80 | 0.45 | -0.57 | 0.42 | Egea-Serrano *et al*., 2010 |
| *Pelophylax perezi* | Ranidae | Larvae | Mesocosms | Nitrogenous compounds | 21 | 20 | 20 | 0.95 | 0.22 | 0.95 | 0.22 | 0.00 | 0.10 | Egea-Serrano *et al*., 2010 |
| *Pelophylax perezi* | Ranidae | Larvae | Mesocosms | Nitrogenous compounds | 21 | 20 | 20 | 0.95 | 0.22 | 0.95 | 0.22 | 0.00 | 0.10 | Egea-Serrano *et al*., 2010 |
| *Pelophylax perezi* | Ranidae | Larvae | Mesocosms | Nitrogenous compounds | 21 | 20 | 20 | 1.00 | 0.00 | 0.90 | 0.31 | -0.45 | 0.10 | Egea-Serrano *et al*., 2010 |
| *Pelophylax perezi* | Ranidae | Larvae | Enclosures | Nitrogenous compounds | 21 | 8 | 8 | 1.00 | 0.00 | 0.88 | 0.35 | -0.47 | 0.26 | Egea-Serrano *et al*., 2010 |
| *Pelophylax perezi* | Ranidae | Larvae | Enclosures | Nitrogenous compounds | 21 | 8 | 8 | 1.00 | 0.00 | 0.88 | 0.35 | -0.47 | 0.26 | Egea-Serrano *et al*., 2010 |
| *Pelophylax perezi* | Ranidae | Larvae | Enclosures | Nitrogenous compounds | 21 | 8 | 8 | 1.00 | 0.00 | 0.63 | 0.52 | -0.97 | 0.28 | Egea-Serrano *et al*., 2010 |
| *Pelophylax perezi* | Ranidae | Larvae | Enclosures | Nitrogenous compounds | 21 | 8 | 8 | 1.00 | 0.00 | 0.63 | 0.52 | -0.97 | 0.28 | Egea-Serrano *et al*., 2010 |
| *Pelophylax perezi* | Ranidae | Larvae | Enclosures | Nitrogenous compounds | 21 | 8 | 8 | 0.88 | 0.35 | 0.88 | 0.35 | 0.00 | 0.25 | Egea-Serrano *et al*., 2010 |
| *Pelophylax perezi* | Ranidae | Larvae | Enclosures | Nitrogenous compounds | 21 | 8 | 8 | 0.88 | 0.35 | 0.63 | 0.52 | -0.53 | 0.26 | Egea-Serrano *et al*., 2010 |
| *Lithobates pipiens* | Ranidae | Embryos | Laboratory | Heavy metals | 154 | 5 | 5 | 0.71 | 0.13 | 0.75 | 0.11 | 0.25 | 0.40 | Chen *et al.*, 2006 |
| *Lithobates pipiens* | Ranidae | Embryos | Laboratory | Heavy metals | 154 | 5 | 5 | 0.71 | 0.13 | 0.81 | 0.14 | 0.63 | 0.42 | Chen *et al.*, 2006 |
| *Lithobates pipiens* | Ranidae | Embryos | Laboratory | Heavy metals | 154 | 5 | 5 | 0.71 | 0.13 | 0.59 | 0.11 | -0.94 | 0.44 | Chen *et al.*, 2006 |
| *Lithobates pipiens* | Ranidae | Embryos | Laboratory | Pesticides | 154 | 4 | 4 | 0.61 | 0.16 | 0.54 | 0.27 | -0.27 | 0.50 | Chen *et al.*, 2007 |
| *Lithobates pipiens* | Ranidae | Embryos | Laboratory | Pesticides | 154 | 4 | 4 | 0.61 | 0.16 | 0.41 | 0.29 | -0.77 | 0.54 | Chen *et al.*, 2007 |
| *Lithobates pipiens* | Ranidae | Embryos | Laboratory | Pesticides | 154 | 4 | 4 | 0.61 | 0.16 | 0.06 | 0.07 | -4.00 | 1.50 | Chen *et al.*, 2007 |
| *Hyla versicolor* | Hylidae | Larvae | Mesocosms | Pesticides | 78 | 4 | 4 | 0.90 | 0.07 | 0.87 | 0.14 | -0.27 | 0.50 | Relyea, 2008 |
| *Hyla versicolor* | Hylidae | Larvae | Mesocosms | Pesticides | 78 | 4 | 4 | 0.90 | 0.07 | 0.93 | 0.10 | 0.26 | 0.50 | Relyea, 2008 |
| *Hyla versicolor* | Hylidae | Larvae | Mesocosms | Pesticides | 78 | 4 | 4 | 0.90 | 0.07 | 0.89 | 0.10 | -0.17 | 0.50 | Relyea, 2008 |
| *Hyla versicolor* | Hylidae | Larvae | Mesocosms | Pesticides | 78 | 4 | 4 | 0.90 | 0.07 | 0.92 | 0.05 | 0.25 | 0.50 | Relyea, 2008 |

| Mass (mg) | | | | | | | | | | | | | | |
| --- | --- | --- | --- | --- | --- | --- | --- | --- | --- | --- | --- | --- | --- | --- |
|  |  |  |  |  |  | n | | Control treatment | | Polluted treatment | |  |  |  |
| Species | Family | Developmental stage | Experimental venue | Pollutant | Days of exposure | Control | Polluted | Mean | SD | Mean | SD | d | Var(d) | Reference |
| *Lithobates catesbeianus* | Ranidae | Larvae | Laboratory | Nitrogenous compounds | 15 | 4 | 4 | 19.00 | 2.00 | 15.00 | 1.80 | -1.83 | 0.71 | Smith *et al.*, 2005 |
| *Lithobates clamitans* | Ranidae | Larvae | Laboratory | Nitrogenous compounds | 15 | 4 | 4 | 16.00 | 1.80 | 15.00 | 4.00 | -0.28 | 0.50 | Smith *et al.*, 2005 |
| *Lithobates catesbeianus* | Ranidae | Larvae | Laboratory | Nitrogenous compounds | 15 | 4 | 4 | 34.00 | 6.00 | 29.00 | 6.00 | -0.72 | 0.53 | Smith *et al.*, 2004 |
| *Lithobates catesbeianus* | Ranidae | Larvae | Mesocosms | Nitrogenous compounds | 42 | 3 | 3 | 660.00 | 74.48 | 800.00 | 363.73 | 0.43 | 0.68 | Smith *et al.*, 2006 |
| *Lithobates clamitans* | Ranidae | Larvae | Mesocosms | Nitrogenous compounds | 42 | 3 | 3 | 510.00 | 207.85 | 1000.00 | 467.65 | 1.08 | 0.76 | Smith *et al.*, 2006 |
| *Hyla versicolor* | Hylidae | Embryos | Laboratory | Nitrogenous compounds | 15 | 4 | 4 | 27.00 | 6.00 | 26.00 | 2.00 | -0.19 | 0.50 | Vaala *et al.*, 2004 |
| *Pseudacris regilla* | Hylidae | Embryos | Laboratory | Nitrogenous compounds | 10 | 3 | 3 | 19.10 | 1.21 | 13.70 | 0.17 | -4.99 | 2.74 | Schuytema & Nebeker, 1999 |
| *Pseudacris regilla* | Hylidae | Embryos | Laboratory | Nitrogenous compounds | 10 | 3 | 3 | 18.90 | 1.73 | 16.70 | 0.87 | -1.29 | 0.80 | Schuytema & Nebeker, 1999 |
| *Pseudacris regilla* | Hylidae | Embryos | Laboratory | Nitrogenous compounds | 10 | 3 | 3 | 16.40 | 0.52 | 15.30 | 1.04 | -1.07 | 0.76 | Schuytema & Nebeker, 1999 |
| *Pseudacris regilla* | Hylidae | Embryos | Laboratory | Nitrogenous compounds | 10 | 3 | 3 | 19.20 | 1.04 | 17.10 | 1.73 | -1.18 | 0.78 | Schuytema & Nebeker, 1999 |
| *Xenopus laevis* | Pipidae | Embryos | Laboratory | Nitrogenous compounds | 5 | 3 | 3 | 7.70 | 0.69 | 6.40 | 0.69 | -1.50 | 0.85 | Schuytema & Nebeker, 1999 |
| *Xenopus laevis* | Pipidae | Embryos | Laboratory | Nitrogenous compounds | 5 | 3 | 3 | 8.10 | 0.69 | 7.20 | 0.69 | -1.04 | 0.76 | Schuytema & Nebeker, 1999 |
| *Xenopus laevis* | Pipidae | Embryos | Laboratory | Nitrogenous compounds | 5 | 3 | 3 | 8.70 | 0.69 | 7.80 | 1.04 | -0.82 | 0.72 | Schuytema & Nebeker, 1999 |
| *Xenopus laevis* | Pipidae | Embryos | Laboratory | Nitrogenous compounds | 5 | 3 | 3 | 7.90 | 0.35 | 6.70 | 0.35 | -2.77 | 1.31 | Schuytema & Nebeker, 1999 |
| *Xenopus laevis* | Pipidae | Embryos | Laboratory | Nitrogenous compounds | 4 | 3 | 3 | 6.40 | 0.17 | 6.00 | 0.35 | -1.17 | 0.78 | Schuytema & Nebeker, 1999 |
| *Xenopus laevis* | Pipidae | Embryos | Laboratory | Nitrogenous compounds | 4 | 3 | 3 | 6.70 | 0.52 | 6.20 | 0.52 | -0.77 | 0.72 | Schuytema & Nebeker, 1999 |
| *Xenopus laevis* | Pipidae | Embryos | Laboratory | Nitrogenous compounds | 4 | 3 | 3 | 7.10 | 0.00 | 6.50 | 0.17 | -3.92 | 1.95 | Schuytema & Nebeker, 1999 |
| *Xenopus laevis* | Pipidae | Embryos | Laboratory | Nitrogenous compounds | 4 | 3 | 3 | 6.40 | 0.00 | 6.50 | 0.35 | 0.33 | 0.68 | Schuytema & Nebeker, 1999 |
| *Rana aurora* | Ranidae | Embryos | Laboratory | Nitrogenous compounds | 16 | 4 | 4 | 25.80 | 1.40 | 24.30 | 2.00 | -0.76 | 0.54 | Schuytema & Nebeker, 1999 |
| *Rana aurora* | Ranidae | Embryos | Laboratory | Nitrogenous compounds | 16 | 4 | 4 | 27.20 | 5.00 | 26.00 | 1.80 | -0.28 | 0.50 | Schuytema & Nebeker, 1999 |
| *Lithobates sphenocephalus* | Ranidae | Larvae | Laboratory | Nitrogenous compounds | 105 | 15 | 2 | 1550.00 | 430.00 | 1510.00 | 350.00 | -0.09 | 0.57 | Ortiz-Santaliestra & Sparling, 2007 |
| *Lithobates sphenocephalus* | Ranidae | Larvae | Laboratory | Wastewater contaminants | 105 | 15 | 5 | 1550.00 | 430.00 | 1460.00 | 370.00 | -0.21 | 0.27 | Ortiz-Santaliestra & Sparling, 2007 |
| *Xenopus laevis* | Pipidae | Larvae | Laboratory | Pesticides | 78 | 11 | 11 | 328.00 | 13.27 | 347.00 | 6.30 | 1.76 | 0.25 | Carr *et al.*, 2003 |
| *Xenopus laevis* | Pipidae | Larvae | Laboratory | Pesticides | 78 | 11 | 11 | 320.00 | 26.53 | 356.00 | 23.22 | 1.39 | 0.23 | Carr *et al.*, 2003 |
| *Lithobates sphenocephalus* | Ranidae | Larvae | Laboratory | Pesticides | 12 | 4 | 4 | 198.49 | 28.78 | 221.97 | 27.28 | 0.73 | 0.53 | Widder & Bidwell, 2006 |
| *Lithobates sphenocephalus* | Ranidae | Larvae | Mesocosms | Pesticides | 12 | 4 | 4 | 362.50 | 75.00 | 323.77 | 122.50 | -0.33 | 0.51 | Widder & Bidwell, 2006 |
| *Hyla chrysoscelis* | Hylidae | Larvae | Laboratory | Pesticides | 12 | 4 | 4 | 98.00 | 18.00 | 82.00 | 6.00 | -1.04 | 0.57 | Widder & Bidwell, 2008 |
| *Lithobates sphenocephalus* | Ranidae | Larvae | Laboratory | Pesticides | 12 | 4 | 4 | 194.00 | 30.00 | 185.00 | 18.00 | -0.32 | 0.51 | Widder & Bidwell, 2008 |
| *Acris crepitans* | Hylidae | Larvae | Laboratory | Pesticides | 4 | 4 | 4 | 169.00 | 20.00 | 158.00 | 38.00 | -0.32 | 0.51 | Widder & Bidwell, 2008 |
| *Gastrophryne olivacea* | Microhylidae | Larvae | Laboratory | Pesticides | 4 | 4 | 4 | 175.00 | 20.00 | 162.00 | 10.00 | -0.71 | 0.53 | Widder & Bidwell, 2008 |
| *Lithobates catesbeianus* | Ranidae | Larvae | Laboratory | Nitrogenous compounds | 15 | 4 | 4 | 19.00 | 2.00 | 17.00 | 4.00 | -0.55 | 0.52 | Smith *et al.*, 2005 |
| *Lithobates catesbeianus* | Ranidae | Larvae | Laboratory | Nitrogenous compounds | 15 | 4 | 4 | 19.00 | 2.00 | 19.00 | 2.00 | 0.00 | 0.50 | Smith *et al.*, 2005 |
| *Lithobates catesbeianus* | Ranidae | Larvae | Laboratory | Nitrogenous compounds | 15 | 4 | 4 | 19.00 | 2.00 | 18.00 | 2.00 | -0.43 | 0.51 | Smith *et al.*, 2005 |
| *Lithobates catesbeianus* | Ranidae | Larvae | Laboratory | Nitrogenous compounds | 15 | 4 | 4 | 19.00 | 2.00 | 14.00 | 2.00 | -2.17 | 0.80 | Smith *et al.*, 2005 |
| *Lithobates clamitans* | Ranidae | Larvae | Laboratory | Nitrogenous compounds | 15 | 4 | 4 | 16.00 | 1.80 | 15.00 | 1.40 | -0.54 | 0.52 | Smith *et al.*, 2005 |
| *Lithobates clamitans* | Ranidae | Larvae | Laboratory | Nitrogenous compounds | 15 | 4 | 4 | 16.00 | 1.80 | 13.00 | 4.00 | -0.84 | 0.54 | Smith *et al.*, 2005 |
| *Lithobates clamitans* | Ranidae | Larvae | Laboratory | Nitrogenous compounds | 15 | 4 | 4 | 16.00 | 1.80 | 13.00 | 1.60 | -1.53 | 0.65 | Smith *et al.*, 2005 |
| *Lithobates clamitans* | Ranidae | Larvae | Laboratory | Nitrogenous compounds | 15 | 4 | 4 | 16.00 | 1.80 | 14.00 | 0.00 | -1.37 | 0.62 | Smith *et al.*, 2005 |
| *Lithobates catesbeianus* | Ranidae | Larvae | Laboratory | Nitrogenous compounds | 15 | 4 | 4 | 34.00 | 6.00 | 25.00 | 4.00 | -1.53 | 0.65 | Smith *et al.*, 2004 |
| *Lithobates catesbeianus* | Ranidae | Larvae | Laboratory | Nitrogenous compounds | 15 | 4 | 4 | 34.00 | 6.00 | 34.00 | 4.00 | 0.00 | 0.50 | Smith *et al.*, 2004 |
| *Lithobates catesbeianus* | Ranidae | Larvae | Laboratory | Nitrogenous compounds | 15 | 4 | 4 | 34.00 | 6.00 | 26.00 | 6.00 | -1.16 | 0.58 | Smith *et al.*, 2004 |
| *Lithobates catesbeianus* | Ranidae | Larvae | Laboratory | Nitrogenous compounds | 15 | 4 | 4 | 34.00 | 6.00 | 29.00 | 4.00 | -0.85 | 0.55 | Smith *et al.*, 2004 |
| *Xenopus laevis* | Pipidae | Larvae | Laboratory | Wastewater contaminants | 11 | 5 | 5 | 46.00 | 4.47 | 64.00 | 13.42 | 1.63 | 0.53 | Fraker & Smith, 2005 |
| *Xenopus laevis* | Pipidae | Larvae | Laboratory | Wastewater contaminants | 11 | 5 | 5 | 46.00 | 4.47 | 49.00 | 17.89 | 0.21 | 0.40 | Fraker & Smith, 2005 |
| *Xenopus laevis* | Pipidae | Larvae | Laboratory | Wastewater contaminants | 11 | 4 | 4 | 48.00 | 2.00 | 47.00 | 4.00 | -0.27 | 0.50 | Fraker & Smith, 2005 |
| *Xenopus laevis* | Pipidae | Larvae | Laboratory | Wastewater contaminants | 11 | 4 | 4 | 48.00 | 2.00 | 50.00 | 2.00 | 0.87 | 0.55 | Fraker & Smith, 2005 |
| *Hyla versicolor* | Hylidae | Larvae | Laboratory | Nitrogenous compounds | 15 | 4 | 4 | 27.00 | 6.00 | 30.00 | 4.00 | 0.51 | 0.52 | Vaala *et al.*, 2004 |
| *Hyla versicolor* | Hylidae | Larvae | Laboratory | Nitrogenous compounds | 15 | 4 | 4 | 27.00 | 6.00 | 28.00 | 2.00 | 0.19 | 0.50 | Vaala *et al.*, 2004 |
| *Hyla versicolor* | Hylidae | Larvae | Laboratory | Nitrogenous compounds | 15 | 4 | 4 | 27.00 | 6.00 | 26.00 | 6.00 | -0.14 | 0.50 | Vaala *et al.*, 2004 |
| *Hyla versicolor* | Hylidae | Larvae | Laboratory | Nitrogenous compounds | 15 | 4 | 4 | 27.00 | 6.00 | 25.00 | 4.00 | -0.34 | 0.51 | Vaala *et al.*, 2004 |
| *Pseudacris regilla* | Hylidae | Embryos | Laboratory | Nitrogenous compounds | 10 | 3 | 3 | 19.10 | 1.21 | 17.20 | 1.04 | -1.35 | 0.82 | Schuytema & Nebeker, 1999 |
| *Pseudacris regilla* | Hylidae | Embryos | Laboratory | Nitrogenous compounds | 10 | 3 | 3 | 19.10 | 1.21 | 11.80 | 1.56 | -4.18 | 2.12 | Schuytema & Nebeker, 1999 |
| *Pseudacris regilla* | Hylidae | Embryos | Laboratory | Nitrogenous compounds | 10 | 3 | 3 | 19.10 | 1.21 | 5.90 | 1.04 | -9.35 | 7.96 | Schuytema & Nebeker, 1999 |
| *Pseudacris regilla* | Hylidae | Embryos | Laboratory | Nitrogenous compounds | 10 | 3 | 3 | 18.90 | 1.73 | 16.70 | 0.87 | -1.29 | 0.80 | Schuytema & Nebeker, 1999 |
| *Pseudacris regilla* | Hylidae | Embryos | Laboratory | Nitrogenous compounds | 10 | 3 | 3 | 18.90 | 1.73 | 15.20 | 0.17 | -2.40 | 1.15 | Schuytema & Nebeker, 1999 |
| *Pseudacris regilla* | Hylidae | Embryos | Laboratory | Nitrogenous compounds | 10 | 3 | 3 | 18.90 | 1.73 | 6.40 | 2.42 | -4.75 | 2.54 | Schuytema & Nebeker, 1999 |
| *Pseudacris regilla* | Hylidae | Embryos | Laboratory | Nitrogenous compounds | 10 | 3 | 3 | 16.40 | 0.52 | 16.20 | 0.52 | -0.31 | 0.67 | Schuytema & Nebeker, 1999 |
| *Pseudacris regilla* | Hylidae | Embryos | Laboratory | Nitrogenous compounds | 10 | 3 | 3 | 16.40 | 0.52 | 12.60 | 2.08 | -2.01 | 1.00 | Schuytema & Nebeker, 1999 |
| *Pseudacris regilla* | Hylidae | Embryos | Laboratory | Nitrogenous compounds | 10 | 3 | 3 | 16.40 | 0.52 | 10.20 | 1.39 | -4.74 | 2.54 | Schuytema & Nebeker, 1999 |
| *Pseudacris regilla* | Hylidae | Embryos | Laboratory | Nitrogenous compounds | 10 | 3 | 3 | 19.20 | 1.04 | 16.10 | 1.73 | -1.74 | 0.92 | Schuytema & Nebeker, 1999 |
| *Xenopus laevis* | Pipidae | Embryos | Laboratory | Nitrogenous compounds | 5 | 3 | 3 | 7.70 | 0.69 | 7.30 | 0.35 | -0.58 | 0.70 | Schuytema & Nebeker, 1999 |
| *Xenopus laevis* | Pipidae | Embryos | Laboratory | Nitrogenous compounds | 5 | 3 | 3 | 7.70 | 0.69 | 4.90 | 1.73 | -1.70 | 0.91 | Schuytema & Nebeker, 1999 |
| *Xenopus laevis* | Pipidae | Embryos | Laboratory | Nitrogenous compounds | 5 | 3 | 3 | 7.70 | 0.69 | 3.60 | 0.17 | -6.50 | 4.18 | Schuytema & Nebeker, 1999 |
| *Xenopus laevis* | Pipidae | Embryos | Laboratory | Nitrogenous compounds | 5 | 3 | 3 | 8.10 | 0.69 | 7.70 | 0.35 | -0.58 | 0.70 | Schuytema & Nebeker, 1999 |
| *Xenopus laevis* | Pipidae | Embryos | Laboratory | Nitrogenous compounds | 5 | 3 | 3 | 8.10 | 0.69 | 6.60 | 0.17 | -2.38 | 1.14 | Schuytema & Nebeker, 1999 |
| *Xenopus laevis* | Pipidae | Embryos | Laboratory | Nitrogenous compounds | 5 | 3 | 3 | 8.10 | 0.69 | 4.70 | 0.17 | -5.39 | 3.08 | Schuytema & Nebeker, 1999 |
| *Xenopus laevis* | Pipidae | Embryos | Laboratory | Nitrogenous compounds | 5 | 3 | 3 | 8.70 | 0.69 | 8.10 | 0.35 | -0.88 | 0.73 | Schuytema & Nebeker, 1999 |
| *Xenopus laevis* | Pipidae | Embryos | Laboratory | Nitrogenous compounds | 5 | 3 | 3 | 8.70 | 0.69 | 5.80 | 0.17 | -4.59 | 2.43 | Schuytema & Nebeker, 1999 |
| *Xenopus laevis* | Pipidae | Embryos | Laboratory | Nitrogenous compounds | 5 | 3 | 3 | 8.70 | 0.69 | 4.10 | 0.35 | -6.72 | 4.43 | Schuytema & Nebeker, 1999 |
| *Xenopus laevis* | Pipidae | Embryos | Laboratory | Nitrogenous compounds | 5 | 3 | 3 | 7.90 | 0.35 | 7.80 | 0.52 | -0.18 | 0.67 | Schuytema & Nebeker, 1999 |
| *Xenopus laevis* | Pipidae | Embryos | Laboratory | Nitrogenous compounds | 4 | 3 | 3 | 6.40 | 0.17 | 6.40 | 0.35 | 0.00 | 0.67 | Schuytema & Nebeker, 1999 |
| *Xenopus laevis* | Pipidae | Embryos | Laboratory | Nitrogenous compounds | 4 | 3 | 3 | 6.40 | 0.17 | 6.10 | 0.35 | -0.88 | 0.73 | Schuytema & Nebeker, 1999 |
| *Xenopus laevis* | Pipidae | Embryos | Laboratory | Nitrogenous compounds | 4 | 3 | 3 | 6.40 | 0.17 | 5.50 | 0.35 | -2.63 | 1.24 | Schuytema & Nebeker, 1999 |
| *Xenopus laevis* | Pipidae | Embryos | Laboratory | Nitrogenous compounds | 4 | 3 | 3 | 6.70 | 0.52 | 5.60 | 0.52 | -1.69 | 0.91 | Schuytema & Nebeker, 1999 |
| *Xenopus laevis* | Pipidae | Embryos | Laboratory | Nitrogenous compounds | 4 | 3 | 3 | 6.70 | 0.52 | 5.20 | 0.35 | -2.72 | 1.28 | Schuytema & Nebeker, 1999 |
| *Xenopus laevis* | Pipidae | Embryos | Laboratory | Nitrogenous compounds | 4 | 3 | 3 | 7.10 | 0.00 | 6.80 | 0.35 | -0.98 | 0.75 | Schuytema & Nebeker, 1999 |
| *Xenopus laevis* | Pipidae | Embryos | Laboratory | Nitrogenous compounds | 4 | 3 | 3 | 7.10 | 0.00 | 6.50 | 0.35 | -1.96 | 0.99 | Schuytema & Nebeker, 1999 |
| *Xenopus laevis* | Pipidae | Embryos | Laboratory | Nitrogenous compounds | 4 | 3 | 3 | 7.10 | 0.00 | 6.10 | 0.35 | -3.27 | 1.56 | Schuytema & Nebeker, 1999 |
| *Xenopus laevis* | Pipidae | Embryos | Laboratory | Nitrogenous compounds | 4 | 3 | 3 | 6.40 | 0.00 | 6.40 | 0.35 | 0.00 | 0.67 | Schuytema & Nebeker, 1999 |
| *Rana aurora* | Ranidae | Embryos | Laboratory | Nitrogenous compounds | 16 | 4 | 4 | 25.80 | 1.40 | 23.00 | 2.00 | -1.41 | 0.62 | Schuytema & Nebeker, 1999 |
| *Rana aurora* | Ranidae | Embryos | Laboratory | Nitrogenous compounds | 16 | 4 | 4 | 25.80 | 1.40 | 20.40 | 0.80 | -4.12 | 1.56 | Schuytema & Nebeker, 1999 |
| *Rana aurora* | Ranidae | Embryos | Laboratory | Nitrogenous compounds | 16 | 4 | 4 | 25.80 | 1.40 | 16.70 | 2.00 | -4.58 | 1.81 | Schuytema & Nebeker, 1999 |
| *Rana aurora* | Ranidae | Embryos | Laboratory | Nitrogenous compounds | 16 | 4 | 4 | 27.20 | 5.00 | 23.70 | 0.60 | -0.85 | 0.55 | Schuytema & Nebeker, 1999 |
| *Xenopus laevis* | Pipidae | Larvae | Laboratory | Pesticides | 78 | 11 | 11 | 328.00 | 13.27 | 311.00 | 19.90 | -0.97 | 0.20 | Carr *et al.*, 2003 |
| *Xenopus laevis* | Pipidae | Larvae | Laboratory | Pesticides | 78 | 11 | 11 | 320.00 | 26.53 | 325.00 | 33.17 | 0.16 | 0.18 | Carr *et al.*, 2003 |
| *Xenopus laevis* | Pipidae | Larvae | Laboratory | Pesticides | 78 | 11 | 11 | 328.00 | 13.27 | 323.00 | 33.17 | -0.19 | 0.18 | Carr *et al.*, 2003 |
| *Xenopus laevis* | Pipidae | Larvae | Laboratory | Pesticides | 78 | 11 | 11 | 320.00 | 26.53 | 327.00 | 33.17 | 0.22 | 0.18 | Carr *et al.*, 2003 |
| *Lithobates sphenocephalus* | Ranidae | Larvae | Laboratory | Pesticides | 12 | 4 | 4 | 198.49 | 28.78 | 190.15 | 16.66 | -0.31 | 0.51 | Widder & Bidwell, 2006 |
| *Lithobates sphenocephalus* | Ranidae | Larvae | Laboratory | Pesticides | 12 | 4 | 4 | 198.49 | 28.78 | 184.85 | 10.60 | -0.55 | 0.52 | Widder & Bidwell, 2006 |
| *Lithobates sphenocephalus* | Ranidae | Larvae | Laboratory | Pesticides | 12 | 4 | 4 | 198.49 | 28.78 | 187.88 | 37.88 | -0.27 | 0.50 | Widder & Bidwell, 2006 |
| *Hyla chrysoscelis* | Hylidae | Larvae | Laboratory | Pesticides | 12 | 4 | 4 | 98.00 | 18.00 | 81.00 | 4.00 | -1.13 | 0.58 | Widder & Bidwell, 2008 |
| *Hyla chrysoscelis* | Hylidae | Larvae | Laboratory | Pesticides | 12 | 4 | 4 | 98.00 | 18.00 | 83.00 | 30.00 | -0.53 | 0.52 | Widder & Bidwell, 2008 |
| *Hyla chrysoscelis* | Hylidae | Larvae | Laboratory | Pesticides | 12 | 4 | 4 | 98.00 | 18.00 | 62.00 | 8.00 | -2.25 | 0.82 | Widder & Bidwell, 2008 |
| *Lithobates sphenocephalus* | Ranidae | Larvae | Laboratory | Pesticides | 12 | 4 | 4 | 194.00 | 30.00 | 217.00 | 28.00 | 0.69 | 0.53 | Widder & Bidwell, 2008 |
| *Lithobates sphenocephalus* | Ranidae | Larvae | Laboratory | Pesticides | 12 | 4 | 4 | 194.00 | 30.00 | 180.00 | 12.00 | -0.53 | 0.52 | Widder & Bidwell, 2008 |
| *Lithobates sphenocephalus* | Ranidae | Larvae | Laboratory | Pesticides | 12 | 4 | 4 | 194.00 | 30.00 | 183.00 | 38.00 | -0.28 | 0.50 | Widder & Bidwell, 2008 |
| *Acris crepitans* | Hylidae | Larvae | Laboratory | Pesticides | 4 | 4 | 4 | 169.00 | 20.00 | 172.00 | 12.00 | 0.16 | 0.50 | Widder & Bidwell, 2008 |
| *Acris crepitans* | Hylidae | Larvae | Laboratory | Pesticides | 4 | 4 | 4 | 169.00 | 20.00 | 177.00 | 12.00 | 0.42 | 0.51 | Widder & Bidwell, 2008 |
| *Acris crepitans* | Hylidae | Larvae | Laboratory | Pesticides | 4 | 4 | 4 | 169.00 | 20.00 | 161.00 | 8.00 | -0.46 | 0.51 | Widder & Bidwell, 2008 |
| *Gastrophryne olivacea* | Microhylidae | Larvae | Laboratory | Pesticides | 4 | 4 | 4 | 175.00 | 20.00 | 178.00 | 24.00 | 0.12 | 0.50 | Widder & Bidwell, 2008 |
| *Gastrophryne olivacea* | Microhylidae | Larvae | Laboratory | Pesticides | 4 | 4 | 4 | 175.00 | 20.00 | 162.00 | 26.00 | -0.49 | 0.51 | Widder & Bidwell, 2008 |
| *Gastrophryne olivacea* | Microhylidae | Larvae | Laboratory | Pesticides | 4 | 4 | 4 | 175.00 | 20.00 | 144.00 | 14.00 | -1.56 | 0.65 | Widder & Bidwell, 2008 |
| *Anaxyrus americanus* | Bufonidae | Embryos | Laboratory | Heavy metals | 40 | 20 | 20 | 169.38 | 50.31 | 104.38 | 22.36 | -1.64 | 0.13 | Snodgrass *et al.*, 2008 |
| *Anaxyrus americanus* | Bufonidae | Embryos | Laboratory | Heavy metals | 48 | 20 | 20 | 205.63 | 50.31 | 115.63 | 19.59 | -2.31 | 0.17 | Snodgrass *et al.*, 2008 |
| *Lithobates sphenocephalus* | Ranidae | Larvae | Laboratory | Heavy metals | 254 | 14 | 18 | 1150.00 | 374.17 | 1150.00 | 352.14 | 0.00 | 0.13 | Unrine *et al.*, 2004 |
| *Lithobates sphenocephalus* | Ranidae | Larvae | Laboratory | Heavy metals | 254 | 14 | 11 | 1150.00 | 374.17 | 1040.00 | 139.30 | -0.36 | 0.16 | Unrine *et al.*, 2004 |
| *Lithobates sphenocephalus* | Ranidae | Larvae | Laboratory | Heavy metals | 254 | 14 | 12 | 1150.00 | 374.17 | 1010.00 | 450.33 | -0.33 | 0.16 | Unrine *et al.*, 2004 |
| *Scinax nasicus* | Hylidae | Larvae | Enclosures | Nitrogenous compounds | 7 | 3 | 3 | 460.00 | 40.00 | 410.00 | 50.00 | -0.88 | 0.73 | Peltzer *et al.*, 2008 |
| *Lissotriton vulgaris* | Salamandridae | Larvae | Laboratory | Nitrogenous compounds | 91 | 2 | 6 | 130.00 | 11.31 | 120.00 | 12.25 | -0.72 | 0.70 | Watt & Oldham, 1990 |
| *Hyla chrysoscelis* | Hylidae | Larvae | Laboratory | Pesticides | 12 | 4 | 4 | 79.00 | 16.00 | 83.00 | 14.00 | 0.23 | 0.50 | Widder & Bidwell, 2008 |
| *Hyla chrysoscelis* | Hylidae | Larvae | Laboratory | Pesticides | 12 | 4 | 4 | 79.00 | 16.00 | 91.00 | 4.00 | 0.89 | 0.55 | Widder & Bidwell, 2008 |
| *Hyla chrysoscelis* | Hylidae | Larvae | Laboratory | Pesticides | 12 | 4 | 4 | 79.00 | 16.00 | 99.00 | 28.00 | 0.76 | 0.54 | Widder & Bidwell, 2008 |
| *Hyla chrysoscelis* | Hylidae | Larvae | Laboratory | Pesticides | 12 | 4 | 4 | 79.00 | 16.00 | 83.00 | 6.00 | 0.29 | 0.51 | Widder & Bidwell, 2008 |
| *Lithobates sphenocephalus* | Ranidae | Larvae | Laboratory | Pesticides | 12 | 4 | 4 | 203.00 | 22.00 | 211.00 | 30.00 | 0.26 | 0.50 | Widder & Bidwell, 2008 |
| *Lithobates sphenocephalus* | Ranidae | Larvae | Laboratory | Pesticides | 12 | 4 | 4 | 203.00 | 22.00 | 235.00 | 20.00 | 1.32 | 0.61 | Widder & Bidwell, 2008 |
| *Lithobates sphenocephalus* | Ranidae | Larvae | Laboratory | Pesticides | 12 | 4 | 4 | 203.00 | 22.00 | 170.00 | 28.00 | -1.14 | 0.58 | Widder & Bidwell, 2008 |
| *Lithobates sphenocephalus* | Ranidae | Larvae | Laboratory | Pesticides | 12 | 4 | 4 | 203.00 | 22.00 | 175.00 | 16.00 | -1.27 | 0.60 | Widder & Bidwell, 2008 |
| *Acris crepitans* | Hylidae | Larvae | Laboratory | Pesticides | 4 | 4 | 4 | 189.00 | 10.00 | 173.00 | 16.00 | -1.04 | 0.57 | Widder & Bidwell, 2008 |
| *Acris crepitans* | Hylidae | Larvae | Laboratory | Pesticides | 4 | 4 | 4 | 189.00 | 10.00 | 169.00 | 12.00 | -1.57 | 0.65 | Widder & Bidwell, 2008 |
| *Acris crepitans* | Hylidae | Larvae | Laboratory | Pesticides | 4 | 4 | 4 | 189.00 | 10.00 | 186.00 | 26.00 | -0.13 | 0.50 | Widder & Bidwell, 2008 |
| *Acris crepitans* | Hylidae | Larvae | Laboratory | Pesticides | 4 | 4 | 4 | 189.00 | 10.00 | 198.00 | 10.00 | 0.78 | 0.54 | Widder & Bidwell, 2008 |
| *Gastrophryne olivacea* | Microhylidae | Larvae | Laboratory | Pesticides | 4 | 4 | 4 | 167.00 | 18.00 | 157.00 | 20.00 | -0.46 | 0.51 | Widder & Bidwell, 2008 |
| *Gastrophryne olivacea* | Microhylidae | Larvae | Laboratory | Pesticides | 4 | 4 | 4 | 167.00 | 18.00 | 170.00 | 14.00 | 0.16 | 0.50 | Widder & Bidwell, 2008 |
| *Gastrophryne olivacea* | Microhylidae | Larvae | Laboratory | Pesticides | 4 | 4 | 4 | 167.00 | 18.00 | 157.00 | 14.00 | -0.54 | 0.52 | Widder & Bidwell, 2008 |
| *Gastrophryne olivacea* | Microhylidae | Larvae | Laboratory | Pesticides | 4 | 4 | 4 | 167.00 | 18.00 | 139.00 | 10.00 | -1.67 | 0.67 | Widder & Bidwell, 2008 |
| *Lithobates sphenocephalus* | Ranidae | Larvae | Laboratory | Pesticides | 12 | 4 | 4 | 204.50 | 20.90 | 212.71 | 28.36 | 0.29 | 0.51 | Widder & Bidwell, 2006 |
| *Lithobates sphenocephalus* | Ranidae | Larvae | Laboratory | Pesticides | 12 | 4 | 4 | 204.50 | 20.90 | 237.31 | 19.40 | 1.41 | 0.63 | Widder & Bidwell, 2006 |
| *Lithobates sphenocephalus* | Ranidae | Larvae | Laboratory | Pesticides | 12 | 4 | 4 | 204.50 | 20.90 | 171.64 | 26.86 | -1.19 | 0.59 | Widder & Bidwell, 2006 |
| *Lithobates sphenocephalus* | Ranidae | Larvae | Laboratory | Pesticides | 12 | 4 | 4 | 204.50 | 20.90 | 175.37 | 16.42 | -1.35 | 0.61 | Widder & Bidwell, 2006 |
| *Rana temporaria* | Ranidae | Embryos | Laboratory | Pesticides | 50 | 2 | 2 | 160.68 | 6.80 | 148.81 | 13.13 | -0.65 | 1.05 | Mandrillon & Saglio, 2008 |
| *Rana temporaria* | Ranidae | Embryos | Laboratory | Pesticides | 50 | 2 | 2 | 160.68 | 6.80 | 136.44 | 12.69 | -1.36 | 1.23 | Mandrillon & Saglio, 2008 |
| *Rana temporaria* | Ranidae | Embryos | Laboratory | Pesticides | 50 | 2 | 2 | 160.68 | 6.80 | 178.00 | 8.31 | 1.30 | 1.21 | Mandrillon & Saglio, 2008 |
| *Pelophylax perezi* | Ranidae | Larvae | Laboratory | Nitrogenous compounds | 21 | 7 | 6 | 281.27 | 155.96 | 153.33 | 52.99 | -0.99 | 0.35 | Egea-Serrano *et al*., 2010 |
| *Pelophylax perezi* | Ranidae | Larvae | Laboratory | Nitrogenous compounds | 21 | 7 | 6 | 281.27 | 155.96 | 117.00 | 48.57 | -1.28 | 0.37 | Egea-Serrano *et al*., 2010 |
| *Pelophylax perezi* | Ranidae | Larvae | Laboratory | Nitrogenous compounds | 21 | 7 | 7 | 281.27 | 155.96 | 183.81 | 83.76 | -0.73 | 0.30 | Egea-Serrano *et al*., 2010 |
| *Pelophylax perezi* | Ranidae | Larvae | Laboratory | Nitrogenous compounds | 21 | 7 | 7 | 281.27 | 155.96 | 278.69 | 242.93 | -0.01 | 0.29 | Egea-Serrano *et al*., 2010 |
| *Pelophylax perezi* | Ranidae | Larvae | Laboratory | Nitrogenous compounds | 21 | 7 | 6 | 281.27 | 155.96 | 164.48 | 37.54 | -0.92 | 0.34 | Egea-Serrano *et al*., 2010 |
| *Pelophylax perezi* | Ranidae | Larvae | Laboratory | Nitrogenous compounds | 21 | 7 | 5 | 216.79 | 61.28 | 169.68 | 92.81 | -0.58 | 0.36 | Egea-Serrano *et al*., 2010 |
| *Pelophylax perezi* | Ranidae | Larvae | Laboratory | Nitrogenous compounds | 21 | 7 | 4 | 216.79 | 61.28 | 118.43 | 61.45 | -1.47 | 0.49 | Egea-Serrano *et al*., 2010 |
| *Pelophylax perezi* | Ranidae | Larvae | Laboratory | Nitrogenous compounds | 21 | 7 | 6 | 216.79 | 61.28 | 145.27 | 75.47 | -0.98 | 0.35 | Egea-Serrano *et al*., 2010 |
| *Pelophylax perezi* | Ranidae | Larvae | Laboratory | Nitrogenous compounds | 21 | 7 | 5 | 216.79 | 61.28 | 127.18 | 61.08 | -1.35 | 0.42 | Egea-Serrano *et al*., 2010 |
| *Pelophylax perezi* | Ranidae | Larvae | Laboratory | Nitrogenous compounds | 21 | 7 | 7 | 216.79 | 61.28 | 212.04 | 89.58 | -0.06 | 0.29 | Egea-Serrano *et al*., 2010 |
| *Pelophylax perezi* | Ranidae | Larvae | Laboratory | Nitrogenous compounds | 21 | 7 | 6 | 216.79 | 61.28 | 218.70 | 106.99 | 0.02 | 0.31 | Egea-Serrano *et al*., 2010 |
| *Pelophylax perezi* | Ranidae | Larvae | Laboratory | Nitrogenous compounds | 21 | 5 | 4 | 302.00 | 170.92 | 484.73 | 196.80 | 0.89 | 0.49 | Egea-Serrano *et al*., 2010 |
| *Pelophylax perezi* | Ranidae | Larvae | Laboratory | Nitrogenous compounds | 21 | 5 | 5 | 302.00 | 170.92 | 411.52 | 298.56 | 0.41 | 0.41 | Egea-Serrano *et al*., 2010 |
| *Pelophylax perezi* | Ranidae | Larvae | Laboratory | Nitrogenous compounds | 21 | 5 | 5 | 302.00 | 170.92 | 345.22 | 183.37 | 0.22 | 0.40 | Egea-Serrano *et al*., 2010 |
| *Pelophylax perezi* | Ranidae | Larvae | Laboratory | Nitrogenous compounds | 21 | 5 | 5 | 302.00 | 170.92 | 377.30 | 172.83 | 0.40 | 0.41 | Egea-Serrano *et al*., 2010 |
| *Pelophylax perezi* | Ranidae | Larvae | Laboratory | Nitrogenous compounds | 21 | 5 | 5 | 302.00 | 170.92 | 311.44 | 134.98 | 0.06 | 0.40 | Egea-Serrano *et al*., 2010 |
| *Pelophylax perezi* | Ranidae | Larvae | Laboratory | Nitrogenous compounds | 21 | 5 | 4 | 302.00 | 170.92 | 466.60 | 147.45 | 0.91 | 0.50 | Egea-Serrano *et al*., 2010 |
| *Pelophylax perezi* | Ranidae | Larvae | Laboratory | Nitrogenous compounds | 21 | 5 | 4 | 289.46 | 168.42 | 246.25 | 128.19 | -0.25 | 0.45 | Egea-Serrano *et al*., 2010 |
| *Pelophylax perezi* | Ranidae | Larvae | Laboratory | Nitrogenous compounds | 21 | 5 | 5 | 289.46 | 168.42 | 132.02 | 65.86 | -1.11 | 0.46 | Egea-Serrano *et al*., 2010 |
| *Pelophylax perezi* | Ranidae | Larvae | Laboratory | Nitrogenous compounds | 21 | 5 | 4 | 289.46 | 168.42 | 237.58 | 132.44 | -0.30 | 0.45 | Egea-Serrano *et al*., 2010 |
| *Pelophylax perezi* | Ranidae | Larvae | Laboratory | Nitrogenous compounds | 21 | 5 | 5 | 289.46 | 168.42 | 180.04 | 128.71 | -0.66 | 0.42 | Egea-Serrano *et al*., 2010 |
| *Pelophylax perezi* | Ranidae | Larvae | Laboratory | Nitrogenous compounds | 21 | 5 | 4 | 289.46 | 168.42 | 173.73 | 137.11 | -0.66 | 0.47 | Egea-Serrano *et al*., 2010 |
| *Pelophylax perezi* | Ranidae | Larvae | Laboratory | Nitrogenous compounds | 21 | 5 | 5 | 289.46 | 168.42 | 213.56 | 174.81 | -0.40 | 0.41 | Egea-Serrano *et al*., 2010 |
| *Pelophylax perezi* | Ranidae | Larvae | Mesocosms | Nitrogenous compounds | 21 | 19 | 19 | 90.87 | 17.41 | 74.24 | 26.08 | -0.73 | 0.11 | Egea-Serrano *et al*., 2010 |
| *Pelophylax perezi* | Ranidae | Larvae | Mesocosms | Nitrogenous compounds | 21 | 19 | 19 | 78.06 | 19.00 | 82.91 | 31.90 | 0.18 | 0.11 | Egea-Serrano *et al*., 2010 |
| *Pelophylax perezi* | Ranidae | Larvae | Mesocosms | Nitrogenous compounds | 21 | 20 | 18 | 107.59 | 21.03 | 94.84 | 31.41 | -0.47 | 0.11 | Egea-Serrano *et al*., 2010 |
| *Pelophylax perezi* | Ranidae | Larvae | Enclosures | Nitrogenous compounds | 21 | 8 | 7 | 51.24 | 11.72 | 62.50 | 10.32 | 0.96 | 0.30 | Egea-Serrano *et al*., 2010 |
| *Pelophylax perezi* | Ranidae | Larvae | Enclosures | Nitrogenous compounds | 21 | 8 | 7 | 51.24 | 11.72 | 84.83 | 25.59 | 1.63 | 0.36 | Egea-Serrano *et al*., 2010 |
| *Pelophylax perezi* | Ranidae | Larvae | Enclosures | Nitrogenous compounds | 21 | 8 | 5 | 55.01 | 19.23 | 47.90 | 22.76 | -0.32 | 0.33 | Egea-Serrano *et al*., 2010 |
| *Pelophylax perezi* | Ranidae | Larvae | Enclosures | Nitrogenous compounds | 21 | 8 | 5 | 55.01 | 19.23 | 78.24 | 26.79 | 0.97 | 0.36 | Egea-Serrano *et al*., 2010 |
| *Pelophylax perezi* | Ranidae | Larvae | Enclosures | Nitrogenous compounds | 21 | 7 | 7 | 47.09 | 4.68 | 43.14 | 10.07 | -0.47 | 0.29 | Egea-Serrano *et al*., 2010 |
| *Pelophylax perezi* | Ranidae | Larvae | Enclosures | Nitrogenous compounds | 21 | 7 | 5 | 47.09 | 4.68 | 82.26 | 25.10 | 1.99 | 0.51 | Egea-Serrano *et al*., 2010 |
| *Hyla versicolor* | Hylidae | Larvae | Mesocosms | Pesticides | 78 | 4 | 4 | 647.00 | 93.80 | 572.00 | 93.80 | -0.70 | 0.53 | Relyea, 2008 |
| *Hyla versicolor* | Hylidae | Larvae | Mesocosms | Pesticides | 78 | 4 | 4 | 647.00 | 93.80 | 581.00 | 150.00 | -0.46 | 0.51 | Relyea, 2008 |
| *Hyla versicolor* | Hylidae | Larvae | Mesocosms | Pesticides | 78 | 4 | 4 | 647.00 | 93.80 | 750.00 | 75.00 | 1.05 | 0.57 | Relyea, 2008 |
| *Hyla versicolor* | Hylidae | Larvae | Mesocosms | Pesticides | 78 | 4 | 4 | 647.00 | 93.80 | 581.00 | 112.60 | -0.55 | 0.52 | Relyea, 2008 |
| *Hyla versicolor* | Hylidae | Larvae | Mesocosms | Pesticides | 78 | 4 | 4 | 647.00 | 93.80 | 581.00 | 75.00 | -0.68 | 0.53 | Relyea, 2008 |
| *Hyla versicolor* | Hylidae | Larvae | Mesocosms | Pesticides | 78 | 4 | 4 | 647.00 | 93.80 | 581.00 | 150.00 | -0.46 | 0.51 | Relyea, 2008 |
| *Hyla versicolor* | Hylidae | Larvae | Mesocosms | Pesticides | 78 | 4 | 4 | 647.00 | 93.80 | 563.00 | 75.00 | -0.86 | 0.55 | Relyea, 2008 |
| *Hyla versicolor* | Hylidae | Larvae | Mesocosms | Pesticides | 78 | 4 | 4 | 647.00 | 93.80 | 834.00 | 206.00 | 1.02 | 0.56 | Relyea, 2008 |
| *Lithobates pipiens* | Ranidae | Larvae | Mesocosms | Pesticides | 78 | 4 | 4 | 1906.00 | 250.00 | 1594.00 | 250.00 | -1.09 | 0.57 | Relyea, 2008 |
| *Lithobates pipiens* | Ranidae | Larvae | Mesocosms | Pesticides | 78 | 4 | 4 | 1906.00 | 250.00 | 1344.00 | 250.00 | -1.95 | 0.74 | Relyea, 2008 |
| *Lithobates pipiens* | Ranidae | Larvae | Mesocosms | Pesticides | 78 | 4 | 4 | 1906.00 | 250.00 | 3613.00 | 250.00 | 5.94 | 2.70 | Relyea, 2008 |
| *Lithobates pipiens* | Ranidae | Larvae | Mesocosms | Pesticides | 78 | 4 | 4 | 1906.00 | 250.00 | 1594.00 | 312.00 | -0.96 | 0.56 | Relyea, 2008 |
| *Lithobates pipiens* | Ranidae | Larvae | Mesocosms | Pesticides | 78 | 4 | 4 | 1906.00 | 250.00 | 1688.00 | 312.00 | -0.67 | 0.53 | Relyea, 2008 |
| *Lithobates pipiens* | Ranidae | Larvae | Mesocosms | Pesticides | 78 | 4 | 4 | 1906.00 | 250.00 | 1719.00 | 500.00 | -0.41 | 0.51 | Relyea, 2008 |
| *Lithobates pipiens* | Ranidae | Larvae | Mesocosms | Pesticides | 78 | 4 | 4 | 1906.00 | 250.00 | 1844.00 | 376.00 | -0.17 | 0.50 | Relyea, 2008 |
| *Lithobates pipiens* | Ranidae | Larvae | Mesocosms | Pesticides | 78 | 4 | 4 | 1906.00 | 250.00 | 2125.00 | 376.00 | 0.60 | 0.52 | Relyea, 2008 |

| TIME TO HATCHING (days) | | | | | | | | | | | | | |
| --- | --- | --- | --- | --- | --- | --- | --- | --- | --- | --- | --- | --- | --- |
|  |  |  |  |  | n | | Control treatment | | Polluted treatment | |  |  |  |
| Species | Family | Developmental stage | Experimental venue | Pollutant | Control | Polluted | Mean | SD | Mean | SD | d | Var(d) | Autor |
| *Ambystoma tigrinum* | Ambystomatidae | Embryos | Laboratory | Nitrogenous compounds | 5 | 5 | 5.63 | 0.54 | 6.86 | 0.58 | 1.99 | 0.60 | Griffis-Kyle, 2007 |
| *Lithobates sylvaticus* | Ranidae | Embryos | Laboratory | Nitrogenous compounds | 5 | 5 | 2.60 | 0.76 | 2.40 | 0.49 | -0.28 | 0.40 | Griffis-Kyle, 2007 |
| *Ambystoma barbouri* | Ambystomatidae | Embryos | Laboratory | Pesticides | 4 | 4 | 12.69 | 1.16 | 14.22 | 0.30 | 1.57 | 0.65 | Rohr *et al*., 2003 |
| *Ambystoma barbouri* | Ambystomatidae | Embryos | Laboratory | Pesticides | 4 | 4 | 12.69 | 1.16 | 13.70 | 0.98 | 0.82 | 0.54 | Rohr *et al*., 2003 |
| *Ambystoma barbouri* | Ambystomatidae | Embryos | Laboratory | Pesticides | 4 | 4 | 12.69 | 1.16 | 13.38 | 0.82 | 0.60 | 0.52 | Rohr *et al*., 2003 |
| *Ambystoma barbouri* | Ambystomatidae | Embryos | Laboratory | Pesticides | 4 | 4 | 12.69 | 1.16 | 14.22 | 1.08 | 1.19 | 0.59 | Rohr *et al*., 2003 |
| *Ambystoma tigrinum* | Ambystomatidae | Embryos | Laboratory | Nitrogenous compounds | 5 | 5 | 5.63 | 0.54 | 6.78 | 0.83 | 1.49 | 0.51 | Griffis-Kyle, 2006 |
| *Ambystoma tigrinum* | Ambystomatidae | Embryos | Laboratory | Nitrogenous compounds | 5 | 5 | 5.63 | 0.54 | 7.18 | 0.69 | 2.26 | 0.66 | Griffis-Kyle, 2006 |
| *Ambystoma tigrinum* | Ambystomatidae | Embryos | Laboratory | Nitrogenous compounds | 5 | 5 | 5.63 | 0.54 | 7.16 | 0.89 | 1.87 | 0.58 | Griffis-Kyle, 2006 |
| *Ambystoma tigrinum* | Ambystomatidae | Embryos | Laboratory | Nitrogenous compounds | 5 | 5 | 5.63 | 0.54 | 5.81 | 1.30 | 0.16 | 0.40 | Griffis-Kyle, 2006 |
| *Ambystoma tigrinum* | Ambystomatidae | Embryos | Laboratory | Nitrogenous compounds | 5 | 5 | 5.63 | 0.54 | 5.10 | 1.23 | -0.50 | 0.41 | Griffis-Kyle, 2006 |
| *Lithobates sylvaticus* | Ranidae | Embryos | Laboratory | Nitrogenous compounds | 5 | 5 | 2.60 | 0.76 | 1.96 | 0.87 | -0.71 | 0.42 | Griffis-Kyle, 2006 |
| *Lithobates sylvaticus* | Ranidae | Embryos | Laboratory | Nitrogenous compounds | 5 | 5 | 2.60 | 0.76 | 1.85 | 0.22 | -1.21 | 0.47 | Griffis-Kyle, 2006 |
| *Lithobates sylvaticus* | Ranidae | Embryos | Laboratory | Nitrogenous compounds | 5 | 5 | 2.60 | 0.76 | 2.55 | 0.51 | -0.07 | 0.40 | Griffis-Kyle, 2006 |
| *Lithobates sylvaticus* | Ranidae | Embryos | Laboratory | Nitrogenous compounds | 5 | 5 | 2.60 | 0.76 | 2.23 | 1.41 | -0.30 | 0.40 | Griffis-Kyle, 2006 |
| *Lithobates sylvaticus* | Ranidae | Embryos | Laboratory | Nitrogenous compounds | 5 | 5 | 2.60 | 0.76 | 2.28 | 0.49 | -0.45 | 0.41 | Griffis-Kyle, 2006 |
| *Ambystoma barbouri* | Ambystomatidae | Embryos | Laboratory | Pesticides | 4 | 4 | 12.69 | 1.16 | 13.32 | 0.88 | 0.53 | 0.52 | Rohr *et al*., 2003 |
| *Ambystoma barbouri* | Ambystomatidae | Embryos | Laboratory | Pesticides | 4 | 4 | 12.69 | 1.16 | 12.62 | 1.80 | -0.04 | 0.50 | Rohr *et al*., 2003 |
| *Ambystoma barbouri* | Ambystomatidae | Embryos | Laboratory | Pesticides | 4 | 4 | 12.69 | 1.16 | 13.58 | 0.66 | 0.82 | 0.54 | Rohr *et al*., 2003 |
| *Ambystoma barbouri* | Ambystomatidae | Embryos | Laboratory | Pesticides | 4 | 4 | 12.69 | 1.16 | 13.19 | 0.40 | 0.50 | 0.52 | Rohr *et al*., 2003 |
| *Rana temporaria* | Ranidae | Embryos | Laboratory | Pesticides | 2 | 2 | 8.85 | 0.04 | 8.75 | 0.04 | -1.38 | 1.24 | Mandrillon & Saglio, 2008 |
| *Rana temporaria* | Ranidae | Embryos | Laboratory | Pesticides | 2 | 2 | 8.85 | 0.04 | 8.84 | 0.02 | -0.12 | 1.00 | Mandrillon & Saglio, 2008 |
| *Rana temporaria* | Ranidae | Embryos | Laboratory | Pesticides | 2 | 2 | 8.85 | 0.04 | 8.87 | 0.02 | 0.42 | 1.02 | Mandrillon & Saglio, 2008 |

| TIME TO METAMORPHOSIS (days) | | | | | | | | | | | | | |
| --- | --- | --- | --- | --- | --- | --- | --- | --- | --- | --- | --- | --- | --- |
|  |  |  |  |  | n | | Control treatment | | Polluted treatment | |  |  |  |
| Species | Family | Developmental stage | Experimental venue | Pollutant | Control | Polluted | Mean | SD | Mean | SD | d | Var(d) | Autor |
| *Lissotriton helveticus* | Salamandridae | Larvae | Laboratory | Nitrogenous compounds | 10 | 6 | 65.50 | 27.51 | 42.50 | 14.70 | -0.92 | 0.29 | Watt & Jarvis, 1997 |
| *Lithobates sphenocephalus* | Ranidae | Larvae | Laboratory | Nitrogenous compounds | 15 | 2 | 57.93 | 18.61 | 93.50 | 0.71 | 1.88 | 0.67 | Ortiz-Santaliestra & Sparling, 2007 |
| *Lithobates sphenocephalus* | Ranidae | Larvae | Laboratory | Wastewater contaminants | 15 | 5 | 57.93 | 18.61 | 97.60 | 3.97 | 2.30 | 0.40 | Ortiz-Santaliestra & Sparling, 2007 |
| *Rana temporaria* | Ranidae | Larvae | Mesocosms | Nitrogenous compounds | 3 | 3 | 66.30 | 4.92 | 83.50 | 3.14 | 3.34 | 1.59 | De Wiejer *et al*., 2003 |
| *Rana temporaria* | Ranidae | Larvae | Mesocosms | Nitrogenous compounds | 3 | 3 | 59.70 | 0.33 | 59.40 | 1.37 | -0.24 | 0.67 | De Wiejer *et al*., 2003 |
| *Rana temporaria* | Ranidae | Larvae | Mesocosms | Nitrogenous compounds | 5 | 2 | 59.30 | 0.85 | 61.60 | 1.34 | 2.00 | 0.99 | De Wiejer *et al*., 2003 |
| *Lithobates sylvaticus* | Ranidae | Larvae | Laboratory | Road de-icers | 4 | 4 | 70.52 | 3.26 | 65.19 | 1.78 | -1.76 | 0.69 | Sanzo & Hecnar, 2006 |
| *Lissotriton helveticus* | Salamandridae | Larvae | Laboratory | Nitrogenous compounds | 10 | 6 | 65.40 | 27.51 | 42.50 | 14.70 | -0.91 | 0.29 | Watt & Jarvis, 1997 |
| *Lissotriton helveticus* | Salamandridae | Larvae | Laboratory | Nitrogenous compounds | 10 | 7 | 65.40 | 27.51 | 48.90 | 29.10 | -0.56 | 0.25 | Watt & Jarvis, 1997 |
| *Lissotriton helveticus* | Salamandridae | Larvae | Laboratory | Nitrogenous compounds | 10 | 7 | 65.40 | 27.51 | 38.20 | 15.35 | -1.10 | 0.28 | Watt & Jarvis, 1997 |
| *Lithobates sylvaticus* | Ranidae | Larvae | Laboratory | Road de-icers | 4 | 4 | 71.82 | 2.72 | 69.39 | 3.34 | -0.69 | 0.53 | Sanzo & Hecnar, 2006 |
| *Lithobates sylvaticus* | Ranidae | Larvae | Laboratory | Road de-icers | 4 | 4 | 71.82 | 2.72 | 65.15 | 2.72 | -2.13 | 0.78 | Sanzo & Hecnar, 2006 |
| *Anaxyrus americanus* | Bufonidae | Embryos | Laboratory | Heavy metals | 20 | 20 | 35.05 | 3.09 | 36.83 | 3.09 | 0.57 | 0.10 | Snodgrass *et al*., 2008 |
| *Anaxyrus americanus* | Bufonidae | Embryos | Laboratory | Heavy metals | 20 | 20 | 35.05 | 2.24 | 36.93 | 2.64 | 0.75 | 0.11 | Snodgrass *et al*., 2008 |
| *Lithobates sphenocephalus* | Ranidae | Larvae | Laboratory | Heavy metals | 14 | 18 | 188.33 | 34.31 | 182.50 | 53.03 | -0.12 | 0.13 | Unrine *et al*., 2004 |
| *Lithobates sphenocephalus* | Ranidae | Larvae | Laboratory | Heavy metals | 14 | 11 | 188.33 | 34.31 | 174.17 | 60.79 | -0.29 | 0.16 | Unrine *et al*., 2004 |
| *Lithobates sphenocephalus* | Ranidae | Larvae | Laboratory | Heavy metals | 14 | 12 | 188.33 | 34.31 | 160.83 | 43.30 | -0.69 | 0.16 | Unrine *et al*., 2004 |
| *Lithobates pipiens* | Ranidae | Embryos | Laboratory | Heavy metals | 5 | 5 | 80.29 | 2.27 | 80.87 | 5.51 | 0.12 | 0.40 | Chen *et al*., 2006 |
| *Lithobates pipiens* | Ranidae | Embryos | Laboratory | Heavy metals | 5 | 5 | 80.29 | 2.27 | 82.61 | 6.16 | 0.45 | 0.41 | Chen *et al*., 2006 |
| *Lithobates pipiens* | Ranidae | Embryos | Laboratory | Heavy metals | 5 | 5 | 80.29 | 2.27 | 86.23 | 5.83 | 1.21 | 0.47 | Chen *et al*., 2006 |
| *Lithobates pipiens* | Ranidae | Embryos | Laboratory | Pesticides | 4 | 4 | 109.57 | 7.82 | 110.87 | 3.48 | 0.19 | 0.50 | Chen *et al*., 2007 |
| *Lithobates pipiens* | Ranidae | Embryos | Laboratory | Pesticides | 4 | 4 | 109.57 | 7.82 | 113.48 | 10.44 | 0.37 | 0.51 | Chen *et al*., 2007 |
| *Lithobates pipiens* | Ranidae | Embryos | Laboratory | Pesticides | 4 | 4 | 109.57 | 7.82 | 139.57 | 9.56 | 2.99 | 1.06 | Chen *et al*., 2007 |
| *Hyla versicolor* | Hylidae | Larvae | Mesocosms | Pesticides | 4 | 4 | 25.30 | 1.20 | 25.35 | 0.80 | 0.04 | 0.50 | Relyea, 2008 |
| *Hyla versicolor* | Hylidae | Larvae | Mesocosms | Pesticides | 4 | 4 | 25.30 | 1.20 | 25.60 | 0.80 | 0.26 | 0.50 | Relyea, 2008 |
| *Hyla versicolor* | Hylidae | Larvae | Mesocosms | Pesticides | 4 | 4 | 25.30 | 1.20 | 24.80 | 1.30 | -0.35 | 0.51 | Relyea, 2008 |
| *Hyla versicolor* | Hylidae | Larvae | Mesocosms | Pesticides | 4 | 4 | 25.30 | 1.20 | 24.30 | 0.90 | -0.82 | 0.54 | Relyea, 2008 |
| *Hyla versicolor* | Hylidae | Larvae | Mesocosms | Pesticides | 4 | 4 | 25.30 | 1.20 | 24.90 | 0.60 | -0.37 | 0.51 | Relyea, 2008 |
| *Hyla versicolor* | Hylidae | Larvae | Mesocosms | Pesticides | 4 | 4 | 25.30 | 1.20 | 26.20 | 1.00 | 0.71 | 0.53 | Relyea, 2008 |
| *Hyla versicolor* | Hylidae | Larvae | Mesocosms | Pesticides | 4 | 4 | 25.30 | 1.20 | 26.45 | 1.20 | 0.83 | 0.54 | Relyea, 2008 |
| *Hyla versicolor* | Hylidae | Larvae | Mesocosms | Pesticides | 4 | 4 | 25.30 | 1.20 | 24.50 | 0.60 | -0.73 | 0.53 | Relyea, 2008 |
| *Lithobates pipiens* | Ranidae | Larvae | Mesocosms | Pesticides | 4 | 4 | 41.41 | 2.81 | 42.19 | 3.13 | 0.23 | 0.50 | Relyea, 2008 |
| *Lithobates pipiens* | Ranidae | Larvae | Mesocosms | Pesticides | 4 | 4 | 41.41 | 2.81 | 43.60 | 4.69 | 0.49 | 0.52 | Relyea, 2008 |
| *Lithobates pipiens* | Ranidae | Larvae | Mesocosms | Pesticides | 4 | 4 | 41.41 | 2.81 | 45.16 | 4.69 | 0.84 | 0.54 | Relyea, 2008 |
| *Lithobates pipiens* | Ranidae | Larvae | Mesocosms | Pesticides | 4 | 4 | 41.41 | 2.81 | 44.22 | 2.19 | 0.97 | 0.56 | Relyea, 2008 |
| *Lithobates pipiens* | Ranidae | Larvae | Mesocosms | Pesticides | 4 | 4 | 41.41 | 2.81 | 43.29 | 3.13 | 0.55 | 0.52 | Relyea, 2008 |
| *Lithobates pipiens* | Ranidae | Larvae | Mesocosms | Pesticides | 4 | 4 | 41.41 | 2.81 | 40.78 | 1.88 | -0.23 | 0.50 | Relyea, 2008 |

|  |  | Proportion of individuals showing abnormalities | | | | | | | | | | | | |
| --- | --- | --- | --- | --- | --- | --- | --- | --- | --- | --- | --- | --- | --- | --- |
|  |  |  |  |  |  | n | | Control treatment | | Polluted treatment | |  |  |  |
| Species | Family | Developmental stage | Experimental venue | Pollutant | Days of exposure | Control | Polluted | Mean | SD | Mean | SD | d | Var(d) | Reference |
| *Lithobates sylvaticus* | Ranidae | Embryos | Laboratory | Nitrogenous compounds | 23 | 5 | 5 | 0.11 | 0.06 | 0.38 | 0.16 | 2.03 | 0.61 | Laposata & Dunson, 1998 |
| *Ambystoma jeffersonianum* | Ambystomatidae | Embryos | Laboratory | Nitrogenous compounds | 25 | 5 | 5 | 0.03 | 0.06 | 0.09 | 0.09 | 0.74 | 0.43 | Laposata & Dunson, 1998 |
| *Ambystoma maculatum* | Ambystomatidae | Embryos | Laboratory | Nitrogenous compounds | 44 | 5 | 5 | 0.00 | 0.00 | 0.04 | 0.04 | 1.36 | 0.49 | Laposata & Dunson, 1998 |
| *Pleurodeles waltl* | Salamandridae | Embryos | Laboratory | Nitrogenous compounds | 15 | 3 | 3 | 0.03 | 0.05 | 0.00 | 0.00 | -0.66 | 0.70 | Ortiz *et al.*, 2004 |
| *Discoglossus galganoi* | Alytidae | Embryos | Laboratory | Nitrogenous compounds | 15 | 3 | 3 | 0.02 | 0.04 | 0.33 | 0.07 | 4.57 | 2.41 | Ortiz *et al.*, 2004 |
| *Bufo bufo* | Bufonidae | Embryos | Laboratory | Nitrogenous compounds | 15 | 9 | 9 | 0.08 | 0.07 | 0.30 | 0.12 | 2.13 | 0.35 | Ortiz *et al.*, 2004 |
| *Epidalea calamita* | Bufonidae | Embryos | Laboratory | Nitrogenous compounds | 15 | 9 | 9 | 0.02 | 0.03 | 0.06 | 0.04 | 1.21 | 0.26 | Ortiz *et al.*, 2004 |
| *Hyla arborea* | Hylidae | Embryos | Laboratory | Nitrogenous compounds | 15 | 3 | 3 | 0.03 | 0.06 | 0.02 | 0.03 | -0.28 | 0.67 | Ortiz *et al.*, 2004 |
| *Xenopus laevis* | Pipidae | Larvae | Laboratory | Pesticides | 78 | 11 | 11 | 0.01 | 0.01 | 0.02 | 0.02 | 0.78 | 0.20 | Carr *et al.*, 2003 |
| *Lithobates sylvaticus* | Ranidae | Embryos | Laboratory | Wastewater contaminants | 23 | 5 | 5 | 0.01 | 0.03 | 0.78 | 0.10 | 9.35 | 4.77 | Laposata & Dunson, 1998 |
| *Ambystoma jeffersonianum* | Ambystomatidae | Embryos | Laboratory | Wastewater contaminants | 25 | 5 | 5 | 0.00 | 0.00 | 0.13 | 0.08 | 2.13 | 0.63 | Laposata & Dunson, 1998 |
| *Ambystoma maculatum* | Ambystomatidae | Embryos | Laboratory | Wastewater contaminants | 44 | 5 | 5 | 0.00 | 0.00 | 0.80 | 0.12 | 8.51 | 4.02 | Laposata & Dunson, 1998 |
| *Lithobates sylvaticus* | Ranidae | Embryos | Laboratory | Nitrogenous compounds | 23 | 5 | 5 | 0.11 | 0.06 | 0.20 | 0.08 | 1.12 | 0.46 | Laposata & Dunson, 1998 |
| *Ambystoma jeffersonianum* | Ambystomatidae | Embryos | Laboratory | Nitrogenous compounds | 24 | 5 | 5 | 0.03 | 0.06 | 0.03 | 0.04 | -0.02 | 0.40 | Laposata & Dunson, 1998 |
| *Ambystoma maculatum* | Ambystomatidae | Embryos | Laboratory | Nitrogenous compounds | 44 | 5 | 5 | 0.00 | 0.00 | 0.03 | 0.04 | 0.91 | 0.44 | Laposata & Dunson, 1998 |
| *Lithobates sylvaticus* | Ranidae | Embryos | Laboratory | Nitrogenous compounds | 23 | 5 | 5 | 0.11 | 0.06 | 0.21 | 0.17 | 0.75 | 0.43 | Laposata & Dunson, 1998 |
| *Ambystoma jeffersonianum* | Ambystomatidae | Embryos | Laboratory | Nitrogenous compounds | 25 | 5 | 5 | 0.03 | 0.06 | 0.07 | 0.07 | 0.54 | 0.41 | Laposata & Dunson, 1998 |
| *Pleurodeles waltl* | Salamandridae | Embryos | Laboratory | Nitrogenous compounds | 15 | 3 | 3 | 0.03 | 0.05 | 0.00 | 0.00 | -0.66 | 0.70 | Ortiz *et al.*, 2004 |
| *Discoglossus galganoi* | Alytidae | Embryos | Laboratory | Nitrogenous compounds | 15 | 3 | 3 | 0.02 | 0.04 | 0.04 | 0.04 | 0.46 | 0.68 | Ortiz *et al.*, 2004 |
| *Bufo bufo* | Bufonidae | Embryos | Laboratory | Nitrogenous compounds | 15 | 9 | 9 | 0.08 | 0.07 | 0.14 | 0.05 | 0.97 | 0.25 | Ortiz *et al.*, 2004 |
| *Epidalea calamita* | Bufonidae | Embryos | Laboratory | Nitrogenous compounds | 15 | 9 | 9 | 0.02 | 0.03 | 0.04 | 0.03 | 0.72 | 0.24 | Ortiz *et al.*, 2004 |
| *Hyla arborea* | Hylidae | Embryos | Laboratory | Nitrogenous compounds | 15 | 3 | 3 | 0.03 | 0.06 | 0.07 | 0.08 | 0.40 | 0.68 | Ortiz *et al.*, 2004 |
| *Xenopus laevis* | Pipidae | Larvae | Laboratory | Pesticides | 78 | 11 | 11 | 0.01 | 0.01 | 0.00 | 0.01 | -0.33 | 0.18 | Carr *et al.*, 2003 |
| *Xenopus laevis* | Pipidae | Larvae | Laboratory | Pesticides | 78 | 11 | 11 | 0.01 | 0.01 | 0.01 | 0.01 | 0.21 | 0.18 | Carr *et al.*, 2003 |
| *Pleurodeles waltl* | Salamandridae | Embryos | Laboratory | Nitrogenous compounds | 15 | 3 | 3 | 0.03 | 0.05 | 0.00 | 0.00 | -0.66 | 0.70 | Ortiz *et al.*, 2004 |
| *Discoglossus galganoi* | Alytidae | Embryos | Laboratory | Nitrogenous compounds | 15 | 3 | 3 | 0.02 | 0.04 | 0.07 | 0.12 | 0.42 | 0.68 | Ortiz *et al.*, 2004 |
| *Hyla arborea* | Hylidae | Embryos | Laboratory | Nitrogenous compounds | 15 | 3 | 3 | 0.03 | 0.06 | 0.02 | 0.03 | -0.28 | 0.67 | Ortiz *et al.*, 2004 |
| *Lithobates sylvaticus* | Ranidae | Embryos | Laboratory | Wastewater contaminants | 23 | 5 | 5 | 0.01 | 0.03 | 0.52 | 0.14 | 4.52 | 1.42 | Laposata & Dunson, 1998 |
| *Ambystoma jeffersonianum* | Ambystomatidae | Embryos | Laboratory | Wastewater contaminants | 25 | 5 | 5 | 0.00 | 0.00 | 0.07 | 0.05 | 1.82 | 0.57 | Laposata & Dunson, 1998 |
| *Ambystoma maculatum* | Ambystomatidae | Embryos | Laboratory | Wastewater contaminants | 44 | 5 | 5 | 0.00 | 0.00 | 0.18 | 0.10 | 2.26 | 0.66 | Laposata & Dunson, 1998 |
| *Lithobates pipiens* | Ranidae | Embryos | Laboratory | Pesticides | 154 | 5 | 5 | 0.00 | 0.00 | 0.02 | 0.03 | 0.73 | 0.43 | Chen *et al.*, 2007 |
| *Lithobates pipiens* | Ranidae | Embryos | Laboratory | Pesticides | 154 | 5 | 5 | 0.00 | 0.00 | 0.01 | 0.02 | 0.90 | 0.44 | Chen *et al.*, 2007 |
| *Lithobates pipiens* | Ranidae | Embryos | Laboratory | Pesticides | 154 | 5 | 5 | 0.00 | 0.00 | 0.06 | 0.04 | 1.64 | 0.53 | Chen *et al.*, 2007 |

| Proportion of surviving tadpoles (factorial meta-analysis) | | | | | | | | | | | | | | | | | | |
| --- | --- | --- | --- | --- | --- | --- | --- | --- | --- | --- | --- | --- | --- | --- | --- | --- | --- | --- |
| Species | Family | Developmental stage | Experimental venue | Days of exposure | STRESS-1 (factor) | STREES-2 (factor) | STRESS 1 (category) | STRESS 2 (category) | n | Mean | SD | STRESS-1 | Var (STRESS-1) | STRESS-2 | Var (STRESS-2) | STRESS-1 x  STRESS-2 | Var (STRESS-1 x  STRESS-2) | Reference |
| *Lithobates catesbeianus* | Ranidae | Larvae | Mesocosms | 42 | Nitrogenous compounds | Competitors | No pollutant | No competitors | 3 | 0.96 | 0.02 | -1.13 | 0.35 | -0.62 | 0.34 | -1.24 | 1.40 | Smith *et al.*, 2006 |
| *Lithobates catesbeianus* | Ranidae | Larvae | Mesocosms | 42 |  |  | No pollutant | Competitors | 3 | 0.96 | 0.02 |  |  |  |  |  |  | Smith *et al.*, 2006 |
| *Lithobates catesbeianus* | Ranidae | Larvae | Mesocosms | 42 |  |  | Pollutant | No competitors | 3 | 0.79 | 0.14 |  |  |  |  |  |  | Smith *et al.*, 2006 |
| *Lithobates catesbeianus* | Ranidae | Larvae | Mesocosms | 42 |  |  | Pollutant | Competitors | 3 | 0.38 | 0.40 |  |  |  |  |  |  | Smith *et al.*, 2006 |
| *Lithobates clamitans* | Ranidae | Larvae | Mesocosms | 42 | Nitrogenous compounds | Competitors | No pollutant | No competitors | 3 | 0.52 | 0.28 | 0.21 | 0.33 | -0.55 | 0.34 | -1.10 | 1.38 | Smith *et al.*, 2006 |
| *Lithobates clamitans* | Ranidae | Larvae | Mesocosms | 42 |  |  | No pollutant | Competitors | 3 | 0.52 | 0.28 |  |  |  |  |  |  | Smith *et al.*, 2006 |
| *Lithobates clamitans* | Ranidae | Larvae | Mesocosms | 42 |  |  | Pollutant | No competitors | 3 | 0.86 | 0.11 |  |  |  |  |  |  | Smith *et al.*, 2006 |
| *Lithobates clamitans* | Ranidae | Larvae | Mesocosms | 42 |  |  | Pollutant | Competitors | 3 | 0.37 | 0.40 |  |  |  |  |  |  | Smith *et al.*, 2006 |
| *Ambystoma maculatum* | Ambystomatidae | Larvae | Mesocosms | 137 | Pesticides | Competitors | No pollutant | Low density | 4 | 0.84 | 0.13 | -0.04 | 0.25 | -1.07 | 0.26 | 0.36 | 1.00 | Metts *et al.*, 2005 |
| *Ambystoma maculatum* | Ambystomatidae | Larvae | Mesocosms | 137 |  |  | No pollutant | High density | 4 | 0.59 | 0.10 |  |  |  |  |  |  | Metts *et al.*, 2005 |
| *Ambystoma maculatum* | Ambystomatidae | Larvae | Mesocosms | 137 |  |  | Low pollutant | Low density | 4 | 0.80 | 0.26 |  |  |  |  |  |  | Metts *et al.*, 2005 |
| *Ambystoma maculatum* | Ambystomatidae | Larvae | Mesocosms | 137 |  |  | Low pollutant | High density | 4 | 0.62 | 0.12 |  |  |  |  |  |  | Metts *et al.*, 2005 |
| *Ambystoma maculatum* | Ambystomatidae | Larvae | Mesocosms | 137 | Pesticides | Competitors | No pollutant | Low density | 4 | 0.84 | 0.13 | -5.83 | 0.52 | -0.94 | 0.26 | 2.51 | 1.20 | Metts *et al.*, 2005 |
| *Ambystoma maculatum* | Ambystomatidae | Larvae | Mesocosms | 137 |  |  | No pollutant | High density | 4 | 0.59 | 0.10 |  |  |  |  |  |  | Metts *et al.*, 2005 |
| *Ambystoma maculatum* | Ambystomatidae | Larvae | Mesocosms | 137 |  |  | High pollutant | Low density | 4 | 0.04 | 0.09 |  |  |  |  |  |  | Metts *et al.*, 2005 |
| *Ambystoma maculatum* | Ambystomatidae | Larvae | Mesocosms | 137 |  |  | High pollutant | High density | 4 | 0.07 | 0.01 |  |  |  |  |  |  | Metts *et al.*, 2005 |
| *Ambystoma opacum* | Ambystomatidae | Larvae | Mesocosms | 137 | Pesticides | Competitors | No pollutant | Low density | 4 | 0.96 | 0.09 | -13.08 | 1.59 | -1.39 | 0.27 | -3.01 | 1.28 | Metts *et al.*, 2005 |
| *Ambystoma opacum* | Ambystomatidae | Larvae | Mesocosms | 137 |  |  | No pollutant | High density | 4 | 0.96 | 0.04 |  |  |  |  |  |  | Metts *et al.*, 2005 |
| *Ambystoma opacum* | Ambystomatidae | Larvae | Mesocosms | 137 |  |  | Low pollutant | Low density | 4 | 0.21 | 0.03 |  |  |  |  |  |  | Metts *et al.*, 2005 |
| *Ambystoma opacum* | Ambystomatidae | Larvae | Mesocosms | 137 |  |  | Low pollutant | High density | 4 | 0.03 | 0.03 |  |  |  |  |  |  | Metts *et al.*, 2005 |
| *Lithobates catesbeianus* | Ranidae | Larvae | Mesocosms | 31 | Pesticides | pH | No pollutant | pH=8 | 4 | 0.68 | 0.08 | 0.05 | 0.25 | 0.64 | 0.25 | 0.18 | 1.00 | Relyea, 2006 |
| *Lithobates catesbeianus* | Ranidae | Larvae | Mesocosms | 31 |  |  | No pollutant | pH=6 | 4 | 0.76 | 0.16 |  |  |  |  |  |  | Relyea, 2006 |
| *Lithobates catesbeianus* | Ranidae | Larvae | Mesocosms | 31 |  |  | Low pollutant | pH=8 | 4 | 0.68 | 0.08 |  |  |  |  |  |  | Relyea, 2006 |
| *Lithobates catesbeianus* | Ranidae | Larvae | Mesocosms | 31 |  |  | Low pollutant | pH=6 | 4 | 0.78 | 0.10 |  |  |  |  |  |  | Relyea, 2006 |
| *Lithobates catesbeianus* | Ranidae | Larvae | Mesocosms | 31 | Pesticides | pH | No pollutant | pH=8 | 4 | 0.68 | 0.08 | -0.69 | 0.25 | 0.34 | 0.25 | -0.21 | 1.00 | Relyea, 2006 |
| *Lithobates catesbeianus* | Ranidae | Larvae | Mesocosms | 31 |  |  | No pollutant | pH=6 | 4 | 0.76 | 0.16 |  |  |  |  |  |  | Relyea, 2006 |
| *Lithobates catesbeianus* | Ranidae | Larvae | Mesocosms | 31 |  |  | High pollutant | pH=8 | 4 | 0.58 | 0.20 |  |  |  |  |  |  | Relyea, 2006 |
| *Lithobates catesbeianus* | Ranidae | Larvae | Mesocosms | 31 |  |  | High pollutant | pH=6 | 4 | 0.62 | 0.09 |  |  |  |  |  |  | Relyea, 2006 |
| *Lithobates catesbeianus* | Ranidae | Larvae | Mesocosms | 31 | Pesticides | Predators | No pollutant | No predator | 4 | 0.68 | 0.08 | 0.09 | 0.25 | -0.16 | 0.25 | 0.26 | 1.00 | Relyea, 2006 |
| *Lithobates catesbeianus* | Ranidae | Larvae | Mesocosms | 31 |  |  | No pollutant | Predator | 4 | 0.64 | 0.17 |  |  |  |  |  |  | Relyea, 2006 |
| *Lithobates catesbeianus* | Ranidae | Larvae | Mesocosms | 31 |  |  | Low pollutant | No predator | 4 | 0.68 | 0.08 |  |  |  |  |  |  | Relyea, 2006 |
| *Lithobates catesbeianus* | Ranidae | Larvae | Mesocosms | 31 |  |  | Low pollutant | Predator | 4 | 0.67 | 0.09 |  |  |  |  |  |  | Relyea, 2006 |
| *Lithobates catesbeianus* | Ranidae | Larvae | Mesocosms | 31 | Pesticides | Predators | No pollutant | No predator | 4 | 0.68 | 0.08 | -0.44 | 0.25 | -0.09 | 0.25 | 0.29 | 1.00 | Relyea, 2006 |
| *Lithobates catesbeianus* | Ranidae | Larvae | Mesocosms | 31 |  |  | No pollutant | Predator | 4 | 0.64 | 0.17 |  |  |  |  |  |  | Relyea, 2006 |
| *Lithobates catesbeianus* | Ranidae | Larvae | Mesocosms | 31 |  |  | High pollutant | No predator | 4 | 0.58 | 0.20 |  |  |  |  |  |  | Relyea, 2006 |
| *Lithobates catesbeianus* | Ranidae | Larvae | Mesocosms | 31 |  |  | High pollutant | Predator | 4 | 0.59 | 0.07 |  |  |  |  |  |  | Relyea, 2006 |
| *Lithobates clamitans* | Ranidae | Larvae | Mesocosms | 31 | Pesticides | pH | No pollutant | pH=8 | 4 | 0.61 | 0.27 | 0.23 | 0.25 | 0.41 | 0.25 | 0.33 | 1.00 | Relyea, 2006 |
| *Lithobates clamitans* | Ranidae | Larvae | Mesocosms | 31 |  |  | No pollutant | pH=6 | 4 | 0.70 | 0.47 |  |  |  |  |  |  | Relyea, 2006 |
| *Lithobates clamitans* | Ranidae | Larvae | Mesocosms | 31 |  |  | Low pollutant | pH=8 | 4 | 0.64 | 0.26 |  |  |  |  |  |  | Relyea, 2006 |
| *Lithobates clamitans* | Ranidae | Larvae | Mesocosms | 31 |  |  | Low pollutant | pH=6 | 4 | 0.85 | 0.07 |  |  |  |  |  |  | Relyea, 2006 |
| *Lithobates clamitans* | Ranidae | Larvae | Mesocosms | 31 | Pesticides | pH | No pollutant | pH=8 | 4 | 0.61 | 0.27 | 0.12 | 0.25 | 0.02 | 0.25 | -0.47 | 1.01 | Relyea, 2006 |
| *Lithobates clamitans* | Ranidae | Larvae | Mesocosms | 31 |  |  | No pollutant | pH=6 | 4 | 0.70 | 0.47 |  |  |  |  |  |  | Relyea, 2006 |
| *Lithobates clamitans* | Ranidae | Larvae | Mesocosms | 31 |  |  | High pollutant | pH=8 | 4 | 0.74 | 0.12 |  |  |  |  |  |  | Relyea, 2006 |
| *Lithobates clamitans* | Ranidae | Larvae | Mesocosms | 31 |  |  | High pollutant | pH=6 | 4 | 0.66 | 0.19 |  |  |  |  |  |  | Relyea, 2006 |
| *Lithobates clamitans* | Ranidae | Larvae | Mesocosms | 31 | Pesticides | Predators | No pollutant | No predator | 4 | 0.61 | 0.27 | -0.59 | 0.25 | -0.08 | 0.25 | -1.34 | 1.06 | Relyea, 2006 |
| *Lithobates clamitans* | Ranidae | Larvae | Mesocosms | 31 |  |  | No pollutant | Predator | 4 | 0.79 | 0.08 |  |  |  |  |  |  | Relyea, 2006 |
| *Lithobates clamitans* | Ranidae | Larvae | Mesocosms | 31 |  |  | Low pollutant | No predator | 4 | 0.64 | 0.26 |  |  |  |  |  |  | Relyea, 2006 |
| *Lithobates clamitans* | Ranidae | Larvae | Mesocosms | 31 |  |  | Low pollutant | Predator | 4 | 0.41 | 0.32 |  |  |  |  |  |  | Relyea, 2006 |
| *Lithobates clamitans* | Ranidae | Larvae | Mesocosms | 31 | Pesticides | Predators | No pollutant | No predator | 4 | 0.61 | 0.27 | 0.17 | 0.25 | 0.41 | 0.25 | -0.86 | 1.02 | Relyea, 2006 |
| *Lithobates clamitans* | Ranidae | Larvae | Mesocosms | 31 |  |  | No pollutant | Predator | 4 | 0.79 | 0.08 |  |  |  |  |  |  | Relyea, 2006 |
| *Lithobates clamitans* | Ranidae | Larvae | Mesocosms | 31 |  |  | High pollutant | No predator | 4 | 0.74 | 0.12 |  |  |  |  |  |  | Relyea, 2006 |
| *Lithobates clamitans* | Ranidae | Larvae | Mesocosms | 31 |  |  | High pollutant | Predator | 4 | 0.73 | 0.16 |  |  |  |  |  |  | Relyea, 2006 |
| *Hyla versicolor* | Hylidae | Larvae | Laboratory | 10 | Pesticides | Predators | No pollutant | No predator | 4 | 0.96 | 0.04 | -2.17 | 0.29 | -1.79 | 0.27 | 0.36 | 1.00 | Relyea & Mills, 2001 |
| *Hyla versicolor* | Hylidae | Larvae | Laboratory | 16 |  |  | No pollutant | Predator | 4 | 0.79 | 0.33 |  |  |  |  |  |  | Relyea & Mills, 2001 |
| *Hyla versicolor* | Hylidae | Larvae | Mesocosms | 10 |  |  | very low pollutant | No predator | 4 | 0.40 | 0.31 |  |  |  |  |  |  | Relyea & Mills, 2001 |
| *Hyla versicolor* | Hylidae | Larvae | Laboratory | 10 |  |  | very low pollutant | Predator | 4 | 0.03 | 0.05 |  |  |  |  |  |  | Relyea & Mills, 2001 |
| *Hyla versicolor* | Hylidae | Larvae | Laboratory | 10 | Pesticides | Predators | No pollutant | No predator | 4 | 0.96 | 0.04 | -0.74 | 0.25 | -3.00 | 0.32 | -1.01 | 1.03 | Relyea & Mills, 2001 |
| *Hyla versicolor* | Hylidae | Larvae | Laboratory | 16 |  |  | No pollutant | Predator | 4 | 0.79 | 0.33 |  |  |  |  |  |  | Relyea & Mills, 2001 |
| *Hyla versicolor* | Hylidae | Larvae | Laboratory | 10 |  |  | Low pollutant | No predator | 4 | 0.91 | 0.18 |  |  |  |  |  |  | Relyea & Mills, 2001 |
| *Hyla versicolor* | Hylidae | Larvae | Laboratory | 16 |  |  | Low pollutant | Predator | 4 | 0.42 | 0.19 |  |  |  |  |  |  | Relyea & Mills, 2001 |
| *Hyla versicolor* | Hylidae | Larvae | Laboratory | 10 | Pesticides | Predators | No pollutant | No predator | 4 | 0.96 | 0.04 | -1.76 | 0.27 | -1.47 | 0.27 | 0.93 | 1.03 | Relyea & Mills, 2001 |
| *Hyla versicolor* | Hylidae | Larvae | Laboratory | 16 |  |  | No pollutant | Predator | 4 | 0.79 | 0.33 |  |  |  |  |  |  | Relyea & Mills, 2001 |
| *Hyla versicolor* | Hylidae | Larvae | Laboratory | 16 |  |  | medium pollutant | No predator | 4 | 0.68 | 0.43 |  |  |  |  |  |  | Relyea & Mills, 2001 |
| *Hyla versicolor* | Hylidae | Larvae | Laboratory | 16 |  |  | medium pollutant | Predator | 4 | 0.29 | 0.29 |  |  |  |  |  |  | Relyea & Mills, 2001 |
| *Hyla versicolor* | Hylidae | Larvae | Laboratory | 10 | Pesticides | Predators | No pollutant | No predator | 4 | 0.96 | 0.04 | -2.15 | 0.29 | -2.11 | 0.28 | 0.97 | 1.03 | Relyea & Mills, 2001 |
| *Hyla versicolor* | Hylidae | Larvae | Laboratory | 16 |  |  | No pollutant | Predator | 4 | 0.79 | 0.33 |  |  |  |  |  |  | Relyea & Mills, 2001 |
| *Hyla versicolor* | Hylidae | Larvae | Laboratory | 16 |  |  | High pollutant | No predator | 4 | 0.78 | 0.24 |  |  |  |  |  |  | Relyea & Mills, 2001 |
| *Hyla versicolor* | Hylidae | Larvae | Laboratory | 16 |  |  | High pollutant | Predator | 4 | 0.32 | 0.21 |  |  |  |  |  |  | Relyea & Mills, 2001 |
| *Hyla versicolor* | Hylidae | Larvae | Laboratory | 10 | Pesticides | Predators | No pollutant | No predator | 4 | 0.96 | 0.04 | -1.98 | 0.28 | -2.84 | 0.31 | 0.35 | 1.00 | Relyea & Mills, 2001 |
| *Hyla versicolor* | Hylidae | Larvae | Laboratory | 16 |  |  | No pollutant | Predator | 4 | 0.79 | 0.33 |  |  |  |  |  |  | Relyea & Mills, 2001 |
| *Hyla versicolor* | Hylidae | Larvae | Laboratory | 16 |  |  | very high pollutant | No predator | 4 | 1.00 | 0.00 |  |  |  |  |  |  | Relyea & Mills, 2001 |
| *Hyla versicolor* | Hylidae | Larvae | Laboratory | 16 |  |  | very high pollutant | Predator | 4 | 0.35 | 0.21 |  |  |  |  |  |  | Relyea & Mills, 2001 |
| *Lithobates sphenocephalus* | Ranidae | Larvae | Laboratory | 12 | Pesticides | Predators | No pollutant | No predator | 4 | 0.92 | 0.04 | 1.67 | 0.27 | -1.93 | 0.28 | 3.47 | 1.38 | Widder & Bidwell, 2006 |
| *Lithobates sphenocephalus* | Ranidae | Larvae | Laboratory | 12 |  |  | No pollutant | Predator | 4 | 0.36 | 0.24 |  |  |  |  |  |  | Widder & Bidwell, 2006 |
| *Lithobates sphenocephalus* | Ranidae | Larvae | Laboratory | 12 |  |  | Pollutant | No predator | 4 | 0.91 | 0.04 |  |  |  |  |  |  | Widder & Bidwell, 2006 |
| *Lithobates sphenocephalus* | Ranidae | Larvae | Laboratory | 12 |  |  | Pollutant | Predator | 4 | 0.88 | 0.02 |  |  |  |  |  |  | Widder & Bidwell, 2006 |
| *Lithobates catesbeianus* | Ranidae | Larvae | Mesocosms | 69 | Pesticides | Predators | No pollutant | No predator | 3 | 0.64 | 0.21 | 0.53 | 0.34 | 0.38 | 0.33 | -0.53 | 1.35 | Boone & Semlitsch, 2003 |
| *Lithobates catesbeianus* | Ranidae | Larvae | Mesocosms | 69 |  |  | No pollutant | Predator | 3 | 0.86 | 0.23 |  |  |  |  |  |  | Boone & Semlitsch, 2003 |
| *Lithobates catesbeianus* | Ranidae | Larvae | Mesocosms | 69 |  |  | Pollutant | No predator | 3 | 0.91 | 0.21 |  |  |  |  |  |  | Boone & Semlitsch, 2003 |
| *Lithobates catesbeianus* | Ranidae | Larvae | Mesocosms | 69 |  |  | Pollutant | Predator | 3 | 0.95 | 0.23 |  |  |  |  |  |  | Boone & Semlitsch, 2003 |
| *Lithobates catesbeianus* | Ranidae | Larvae | Mesocosms | 69 | Pesticides | Predators | No pollutant | No predator | 3 | 0.64 | 0.21 | 0.51 | 0.34 | -0.81 | 0.34 | -0.60 | 1.35 | Boone & Semlitsch, 2003 |
| *Lithobates catesbeianus* | Ranidae | Larvae | Mesocosms | 69 |  |  | No pollutant | Predator | 3 | 0.47 | 0.21 |  |  |  |  |  |  | Boone & Semlitsch, 2003 |
| *Lithobates catesbeianus* | Ranidae | Larvae | Mesocosms | 69 |  |  | Pollutant | No predator | 3 | 0.91 | 0.21 |  |  |  |  |  |  | Boone & Semlitsch, 2003 |
| *Lithobates catesbeianus* | Ranidae | Larvae | Mesocosms | 69 |  |  | Pollutant | Predator | 3 | 0.54 | 0.23 |  |  |  |  |  |  | Boone & Semlitsch, 2003 |
| *Lithobates catesbeianus* | Ranidae | Larvae | Mesocosms | 69 | Pesticides | Predators | No pollutant | No predator | 3 | 0.64 | 0.21 | 1.20 | 0.35 | -0.40 | 0.33 | 0.74 | 1.36 | Boone & Semlitsch, 2003 |
| *Lithobates catesbeianus* | Ranidae | Larvae | Mesocosms | 69 |  |  | No pollutant | Predator | 3 | 0.39 | 0.21 |  |  |  |  |  |  | Boone & Semlitsch, 2003 |
| *Lithobates catesbeianus* | Ranidae | Larvae | Mesocosms | 69 |  |  | Pollutant | No predator | 3 | 0.91 | 0.21 |  |  |  |  |  |  | Boone & Semlitsch, 2003 |
| *Lithobates catesbeianus* | Ranidae | Larvae | Mesocosms | 69 |  |  | Pollutant | Predator | 3 | 0.90 | 0.21 |  |  |  |  |  |  | Boone & Semlitsch, 2003 |
| *Rana cascadae* | Ranidae | Larvae | Laboratory | 21 | Nitrogenous compounds | pH | No pollutant | pH=7 | 4 | 0.99 | 0.00 | -0.41 | 0.25 | -0.41 | 0.25 | -1.14 | 1.04 | Hatch & Blaustein, 2000 |
| *Rana cascadae* | Ranidae | Larvae | Laboratory | 21 |  |  | No pollutant | pH=5 | 4 | 1.00 | 0.00 |  |  |  |  |  |  | Hatch & Blaustein, 2000 |
| *Rana cascadae* | Ranidae | Larvae | Laboratory | 21 |  |  | Low pollutant | pH=7 | 4 | 1.00 | 0.00 |  |  |  |  |  |  | Hatch & Blaustein, 2000 |
| *Rana cascadae* | Ranidae | Larvae | Laboratory | 21 |  |  | Low pollutant | pH=5 | 4 | 0.94 | 0.10 |  |  |  |  |  |  | Hatch & Blaustein, 2000 |
| *Rana cascadae* | Ranidae | Larvae | Laboratory | 21 | Nitrogenous compounds | pH | No pollutant | pH=7 | 4 | 0.99 | 0.00 | -1.18 | 0.26 | -1.18 | 0.26 | -2.51 | 1.20 | Hatch & Blaustein, 2000 |
| *Rana cascadae* | Ranidae | Larvae | Laboratory | 21 |  |  | No pollutant | pH=5 | 4 | 1.00 | 0.00 |  |  |  |  |  |  | Hatch & Blaustein, 2000 |
| *Rana cascadae* | Ranidae | Larvae | Laboratory | 21 |  |  | High pollutant | pH=7 | 4 | 1.00 | 0.00 |  |  |  |  |  |  | Hatch & Blaustein, 2000 |
| *Rana cascadae* | Ranidae | Larvae | Laboratory | 21 |  |  | High pollutant | pH=5 | 4 | 0.70 | 0.20 |  |  |  |  |  |  | Hatch & Blaustein, 2000 |
| *Rana cascadae* | Ranidae | Larvae | Laboratory | 21 | Nitrogenous compounds | UV radiation | No pollutant | No UV radiation | 4 | 0.99 | 0.00 | 0.05 | 0.25 | -0.91 | 0.26 | -0.10 | 1.00 | Hatch & Blaustein, 2000 |
| *Rana cascadae* | Ranidae | Larvae | Laboratory | 21 |  |  | No pollutant | UV radiation | 4 | 0.90 | 0.12 |  |  |  |  |  |  | Hatch & Blaustein, 2000 |
| *Rana cascadae* | Ranidae | Larvae | Laboratory | 21 |  |  | Low pollutant | No UV radiation | 4 | 1.00 | 0.00 |  |  |  |  |  |  | Hatch & Blaustein, 2000 |
| *Rana cascadae* | Ranidae | Larvae | Laboratory | 21 |  |  | Low pollutant | UV radiation | 4 | 0.90 | 0.12 |  |  |  |  |  |  | Hatch & Blaustein, 2000 |
| *Rana cascadae* | Ranidae | Larvae | Laboratory | 21 | Nitrogenous compounds | UV radiation | No pollutant | No UV radiation | 4 | 0.99 | 0.00 | 0.26 | 0.25 | -0.78 | 0.25 | 0.31 | 1.00 | Hatch & Blaustein, 2000 |
| *Rana cascadae* | Ranidae | Larvae | Laboratory | 21 |  |  | No pollutant | UV radiation | 4 | 0.90 | 0.12 |  |  |  |  |  |  | Hatch & Blaustein, 2000 |
| *Rana cascadae* | Ranidae | Larvae | Laboratory | 21 |  |  | High pollutant | No UV radiation | 4 | 1.00 | 0.00 |  |  |  |  |  |  | Hatch & Blaustein, 2000 |
| *Rana cascadae* | Ranidae | Larvae | Laboratory | 21 |  |  | High pollutant | UV radiation | 4 | 0.94 | 0.10 |  |  |  |  |  |  | Hatch & Blaustein, 2000 |
| *Lithobates sphenocephalus* | Ranidae | Larvae | Laboratory | 105 | Nitrogenous compounds | Wastewater contaminants | No pollutant | No pollutant | 3 | 0.83 | 0.19 | -0.94 | 0.34 | -0.94 | 0.34 | -0.13 | 1.33 | Ortiz-Santaliestra & Sparling, 2007 |
| *Lithobates sphenocephalus* | Ranidae | Larvae | Laboratory | 105 |  |  | No pollutant | Low pollutant | 3 | 0.55 | 0.28 |  |  |  |  |  |  | Ortiz-Santaliestra & Sparling, 2007 |
| *Lithobates sphenocephalus* | Ranidae | Larvae | Laboratory | 105 |  |  | Pollutant | No pollutant | 3 | 0.55 | 0.21 |  |  |  |  |  |  | Ortiz-Santaliestra & Sparling, 2007 |
| *Lithobates sphenocephalus* | Ranidae | Larvae | Laboratory | 105 |  |  | Pollutant | Low pollutant | 3 | 0.23 | 0.10 |  |  |  |  |  |  | Ortiz-Santaliestra & Sparling, 2007 |
| *Lithobates sphenocephalus* | Ranidae | Larvae | Laboratory | 105 | Nitrogenous compounds | Wastewater contaminants | No pollutant | No pollutant | 3 | 0.83 | 0.19 | -0.87 | 0.34 | -2.54 | 0.40 | 0.58 | 1.35 | Ortiz-Santaliestra & Sparling, 2007 |
| *Lithobates sphenocephalus* | Ranidae | Larvae | Laboratory | 105 |  |  | No pollutant | High pollutant | 3 | 0.15 | 0.12 |  |  |  |  |  |  | Ortiz-Santaliestra & Sparling, 2007 |
| *Lithobates sphenocephalus* | Ranidae | Larvae | Laboratory | 105 |  |  | Pollutant | No pollutant | 3 | 0.55 | 0.21 |  |  |  |  |  |  | Ortiz-Santaliestra & Sparling, 2007 |
| *Lithobates sphenocephalus* | Ranidae | Larvae | Laboratory | 105 |  |  | Pollutant | High pollutant | 3 | 0.01 | 0.00 |  |  |  |  |  |  | Ortiz-Santaliestra & Sparling, 2007 |
| *Hyla versicolor* | Hylidae | Larvae | Mesocosms | 23 | Pesticides | Predators | No pollutant | No predator | 5 | 0.71 | 0.20 | 0.14 | 0.20 | -1.81 | 0.22 | 1.45 | 0.85 | Relyea *et al.*, 2005 |
| *Hyla versicolor* | Hylidae | Larvae | Mesocosms | 23 |  |  | No pollutant | Predator | 5 | 0.09 | 0.20 |  |  |  |  |  |  | Relyea *et al.*, 2005 |
| *Hyla versicolor* | Hylidae | Larvae | Mesocosms | 23 |  |  | Pollutant | No predator | 5 | 0.57 | 0.30 |  |  |  |  |  |  | Relyea *et al.*, 2005 |
| *Hyla versicolor* | Hylidae | Larvae | Mesocosms | 23 |  |  | Pollutant | Predator | 5 | 0.30 | 0.22 |  |  |  |  |  |  | Relyea *et al.*, 2005 |
| *Hyla versicolor* | Hylidae | Larvae | Mesocosms | 23 | Pesticides | Predators | No pollutant | No predator | 5 | 0.71 | 0.20 | -0.24 | 0.20 | -2.93 | 0.25 | 1.68 | 0.87 | Relyea *et al.*, 2005 |
| *Hyla versicolor* | Hylidae | Larvae | Mesocosms | 23 |  |  | No pollutant | Predator | 5 | 0.09 | 0.20 |  |  |  |  |  |  | Relyea *et al.*, 2005 |
| *Hyla versicolor* | Hylidae | Larvae | Mesocosms | 23 |  |  | Pollutant | No predator | 5 | 0.53 | 0.10 |  |  |  |  |  |  | Relyea *et al.*, 2005 |
| *Hyla versicolor* | Hylidae | Larvae | Mesocosms | 23 |  |  | Pollutant | Predator | 5 | 0.19 | 0.10 |  |  |  |  |  |  | Relyea *et al.*, 2005 |
| *Lithobates pipiens* | Ranidae | Larvae | Mesocosms | 23 | Pesticides | Predators | No pollutant | No predator | 5 | 0.99 | 0.05 | 1.52 | 0.21 | -2.35 | 0.23 | 3.26 | 1.07 | Relyea *et al.*, 2005 |
| *Lithobates pipiens* | Ranidae | Larvae | Mesocosms | 23 |  |  | No pollutant | Predator | 5 | 0.19 | 0.05 |  |  |  |  |  |  | Relyea *et al.*, 2005 |
| *Lithobates pipiens* | Ranidae | Larvae | Mesocosms | 23 |  |  | Pollutant | No predator | 5 | 0.97 | 0.05 |  |  |  |  |  |  | Relyea *et al.*, 2005 |
| *Lithobates pipiens* | Ranidae | Larvae | Mesocosms | 23 |  |  | Pollutant | Predator | 5 | 0.82 | 0.37 |  |  |  |  |  |  | Relyea *et al.*, 2005 |
| *Lithobates pipiens* | Ranidae | Larvae | Mesocosms | 23 | Pesticides | Predators | No pollutant | No predator | 5 | 0.99 | 0.05 | -0.40 | 0.20 | -4.95 | 0.35 | -0.48 | 0.81 | Relyea *et al.*, 2005 |
| *Lithobates pipiens* | Ranidae | Larvae | Mesocosms | 23 |  |  | No pollutant | Predator | 5 | 0.33 | 0.22 |  |  |  |  |  |  | Relyea *et al.*, 2005 |
| *Lithobates pipiens* | Ranidae | Larvae | Mesocosms | 23 |  |  | Pollutant | No predator | 5 | 0.97 | 0.05 |  |  |  |  |  |  | Relyea *et al.*, 2005 |
| *Lithobates pipiens* | Ranidae | Larvae | Mesocosms | 23 |  |  | Pollutant | Predator | 5 | 0.24 | 0.12 |  |  |  |  |  |  | Relyea *et al.*, 2005 |
| *Lithobates pipiens* | Ranidae | Larvae | Mesocosms | 23 | Pesticides | Predators | No pollutant | No predator | 5 | 0.99 | 0.05 | -1.34 | 0.21 | -5.23 | 0.37 | 1.99 | 0.90 | Relyea *et al.*, 2005 |
| *Lithobates pipiens* | Ranidae | Larvae | Mesocosms | 23 |  |  | No pollutant | Predator | 5 | 0.19 | 0.05 |  |  |  |  |  |  | Relyea *et al.*, 2005 |
| *Lithobates pipiens* | Ranidae | Larvae | Mesocosms | 23 |  |  | Pollutant | No predator | 5 | 0.69 | 0.20 |  |  |  |  |  |  | Relyea *et al.*, 2005 |
| *Lithobates pipiens* | Ranidae | Larvae | Mesocosms | 23 |  |  | Pollutant | Predator | 5 | 0.14 | 0.12 |  |  |  |  |  |  | Relyea *et al.*, 2005 |
| *Lithobates pipiens* | Ranidae | Larvae | Mesocosms | 23 | Pesticides | Predators | No pollutant | No predator | 5 | 0.99 | 0.05 | -1.53 | 0.21 | -3.65 | 0.28 | 0.53 | 0.81 | Relyea *et al.*, 2005 |
| *Lithobates pipiens* | Ranidae | Larvae | Mesocosms | 23 |  |  | No pollutant | Predator | 5 | 0.33 | 0.22 |  |  |  |  |  |  | Relyea *et al.*, 2005 |
| *Lithobates pipiens* | Ranidae | Larvae | Mesocosms | 23 |  |  | Pollutant | No predator | 5 | 0.69 | 0.20 |  |  |  |  |  |  | Relyea *et al.*, 2005 |
| *Lithobates pipiens* | Ranidae | Larvae | Mesocosms | 23 |  |  | Pollutant | Predator | 5 | 0.12 | 0.10 |  |  |  |  |  |  | Relyea *et al.*, 2005 |
| *Pseudacris regilla* | Hylidae | Larvae | Laboratory | 7 | Nitrogenous compounds | Mold | No pollutant | No mold | 5 | 0.96 | 0.05 | -0.31 | 0.20 | 0.37 | 0.20 | -0.98 | 0.82 | Romansic *et al.*, 2006 |
| *Pseudacris regilla* | Hylidae | Larvae | Laboratory | 7 |  |  | No pollutant | Mold | 5 | 1.00 | 0.00 |  |  |  |  |  |  | Romansic *et al.*, 2006 |
| *Pseudacris regilla* | Hylidae | Larvae | Laboratory | 7 |  |  | Low pollutant | No mold | 5 | 0.97 | 0.05 |  |  |  |  |  |  | Romansic *et al.*, 2006 |
| *Pseudacris regilla* | Hylidae | Larvae | Laboratory | 7 |  |  | Low pollutant | Mold | 5 | 0.96 | 0.05 |  |  |  |  |  |  | Romansic *et al.*, 2006 |
| *Pseudacris regilla* | Hylidae | Larvae | Laboratory | 7 | Nitrogenous compounds | Mold | No pollutant | No mold | 5 | 0.96 | 0.05 | 0.75 | 0.20 | 0.75 | 0.20 | -1.49 | 0.86 | Romansic *et al.*, 2006 |
| *Pseudacris regilla* | Hylidae | Larvae | Laboratory | 7 |  |  | No pollutant | Mold | 5 | 1.00 | 0.00 |  |  |  |  |  |  | Romansic *et al.*, 2006 |
| *Pseudacris regilla* | Hylidae | Larvae | Laboratory | 7 |  |  | High pollutant | No mold | 5 | 1.00 | 0.00 |  |  |  |  |  |  | Romansic *et al.*, 2006 |
| *Pseudacris regilla* | Hylidae | Larvae | Laboratory | 7 |  |  | High pollutant | Mold | 5 | 1.00 | 0.00 |  |  |  |  |  |  | Romansic *et al.*, 2006 |
| *Rana aurora* | Ranidae | Larvae | Laboratory | 7 | Nitrogenous compounds | Mold | No pollutant | No mold | 5 | 0.92 | 0.07 | -0.09 | 0.20 | -1.29 | 0.21 | 1.79 | 0.88 | Romansic *et al.*, 2006 |
| *Rana aurora* | Ranidae | Larvae | Laboratory | 7 |  |  | No pollutant | Mold | 5 | 0.62 | 0.16 |  |  |  |  |  |  | Romansic *et al.*, 2006 |
| *Rana aurora* | Ranidae | Larvae | Laboratory | 7 |  |  | Low pollutant | No mold | 5 | 0.78 | 0.07 |  |  |  |  |  |  | Romansic *et al.*, 2006 |
| *Rana aurora* | Ranidae | Larvae | Laboratory | 7 |  |  | Low pollutant | Mold | 5 | 0.73 | 0.19 |  |  |  |  |  |  | Romansic *et al.*, 2006 |
| *Rana aurora* | Ranidae | Larvae | Laboratory | 7 | Nitrogenous compounds | Mold | No pollutant | No mold | 5 | 0.92 | 0.07 | 0.13 | 0.20 | -1.29 | 0.21 | 2.53 | 0.96 | Romansic *et al.*, 2006 |
| *Rana aurora* | Ranidae | Larvae | Laboratory | 7 |  |  | No pollutant | Mold | 5 | 0.62 | 0.16 |  |  |  |  |  |  | Romansic *et al.*, 2006 |
| *Rana aurora* | Ranidae | Larvae | Laboratory | 7 |  |  | High pollutant | No mold | 5 | 0.79 | 0.08 |  |  |  |  |  |  | Romansic *et al.*, 2006 |
| *Rana aurora* | Ranidae | Larvae | Laboratory | 7 |  |  | High pollutant | Mold | 5 | 0.78 | 0.12 |  |  |  |  |  |  | Romansic *et al.*, 2006 |
| *Ambystoma gracile* | Ambystomatidae | Larvae | Laboratory | 7 | Nitrogenous compounds | Mold | No pollutant | No mold | 5 | 1.00 | 0.00 | 1.12 | 0.21 | -1.12 | 0.21 | 2.23 | 0.92 | Romansic *et al.*, 2006 |
| *Ambystoma gracile* | Ambystomatidae | Larvae | Laboratory | 7 |  |  | No pollutant | Mold | 5 | 0.94 | 0.05 |  |  |  |  |  |  | Romansic *et al.*, 2006 |
| *Ambystoma gracile* | Ambystomatidae | Larvae | Laboratory | 7 |  |  | Low pollutant | No mold | 5 | 1.00 | 0.00 |  |  |  |  |  |  | Romansic *et al.*, 2006 |
| *Ambystoma gracile* | Ambystomatidae | Larvae | Laboratory | 7 |  |  | Low pollutant | Mold | 5 | 1.00 | 0.00 |  |  |  |  |  |  | Romansic *et al.*, 2006 |
| *Ambystoma gracile* | Ambystomatidae | Larvae | Laboratory | 7 | Nitrogenous compounds | Mold | No pollutant | No mold | 5 | 1.00 | 0.00 | 1.12 | 0.21 | -1.12 | 0.21 | 2.23 | 0.92 | Romansic *et al.*, 2006 |
| *Ambystoma gracile* | Ambystomatidae | Larvae | Laboratory | 7 |  |  | No pollutant | Mold | 5 | 0.94 | 0.05 |  |  |  |  |  |  | Romansic *et al.*, 2006 |
| *Ambystoma gracile* | Ambystomatidae | Larvae | Laboratory | 7 |  |  | High pollutant | No mold | 5 | 1.00 | 0.00 |  |  |  |  |  |  | Romansic *et al.*, 2006 |
| *Ambystoma gracile* | Ambystomatidae | Larvae | Laboratory | 7 |  |  | High pollutant | Mold | 5 | 1.00 | 0.00 |  |  |  |  |  |  | Romansic *et al.*, 2006 |
| *Rana temporaria* | Ranidae | Larvae | Laboratory | 3 | Pesticides | Predators | No pollutant | No predator | 5 | 1.00 | 0.00 | -1.56 | 0.22 | -16.59 | 1.92 | -3.11 | 1.04 | Mandrillon & Saglio, 2007 |
| *Rana temporaria* | Ranidae | Larvae | Laboratory | 3 |  |  | No pollutant | Predator | 5 | 0.61 | 0.04 |  |  |  |  |  |  | Mandrillon & Saglio, 2007 |
| *Rana temporaria* | Ranidae | Larvae | Laboratory | 3 |  |  | Very low pollutant | No predator | 5 | 1.00 | 0.00 |  |  |  |  |  |  | Mandrillon & Saglio, 2007 |
| *Rana temporaria* | Ranidae | Larvae | Laboratory | 3 |  |  | Very low pollutant | Predator | 5 | 0.53 | 0.02 |  |  |  |  |  |  | Mandrillon & Saglio, 2007 |
| *Rana temporaria* | Ranidae | Larvae | Laboratory | 3 | Pesticides | Predators | No pollutant | No predator | 5 | 1.00 | 0.00 | 0.27 | 0.20 | -15.36 | 1.68 | 0.54 | 0.81 | Mandrillon & Saglio, 2007 |
| *Rana temporaria* | Ranidae | Larvae | Laboratory | 3 |  |  | No pollutant | Predator | 5 | 0.61 | 0.04 |  |  |  |  |  |  | Mandrillon & Saglio, 2007 |
| *Rana temporaria* | Ranidae | Larvae | Laboratory | 3 |  |  | Low pollutant | No predator | 5 | 1.00 | 0.00 |  |  |  |  |  |  | Mandrillon & Saglio, 2007 |
| *Rana temporaria* | Ranidae | Larvae | Laboratory | 3 |  |  | Low pollutant | Predator | 5 | 0.63 | 0.02 |  |  |  |  |  |  | Mandrillon & Saglio, 2007 |
| *Rana temporaria* | Ranidae | Larvae | Laboratory | 3 | Pesticides | Predators | No pollutant | No predator | 5 | 1.00 | 0.00 | 2.33 | 0.23 | -12.71 | 1.21 | 4.67 | 1.34 | Mandrillon & Saglio, 2007 |
| *Rana temporaria* | Ranidae | Larvae | Laboratory | 3 |  |  | No pollutant | Predator | 5 | 0.61 | 0.04 |  |  |  |  |  |  | Mandrillon & Saglio, 2007 |
| *Rana temporaria* | Ranidae | Larvae | Laboratory | 3 |  |  | High pollutant | No predator | 5 | 1.00 | 0.00 |  |  |  |  |  |  | Mandrillon & Saglio, 2007 |
| *Rana temporaria* | Ranidae |  |  | 3 |  |  | High pollutant | Predator | 5 | 0.73 | 0.02 |  |  |  |  |  |  | Mandrillon & Saglio, 2007 |
| *Lithobates pipiens* | Ranidae | Larvae | Laboratory | 24 | Wastewater contaminants | Wastewater contaminants | No pollutant | No pollutant | 3 | 1.00 | 0.00 | -0.37 | 0.33 | 0.37 | 0.33 | 0.74 | 1.36 | Fraker & Smith, 2004 |
| *Lithobates pipiens* | Ranidae | Larvae | Laboratory | 24 |  |  | No pollutant | Low pollutant | 3 | 1.00 | 0.00 |  |  |  |  |  |  | Fraker & Smith, 2004 |
| *Lithobates pipiens* | Ranidae | Larvae | Laboratory | 24 |  |  | High pollutant | No pollutant | 3 | 0.93 | 0.12 |  |  |  |  |  |  | Fraker & Smith, 2004 |
| *Lithobates pipiens* | Ranidae | Larvae | Laboratory | 24 |  |  | High pollutant | Low pollutant | 3 | 1.00 | 0.00 |  |  |  |  |  |  | Fraker & Smith, 2004 |
| *Lithobates pipiens* | Ranidae | Larvae | Laboratory | 24 | Wastewater contaminants | Wastewater contaminants | No pollutant | No pollutant | 3 | 1.00 | 0.00 | -0.37 | 0.33 | 0.37 | 0.33 | 0.74 | 1.36 | Fraker & Smith, 2004 |
| *Lithobates pipiens* | Ranidae | Larvae | Laboratory | 24 |  |  | No pollutant | High pollutant | 3 | 1.00 | 0.00 |  |  |  |  |  |  | Fraker & Smith, 2004 |
| *Lithobates pipiens* | Ranidae | Larvae | Laboratory | 24 |  |  | High pollutant | No pollutant | 3 | 0.93 | 0.12 |  |  |  |  |  |  | Fraker & Smith, 2004 |
| *Lithobates pipiens* | Ranidae | Larvae | Laboratory | 24 |  |  | High pollutant | High pollutant | 3 | 1.00 | 0.00 |  |  |  |  |  |  | Fraker & Smith, 2004 |

References cited.

Boone MD, Semlitsch RD (2003) Interaction of bullfrog tadpole predators and an insecticide: predation release and facilitation. *Oecologia*, **42**, 610-616.

Burgett AA, Wright CD, Smith GR, Fortune DT, Johnson SL (2007) Impact of ammonium nitrate on wood frog (*Rana sylvativa*) tadpoles: effect on survivorship and behavior. *Herpetological Conservation and Biology*, **2**, 29-34.

Carr JA, Gentles A, Smith EE *et al*. (2003) Response of larval *Xenopus laevis* to atrazine: assessment of growth, metamorphosis and gonadal and laryngeal morphology. *Environmental Toxicology and Chemistry*, **22**, 396-405.

Chen TH, Gross JA, Karasov WH (2006) Sublethal effects of lead on Northern leopard frog (*Rana pipiens*) tadpoles. *Environmental Toxicology and Chemistry*, **25**, 1383-1389.

Chen TH, Gross JA, Karasov WH (2007) Adverse effects of chronic copper exposure in larval Northern leopard frog (*Rana pipiens*). *Environmental Toxicology and Chemistry*, **26**, 1470-1475.

De Wiejer P, Watt PJ, Oldham RS (2003) Amphibian decline and aquatic pollution: effects of nitrogenous fertiliser on survival and development of larvae of the frog *Rana temporaria*. *Applied Herpetology*, **1**, 3-12.

Dougherty CK, Smith GR (2006) Acute effects of road de-icers on the tadpoles of three anurans. *Applied Herpetology*, **3**, 87-93.

Egea-Serrano A, Tejedo M, Torralva M (2009) Populational divergence in the impact of three nitrogenous compounds and their combination on larvae of the frog *Pelophylax perezi* (Seoane, 1885). *Chemosphere*, **76**, 869-877.

Egea-Serrano A (2010) *Aspectos relevantes en la conservación de anfibios en la Región de Murcia: efectos de la contaminación por fertilizantes sobre Pelophylax perezi (Seoane, 1885) [Relevant aspects in amphibian conservation in the province of Murcia: effect of fertilizer pollution on Pelophylax perezi (Seoane, 1885)*. PhD Thesis. Universidad de Murcia, Murcia, 366 pp.

Fraker SL, Smith GR (2004) Direct and interactive effects of ecologically relevant concentrations of organic wastewater contaminants on *Rana pipiens* tadpoles. *Environmental Toxicology*, **19**, 250-256.

Fraker SL, Smith GR (2005) Effects of two organic wastewater contaminants on *Xenopus laevis* tadpoles. *Applied Herpetology*, **2**, 381-388.

Greulich K, Pfluggmacher S (2003) Differences in susceptibility of various life stages of amphibians to pesticide exposure. *Aquatic Toxicology*, **65**, 329-336.

Griffis-Kyle KL (2007) Sublethal effects of nitrite on eastern tiger salamander (*Ambystoma tigrinum tigrinum*) and wood frog (*Rana sylvatica*) embryos and larvae: implications for field populations. *Aquatic Ecology*, **41**, 119-127.

Hatch AC, Blaustein AR (2000) Combined effects of UV-B, nitrate and low pH reduce the survival and activity levels of larval cascade frogs (*Rana cascadae*). *Archives of Environmental Contamination and Toxicology*, **39**, 494-499.

Karraker NE, Gibbs JP, Vonesh JR (2008) Impact of road deicing salt on the demography of vernal pool-breeding amphibians. *Ecological Applications*, **18**, 724-734.

Laposata MM, Dunson WA (1998) Effects of boron and nitrate on hatching success of amphibian eggs. *Archives of Environmental Contamination and Toxicology*, **35**, 615-619.

Mandrillon AL, Saglio P (2007) Waterborne amitrole affects the predator prey relationship between common frog tadpoles (*Rana temporaria*) and larval spotted salamander (*Salamandra salamandra*). *Archives of Environmental Contamination and Toxicology*, **53**, 233-240.

Mandrillon AL, Saglio P (2009) Effects of single and combined embryonic exposures to herbicide and conspecific chemical alarm cues on hatching and larval traits in the common frog (*Rana temporaria*). *Archives of Environmental Contamination and Toxicology*, **56**, 566-576.

Metts BS, Hopkins WA, Nestor JP (2005) Interaction of an insecticide with larval density in pond-breeding salamanders (*Ambystoma*). *Freshwater Biology*, **50**, 685-696.

Ortiz ME, Marco A, Saiz N, Lizana M (2004) Impact of ammonium nitrate on growth and survival of six European amphibians. *Archives of Environmental Contamination and Toxicology*, **47**, 234-239.

Ortiz-Santaliestra M, Marco A, Fernández MJ, Lizana M (2006) Influence of developmental stage on sensitivity to ammonium nitrate of aquatic stages of amphibians. *Environmental Toxicology and Chemistry*, **25**, 105-111.

Ortiz-Santaliestra M, Marco A, Fernández-Benéitez MJ, Lizana M (2007) Effects of ammonium nitrate exposure and water acidification on dwarf newt: the protective effect of oviposition behabiour on embryonic survival. *Aquatic Toxicology*, **85**, 251-257.

Ortiz-Santaliestra M, Sparling D (2007) Alteration of larval development and metamorphosis by nitrate and perchlorate in Southern leopard frogs (*Rana sphenocephala*). *Archives of Environmental Contamination and Toxicology*, **53**, 639-646.

Peltzer PM, Lajmanovich RC, Attademo AM, Beltzer AH (2006) Diversity of anurans across agricultural ponds in Argentina. *Biodiversity and Conservation*, **15**, 3499-3513.

Relyea R (2005) The impact of insecticides and herbicides on the biodiversity and productivity of aquatic communities. *Ecological Applications*, **15**, 618-627.

Relyea R (2005) The lethal impact of roundup on aquatic and terrestrial amphibians. *Ecological Applications*, **15**, 1118-1124.

Relyea R (2006) The effects of pesticides, pH, and predatory stress on amphibians under mesocosm conditions. *Ecotoxicology*, **15**, 503-511.

Relyea R (2009) A cocktail of contaminants: how mixtures of pesticides at low concentrations affect aquatic communities. *Oecologia*, **159**, 363-376.

Relyea R, Mills N (2001) Predator-induced stress makes the pesticide carbaryl more deadly to gray treefrog tadpoles. *Proceedings of the National Academy of Sciences of the United States of America*, **27**, 2491-2496.

Relyea R, Schoeppner NM, Hoverman JT (2005) Pesticides and amphibians: the importance of community context. *Ecological Applications*, **15**, 1125-1134.

Rohr JR, Elskus AA, Shepherd BS, Crowley PH, McCarthy TM (2003) Lethal and sublethal effects of atrazine, carbaryl endosulfan and octylphenol on the streamside salamander (*Ambystoma barbouri*). *Environmental Toxicology and Chemistry*, **22**, 2385-2392.

Romansic JM, Diez KA, Higashi EM, Blaustein AR (2006) Effects of nitrate on the pathogenic water mold *Saprolegnia* on survival of amphibian larvae. *Diseases of Aquatic Organisms*, **68**, 235-243.

Sanzo D, Hecnar SJ (2006) Effects of road de-icing salt (NaCl) on larval wood frogs (*Rana sylvatica*). *Environmental Pollution*, **140**, 247-256.

Schuytema GS, Nebeker AV (1999a) Effects of ammonium nitrate, sodium nitrate and urea on red-legged frogs, Pacific treefrogs and African clawed frogs. *Bulletin of Environmental Contamination and Toxicology*, **63**, 357-364.

Schuytema GS, Nebeker AV (1999b) Compatative effects of ammonium and nitrate compounds on Pacific treefrogs and African clawed frog embryos. *Archives of Environmental Contamination and Toxicology*, **36**, 200-206.

Smith GR (2001) Effects of acute exposure to commercial formulation of glyphosate on the tadpoles of two species of anurans. *Bulletin of Environmental Contamination and Toxicology*, **67**, 483-488.

Smith GR (2007) Lack of effect of nitrate, nitrite and phosphate on wood frog (*Rana sylvatica*) tadpoles. *Applied Herpetology*, **4**, 287-291.

Smith GR, Burgett AA (2005) Effects of three organic wastewater contaminants on American toad, *Bufo americanus*, tadpoles. *Ecotoxicology*, **14**, 477-482.

Smith GR, Temple KG, Dingfelder HA, Vaala DA (2006) Effects of nitrate on the interactions of the tadpoles of two ranids (*Rana clamitans* and *Rana catesbeiana*). *Aquatic Ecology*, **40**, 125-130.

Smith GR, Temple KG, Vaala DA, Dingfelder HA (2005) Effects of nitrate on the tadpoles of two ranids (*Rana catebeiana* and *R. clamitans*). *Archives of Environmental Contamination and Toxicology*, **49**, 559-562.

Smith GR, Vaala DA, Dingfelder HA, Temple KG (2004) Effects of nitrite on bullfrog (*Rana catesbeiana*) tadpoles from central Ohio, USA. *Bulletin of Environmental Contamination and Toxicology*, **72**, 1012-1016.

Snodgrass JW, Casey RE, Joseph D, Simon JA (2008) Microcosm investigations of stormwater pond sediment toxicity to embryonic and larval amphibians: Variation in sensitivity among species. *Environmental Pollution*, **154**, 291-297.

Unrine J, Jagoe CH, Hopkins WA, Brant HA (2004) Adverse effects of ecologically relevant dietary mercury exposure in southern leopard frog (*Rana sphenocephala*) larvae. *Environmental Toxicology and Chemistry*, **23**, 2964-2970.

Vaala DA, Smith GR, Temple KG, Dingfelder HA (2004) No effect of nitrate on gray treefrog (*Hyla versicolor*) tadpoles. *Applied Herpetology*, **1**, 265-269.

Watt PJ, Jarvis P (1997) Survival analysis in palmate newts exposed to ammonium nitrate agricultural fertilizer. *Ecotoxicology*, **6**, 355-362.

Watt PJ, Oldham RS (1995) The effect of ammonium nitrate on the feeding and development of larvae of the smooth newt, *Triturus vulgaris* (L.), and on the behaviour of its food source, *Daphnia. Freshwater Biology*, **33**, 319-324.

Widder PD, Bidwell JR (2006) Cholinesterase activity and behaviour in chlorpyrifos-exposed *Rana sphenocephala* tadpoles. *Environmental Toxicology and Chemistry*, **25**, 2446-2454.

Widder PM, Bidwell JR (2008) Tadpole size, cholinesterase activity and swim speed in four forg species after exposure to sub-lethal concentration of chlorpyrifos. *Aquatic Toxicology*, **88**, 9-18.
